# Supplementary material for: Exploitation of phylum-spanning omics resources reveals complexity in the nematode FLP signalling system and provides insights into flp-gene evolution
Source: BMC Genomics. 2024 Dec 19;25:1220. doi: 10.1186/s12864-024-11111-6 (PMC11658156; doi:10.1186/s12864-024-11111-6)
Supplement: Supplementary file 4 — Supplementary Material 4 [file 12864_2024_11111_MOESM4_ESM.pdf]

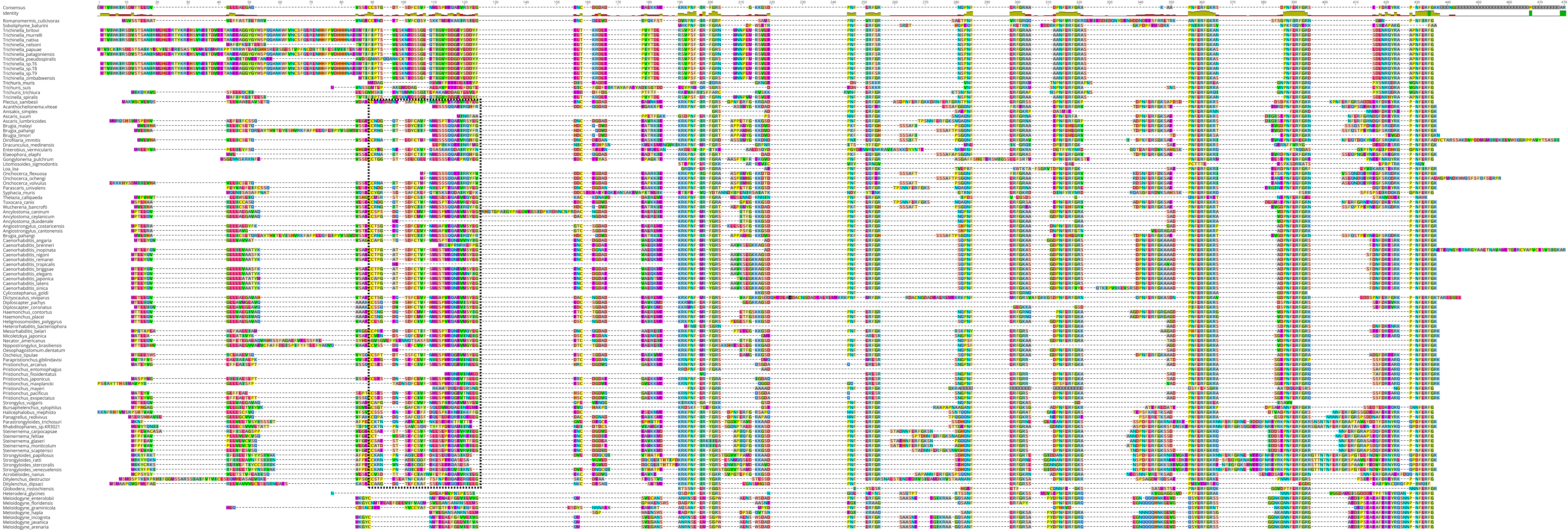
Consensus
Identity
Romanomermis_culicivorax
Sobolphyme_baturini
Trichinella_britovi
Trichinella_murrelli
Trichinella_nativa
Trichinella_nelsoni
Trichinella_papuae
Trichinella_patagoniensis
Trichinella_pseudospiralis
Trichinella_sp.T6
Trichinella_sp.T8
Trichinella_sp.T9
Trichinella_zimbabwensis
Trichuris_muris
Trichuris_suis
Trichuris_trichiura
Tricinella_spiralis
Plectus_sambesii
Acanthocheilonema.viteae
Anisakis_simplex
Ascaris_suum
Ascaris_lumbricoides
Brugia_malayi
Brugia_pahangi
Brugia_timori
Dirofilaria_immitis
Dracunculus_medinensis
Enterobius_vermicularis
Elaeophora_elaphi
Gongylonema_pulchrum
Litomosoides_sigmodontis
Loa_loa
Onchocerca_flexuosa
Onchocerca_ochengi
Onchocerca_volvulus
Parascaris_univalens
Syphacia_muris
Thelazia_callipaeda
Toxocara_canis
Wuchereria_bancrofti
Ancylostoma_caninum
Ancylostoma_ceylanicum
Ancylostoma_duodenale
Angiostrongylus_costaricensis
Angiostrongylus_cantonensis
Brugia_pahangi
Caenorhabditis_angaria
Caenorhabditis_brenneri
Caenorhabditis_inopinata
Caenorhabditis_nigoni
Caenorhabditis_remanei
Caenorhabditis_tropicalis
Caenorhabditis_briggsae
Caenorhabditis_elegans
Caenorhabditis_japonica
Caenorhabditis_latens
Caenorhabditis_sinica
Cylicostephanus_goldi
Dictyocaulus_viviparus
Diploscapter_pachys
Diploscapter_coronatus
Haemonchus_contortus
Haemonchus_placei
Heligmosomoides_polygyrus
Heterorhabditis_bacteriophora
Mesorhabditis_belari
Micoletzkya_japonica
Necator_americanus
Nippostrongylus_brasiliensis
Oesophagostomum.dentatum
Oscheius_tipulae
Parapristionchus.giblindavisi
Pristionchus_arcanus
Pristionchus_entomophagus
Pristionchus_fissidentatus
Pristionchus_japonicus
Pristionchus_maxplancki
Pristionchus_mayeri
Pristionchus_pacificus
Pristionchus_exspectatus
Strongylus_vulgaris
Bursaphelenchus_xylophilus
Halicephalobus_mephisto
Panagrellus_redivivus
Parastrongyloides_trichosuri
Rhabditophanes_sp.KR3021
Steinernema_carpocapsae
Steinernema_feltiae
Steinernema_glaseri
Steinernema_monticolum
Steinernema_scapterisci
Strongyloides_papillosus
Strongyloides_ratti
Strongyloides_stercoralis
Strongyloides_venezuelensis
Acrobeloides_nanus
Ditylenchus_destructor
Ditylenchus_dipsaci
Globodera_rostochiensis
Heterodera_glycines
Meloidogyne_enterolobii
Meloidogyne_floridensis
Meloidogyne_graminicola
Meloidogyne_hapla
Meloidogyne_incognita
Meloidogyne_javanica
Meloidogyne_arenaria

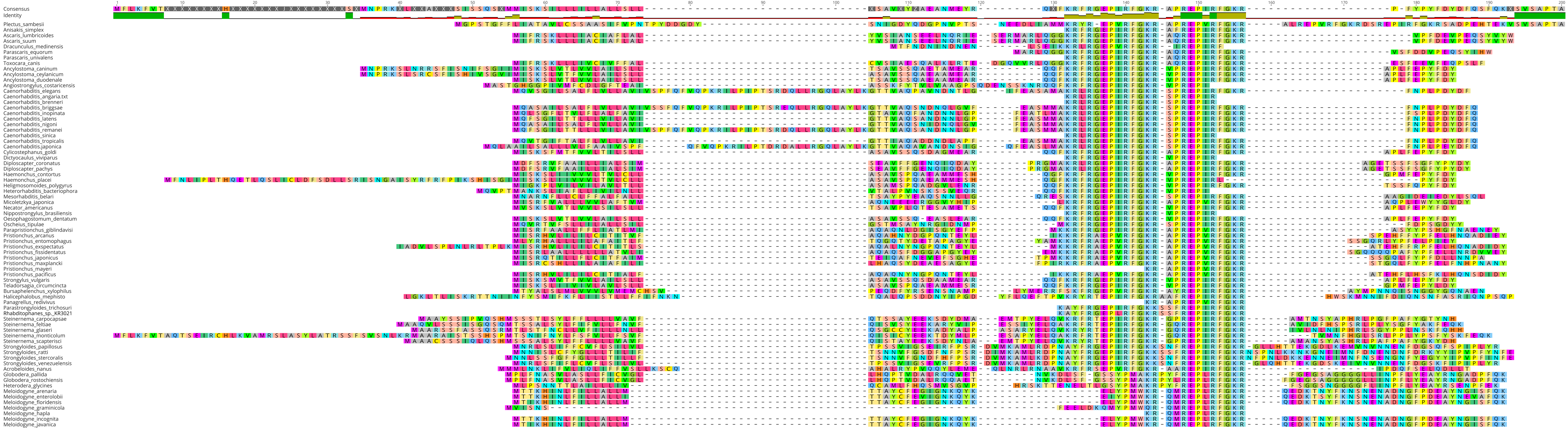
Consensus
Identity
Plectus_sambesii
Anisakis_simplex
Ascaris_lumbricoides
Ascaris_suum
Dracunculus_medinensis
Parascaris_equorum
Parascaris_univalens
Toxocara_canis
Ancylostoma_caninum
Ancylostoma_ceylanicum
Ancylostoma_duodenale
Angiostrongylus_costaricensis
Caenorhabditis_elegans
Caenorhabditis_angaria.txt
Caenorhabditis_brenneri
Caenorhabditis_briggsae
Caenorhabditis_inopinata
Caenorhabditis_latens
Caenorhabditis_nigoni
Caenorhabditis_remanei
Caenorhabditis_sinica
Caenorhabditis_tropicalis
Caenorhabditis_japonica
Cylicostephanus_goldi
Dictyocaulus_viviparus
Diploscapter_coronatus
Diploscapter_pachys
Haemonchus_contortus
Haemonchus_placei
Heligmosomoides_polygyrus
Heterorhabditis_bacteriophora
Mesorhabditis_belari
Micoletzkya_japonica
Necator_americanus
Nippostrongylus_brasiliensis
Oesophagostomum_dentatum
Oscheius_tipulae
Parapristionchus_giblindavisi
Pristionchus_arcanus
Pristionchus_entomophagus
Pristionchus_exspectatus
Pristionchus_fissidentatus
Pristionchus_japonicus
Pristionchus_maxplancki
Pristionchus_mayeri
Pristionchus_pacificus
Strongylus_vulgaris
Teladorsagia_circumcincta
Bursaphelenchus_xylophilus
Halicephalobus_mephisto
Panagrellus_redivivus
Parastrongyloides_trichosuri
Rhabditophanes_sp._KR3021
Steinernema_carpocapsae
Steinernema_feltiae
Steinernema_glaseri
Steinernema_monticolum
Steinernema_scapterisci
Strongyloides_papillosus
Strongyloides_ratti
Strongyloides_stercoralis
Strongyloides_venezuelensis
Acrobeloides_nanus
Globodera_pallida
Globodera_rostochiensis
Heterodera_glycines
Meloidogyne_arenaria
Meloidogyne_enterolobii
Meloidogyne_floridensis
Meloidogyne_graminicola
Meloidogyne_hapla
Meloidogyne_incognita
Meloidogyne_javanica

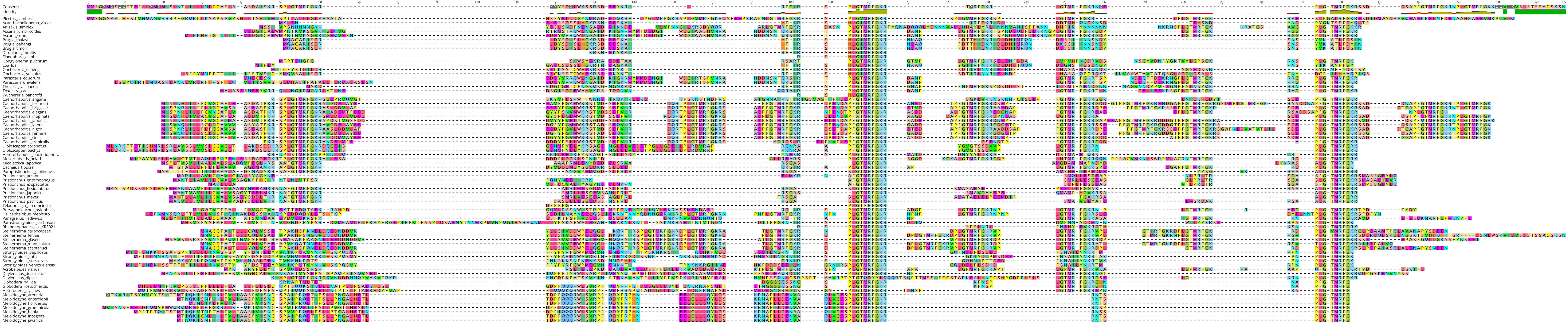

Consensus
Identity
Plectus_sambesii
Acanthocheilonema_viteae
Anisakis_simplex
Ascaris_lumbricoides
Ascaris_suum
Brugia_malayi
Brugia_pahangi
Brugia_timori
Dirofilaria_immitis
Elaeophora_elaphi
Gongylonema_pulchrum
Loa_loa
Onchocerca_ochengi
Onchocerca_volvulus
Parascaris_equorum
Parascaris_univalens
Thelazia_callipaeda
Toxocara_canis
Wuchereria_bancrofti
Caenorhabditis_angaria
Caenorhabditis_brenneri
Caenorhabditis_briggsae
Caenorhabditis_elegans
Caenorhabditis_inopinata
Caenorhabditis_japonica
Caenorhabditis_latens
Caenorhabditis_nigoni
Caenorhabditis_remanei
Caenorhabditis_sinica
Caenorhabditis_tropicalis
Diploscapter_coronatus
Diploscapter_pachys
Heterorhabditis_bacteriophora
Mesorhabditis_belari
Micoletzkya_japonica
Oscheius_tipulae
Parapristionchus_giblindavisi
Pristionchus_arcanus
Pristionchus_entomophagus
Pristionchus_exspectatus
Pristionchus_fissidentatus
Pristionchus_japonicus
Pristionchus_mayeri
Pristionchus_pacificus
Teladorsagia_circumcincta
Bursaphelenchus_xylophilus
Halicephalobus_mephisto
Panagrellus_redivivus
Parastrongyloides_trichosuri
Rhabditophanes_sp._KR3021
Steinernema_carpocapsae
Steinernema_feltiae
Steinernema_glaseri
Steinernema_monticolum
Steinernema_scapterisci
Strongyloides_papillosus
Strongyloides_ratti
Strongyloides_stercoralis
Strongyloides_venezuelensis
Acrobeloides_nanus
Ditylenchus_destructor
Ditylenchus_dipsaci
Globodera_pallida
Globodera_rostochiensis
Heterodera_glycines
Meloidogyne_arenaria
Meloidogyne_enterolobii
Meloidogyne_floridensis
Meloidogyne_graminicola
Meloidogyne_hapla
Meloidogyne_incognita
Meloidogyne_javanica

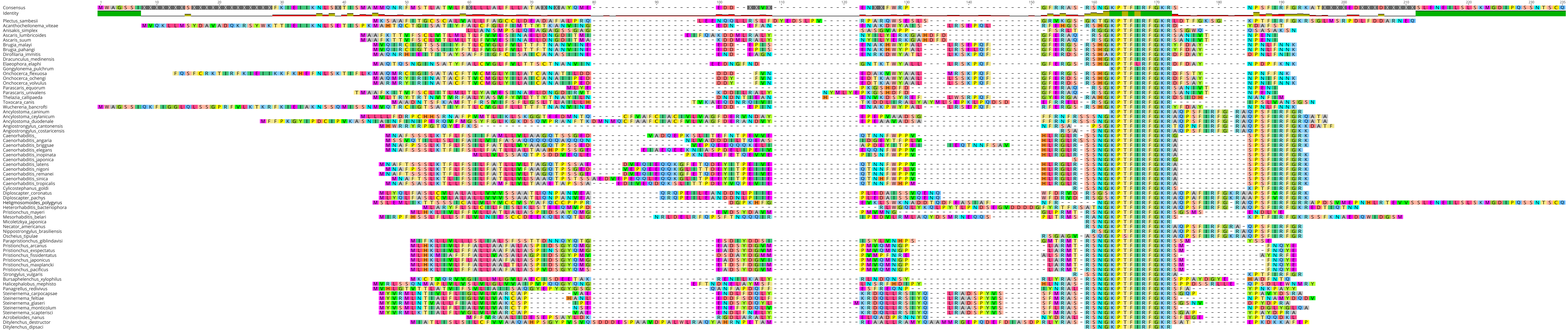
Consensus
Identity
Plectus_sambesii
Acanthocheilonema_viteae
Anisakis_simplex
Ascaris_lumbricoides
Ascaris_suum
Brugia_malayi
Brugia_pahangi
Dirofilaria_immitis
Dracunculus_medinensis
Elaeophora_elaphi
Gongylonema_pulchrum
Onchocerca_flexuosa
Onchocerca_ochengi
Onchocerca_volvulus
Parascaris_equorum
Parascaris_univalens
Thelazia_callipaeda
Toxocara_canis
Wuchereria_bancrofti
Ancylostoma_caninum
Ancylostoma_ceylanicum
Ancylostoma_duodenale
Angiostrongylus_cantonensis
Angiostrongylus_costaricensis
Caenorhabditis_
Caenorhabditis_angaria
Caenorhabditis_briggsae
Caenorhabditis_elegans
Caenorhabditis_inopinata
Caenorhabditis_japonica
Caenorhabditis_latens
Caenorhabditis_nigoni
Caenorhabditis_remanei
Caenorhabditis_sinica
Caenorhabditis_tropicalis
Cylicostephanus_goldi
Diploscapter_coronatus
Diploscapter_pachys
Heligmosomoides_polygyrus
Heterorhabditis_bacteriophora
Pristionchus_mayeri
Mesorhabditis_belari
Micoletzkya_japonica
Necator_americanus
Nippostrongylus_brasiliensis
Oscheius_tipulae
Parapristionchus_giblindavisi
Pristionchus_arcanus
Pristionchus_exspectatus
Pristionchus_fissidentatus
Pristionchus_japonicus
Pristionchus_maxplancki
Pristionchus_pacificus
Strongylus_vulgaris
Bursaphelenchus_xylophilus
Halicephalobus_mephisto
Panagrellus_redivivus
Steinernema_carpocapsae
Steinernema_feltiae
Steinernema_glaseri
Steinernema_monticolum
Steinernema_scapterisci
Acrobeloides_nanus
Ditylenchus_destructor
Ditylenchus_dipsaci

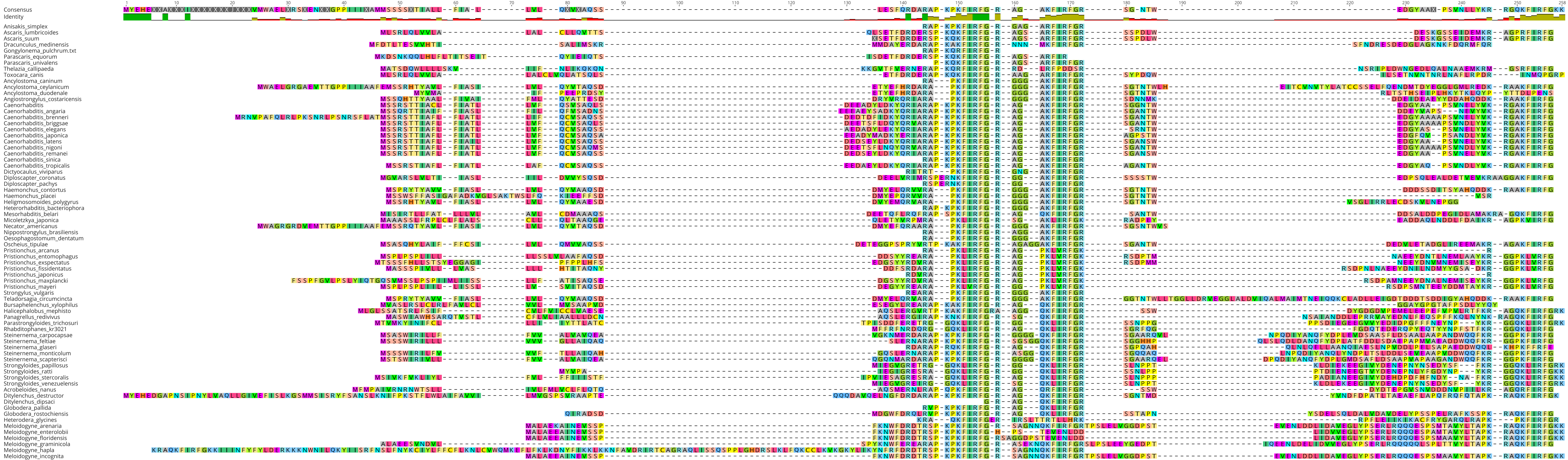
Consensus
Identity
Anisakis_simplex
Ascaris_lumbricoides
Ascaris_suum
Dracunculus_medinensis
Gongylonema_pulchrum.txt
Parascaris_equorum
Parascaris_univalens
Thelazia_callipaeda
Toxocara_canis
Ancylostoma_caninum
Ancylostoma_ceylanicum
Ancylostoma_duodenale
Angiostrongylus_costaricensis
Caenorhabditis
Caenorhabditis_angaria
Caenorhabditis_brenneri
Caenorhabditis_briggsae
Caenorhabditis_elegans
Caenorhabditis_japonica
Caenorhabditis_latens
Caenorhabditis_nigoni
Caenorhabditis_remanei
Caenorhabditis_sinica
Caenorhabditis_tropicalis
Dictyocaulus_viviparus
Diploscapter_coronatus
Diploscapter_pachys
Haemonchus_contortus
Haemonchus_placei
Heligmosomoides_polygyrus
Heterorhabditis_bacteriophora
Mesorhabditis_belari
Micoletzkya_japonica
Necator_americanus
Nippostrongylus_brasiliensis
Oesophagostomum_dentatum
Oscheius_tipulae
Pristionchus_arcanus
Pristionchus_entomophagus
Pristionchus_exspectatus
Pristionchus_fissidentatus
Pristionchus_japonicus
Pristionchus_maxplancki
Pristionchus_mayeri
Strongylus_vulgaris
Teladorsagia_circumcincta
Bursaphelenchus_xylophilus
Halicephalobus_mephisto
Panagrellus_redivivus
Parastrongyloides_trichosuri
Rhabditophanes_kr3021
Steinernema_carpocapsae
Steinernema_feltiae
Steinernema_glaseri
Steinernema_monticolum
Steinernema_scapterisci
Strongyloides_papillosus
Strongyloides_ratti
Strongyloides_stercoralis
Strongyloides_venezuelensis
Acrobeloides_nanus
Ditylenchus_destructor
Ditylenchus_dipsaci
Globodera_pallida
Globodera_rostochiensis
Heterodera_glycines
Meloidogyne_arenaria
Meloidogyne_enterolobii
Meloidogyne_floridensis
Meloidogyne_graminicola
Meloidogyne_hapla
Meloidogyne_incognita

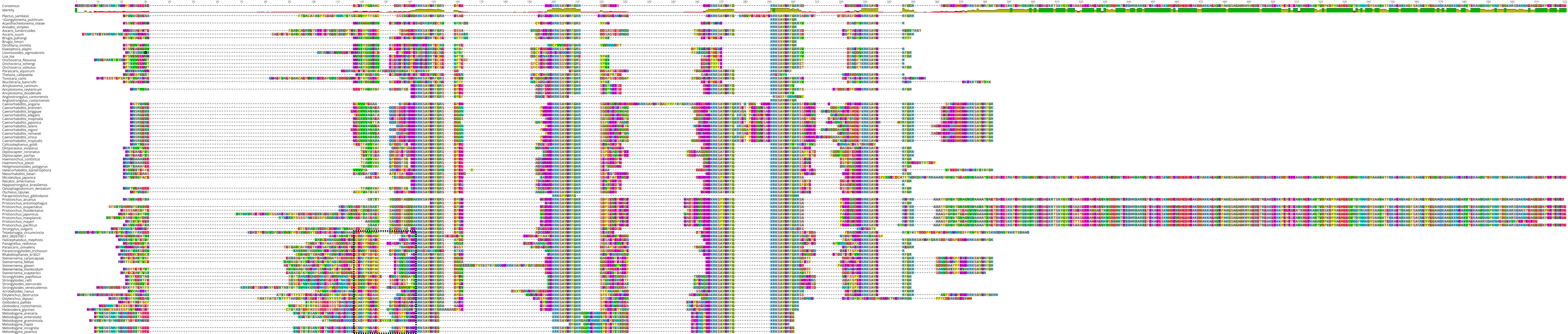
Consensus
Identity
Plectus_sambesii
<Gongylonema_pulchrum
Acanthocheilonema_viteae
Anisakis_simplex
Ascaris_lumbricoides
Ascaris_suum
Brugia_pahangi
Brugia_timori
Dirofilaria_immitis
Elaeophora_elaphi
Litomosoides_sigmodontis
Loa_loa
Onchocerca_flexuosa
Onchocerca_ochengi
Onchocerca_volvulus
Parascaris_equorum
Thelazia_callipaeda
Toxocara_canis
Wuchereria_bancrofti
Ancylostoma_caninum
Ancylostoma_ceylanicum
Ancylostoma_duodenale
Angiostrongylus_cantonensis
Angiostrongylus_costaricensis
Caenorhabditis_angaria
Caenorhabditis_brenneri
Caenorhabditis_briggsae
Caenorhabditis_elegans
Caenorhabditis_inopinata
Caenorhabditis_japonica
Caenorhabditis_latens
Caenorhabditis_nigoni
Caenorhabditis_remanei
Caenorhabditis_sinica
Caenorhabditis_tropicalis
Cylicostephanus_goldi
Dictyocaulus_viviparus
Diploscapter_coronatus
Diploscapter_pachys
Haemonchus_contortus
Haemonchus_placei
Heligmosomoides_polygyrus
Heterorhabditis_bacteriophora
Mesorhabditis_belari
Micoletzkya_japonica
Necator_americanus
Nippostrongylus_brasiliensis
Oesophagostomum_dentatum
Oscheius_tipulae
Parapristionchus_giblindavisI
Pristionchus_arcanus
Pristionchus_entomophagus
Pristionchus_exspectatus
Pristionchus_fissidentatus
Pristionchus_japonicus
Pristionchus_maxplancki
Pristionchus_mayeri
Pristionchus_pacificus
Strongylus_vulgaris
Teladorsagia_circumcincta
BXY_0826700.1
Halicephalobus_mephisto
Panagrellus_redivivus
Parascaris_univalens
Parastrongyloides_trichosuri
Rhabditophanes_kr3021
Steinernema_carpocapsae
Steinernema_feltiae
Steinernema_glaseri
Steinernema_monticolum
Steinernema_scapterisci
Strongyloides_papillosus
Strongyloides_ratti
Strongyloides_stercoralis
Strongyloides_venezuelensis
Acrobeloides_nanus
Ditylenchus_destructor
Ditylenchus_dipsaci
Globodera_pallida
Globodera_rostochiensis
Heterodera_glycines
Meloidogyne_arenaria
Meloidogyne_enterolobii
Meloidogyne_graminicola
Meloidogyne_hapla
Meloidogyne_incognita
Meloidogyne_javanica

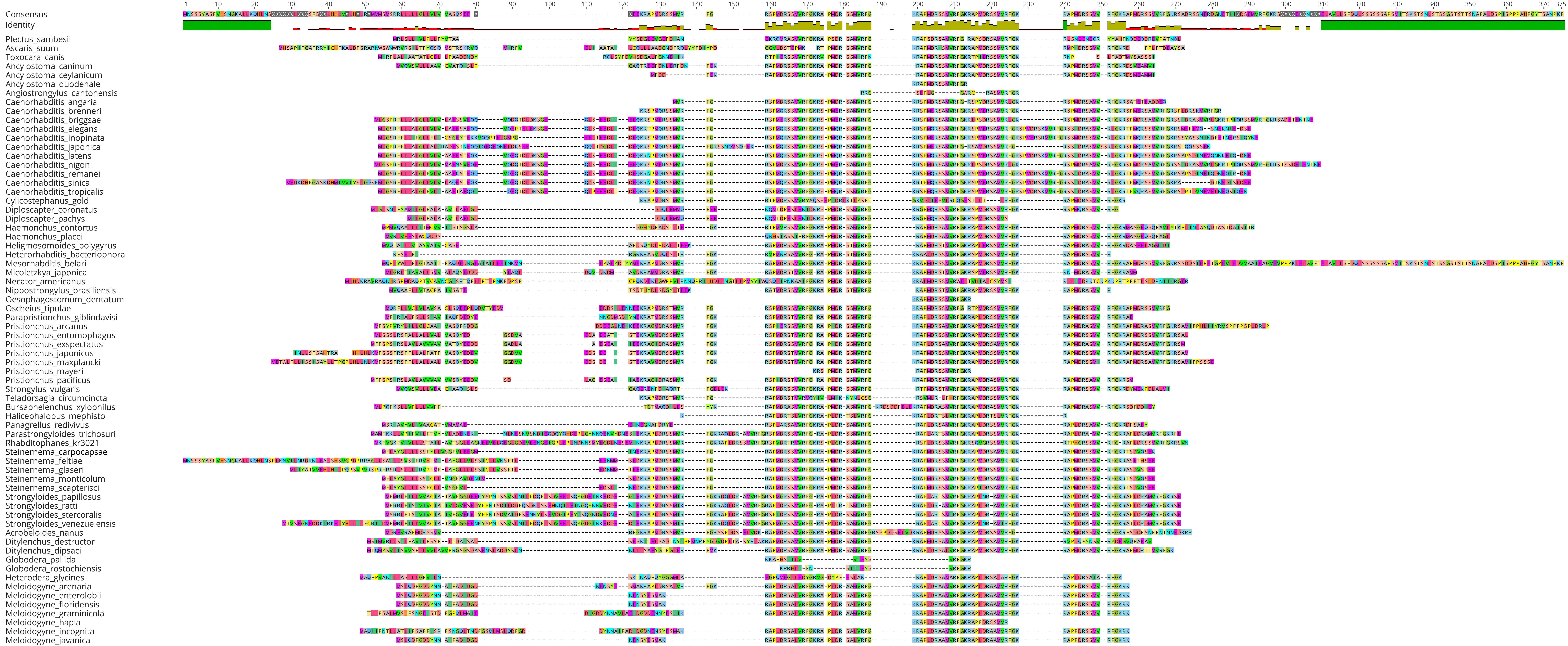
Consensus
Identity
Plectus_sambesii
Ascaris_suum
Toxocara_canis
Ancylostoma_caninum
Ancylostoma_ceylanicum
Ancylostoma_duodenale
Angiostrongylus_cantonensis
Caenorhabditis_angaria
Caenorhabditis_brenneri
Caenorhabditis_briggsae
Caenorhabditis_elegans
Caenorhabditis_inopinata
Caenorhabditis_japonica
Caenorhabditis_latens
Caenorhabditis_nigoni
Caenorhabditis_remanei
Caenorhabditis_sinica
Caenorhabditis_tropicalis
Cylicostephanus_goldi
Diploscapter_coronatus
Diploscapter_pachys
Haemonchus_contortus
Haemonchus_placei
Heligmosomoides_polygyrus
Heterorhabditis_bacteriophora
Mesorhabditis_belari
Micoletzkya_japonica
Necator_americanus
Nippostrongylus_brasiliensis
Oesophagostomum_dentatum
Oscheius_tipulae
Parapristionchus_giblindavisi
Pristionchus_arcanus
Pristionchus_entomophagus
Pristionchus_exspectatus
Pristionchus_japonicus
Pristionchus_maxplancki
Pristionchus_mayeri
Pristionchus_pacificus
Strongylus_vulgaris
Teladorsagia_circumcincta
Bursaphelenchus_xylophilus
Halicephalobus_mephisto
Panagrellus_redivivus
Parastrongyloides_trichosuri
Rhabditophanes_kr3021
Steinernema_carpocapsae
Steinernema_feltiae
Steinernema_glaseri
Steinernema_monticolum
Steinernema_scapterisci
Strongyloides_papillosus
Strongyloides_ratti
Strongyloides_stercoralis
Strongyloides_venezuelensis
Acrobeloides_nanus
Ditylenchus_destructor
Ditylenchus_dipsaci
Globodera_pallida
Globodera_rostochiensis
Heterodera_glycines
Meloidogyne_arenaria
Meloidogyne_enterolobii
Meloidogyne_floridensis
Meloidogyne_graminicola
Meloidogyne_hapla
Meloidogyne_incognita
Meloidogyne_javanica

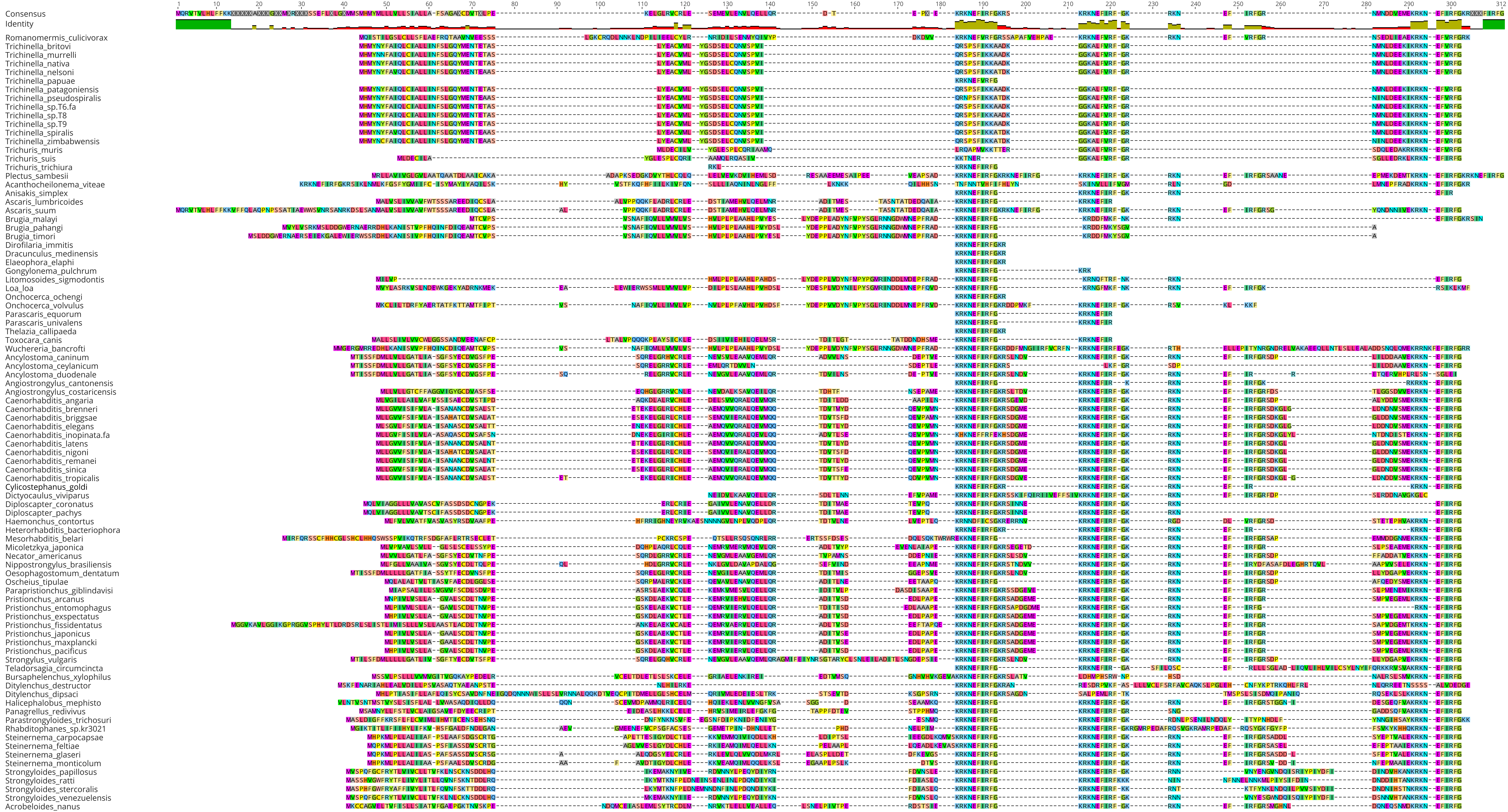

Consensus
Identity
Romanomermis_culicivorax
Trichinella_britovi
Trichinella_murrelli
Trichinella_nativa
Trichinella_nelsoni
Trichinella_papuae
Trichinella_patagoniensis
Trichinella_pseudospiralis
Trichinella_sp.T6.fa
Trichinella_sp.T8
Trichinella_sp.T9
Trichinella_spiralis
Trichinella_zimbabwensis
Trichuris_muris
Trichuris_suis
Trichuris_trichiura
Plectus_sambesii
Acanthocheilonema_viteae
Anisakis_simplex
Ascaris_lumbricoides
Ascaris_suum
Brugia_malayi
Brugia_pahangi
Brugia_timori
Dirofilaria_immitis
Dracunculus_medinensis
Elaeophora_elaphi
Gongylonema_pulchrum
Litomosoides_sigmodontis
Loa_loa
Onchocerca_ochengi
Onchocerca_volvulus
Parascaris_equorum
Parascaris_univalens
Thelazia_callipaeda
Toxocara_canis
Wuchereria_bancrofti
Ancylostoma_caninum
Ancylostoma_ceylanicum
Ancylostoma_duodenale
Angiostrongylus_cantonensis
Angiostrongylus_costaricensis
Caenorhabditis_angaria
Caenorhabditis_brenneri
Caenorhabditis_briggsae
Caenorhabditis_elegans
Caenorhabditis_inopinata.fa
Caenorhabditis_latens
Caenorhabditis_nigoni
Caenorhabditis_remanei
Caenorhabditis_sinica
Caenorhabditis_tropicalis
Cylicostephanus_goldi
Dictyocaulus_viviparus
Diploscapter_coronatus
Diploscapter_pachys
Haemonchus_contortus
Heterorhabditis_bacteriophora
Mesorhabditis_belari
Micoletzkya_japonica
Necator_americanus
Nippostrongylus_brasiliensis
Oesophagostomum_dentatum
Oscheius_tipulae
Parapristionchus_giblindavisi
Pristionchus_arcanus
Pristionchus_entomophagus
Pristionchus_exspectatus
Pristionchus_fissidentatus
Pristionchus_japonicus
Pristionchus_maxplancki
Pristionchus_pacificus
Strongylus_vulgaris
Teladorsagia_circumcincta
Bursaphelenchus_xylophilus
Ditylenchus_destructor
Ditylenchus_dipsaci
Halicephalobus_mephisto
Panagrellus_redivivus
Parastrongyloides_trichosuri
Rhabditophanes_sp.kr3021
Steinernema_carpocapsae
Steinernema_feltiae
Steinernema_glaseri
Steinernema_monticolum
Strongyloides_papillosus
Strongyloides_ratti
Strongyloides_stercoralis
Strongyloides_venezuelensis
Acrobeloides_nanus

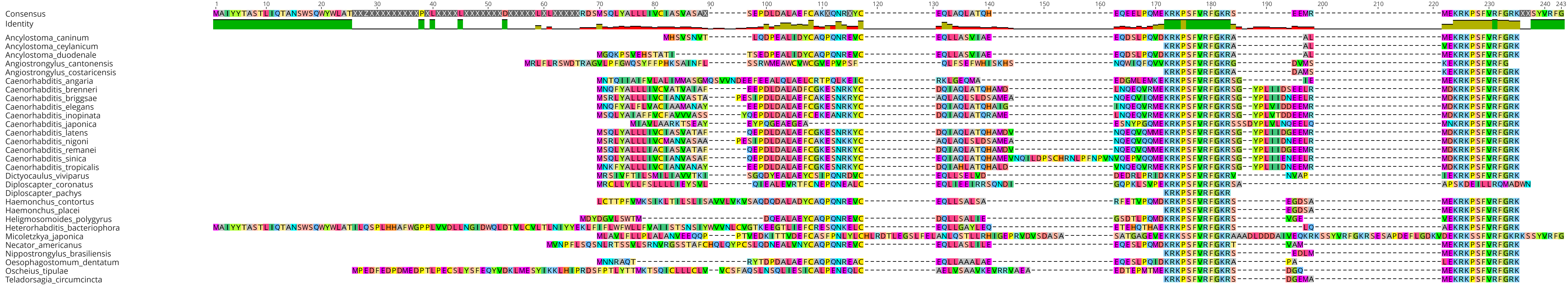
Consensus
Identity
1
10
20
30
40
50
60
70
80
90
100
110
120
130
140
150
160
170
180
190
200
210
220
230
240
243
Ancylostoma_caninum
Ancylostoma_ceylanicum
Ancylostoma_duodenale
Angiostrongylus_cantonensis
Angiostrongylus_costaricensis
Caenorhabditis_angaria
Caenorhabditis_brenneri
Caenorhabditis_briggsae
Caenorhabditis_elegans
Caenorhabditis_inopinata
Caenorhabditis_japonica
Caenorhabditis_latens
Caenorhabditis_nigoni
Caenorhabditis_remanei
Caenorhabditis_sinica
Caenorhabditis_tropicalis
Dictyocaulus_viviparus
Diploscapter_coronatus
Diploscapter_pachys
Haemonchus_contortus
Haemonchus_placei
Heligmosomoides_polygyrus
Heterorhabditis_bacteriophora
Micoletzkya_japonica
Necator_americanus
Nippostrongylus_brasiliensis
Oesophagostomum_dentatum
Oscheius_tipulae
Teladorsagia_circumcincta

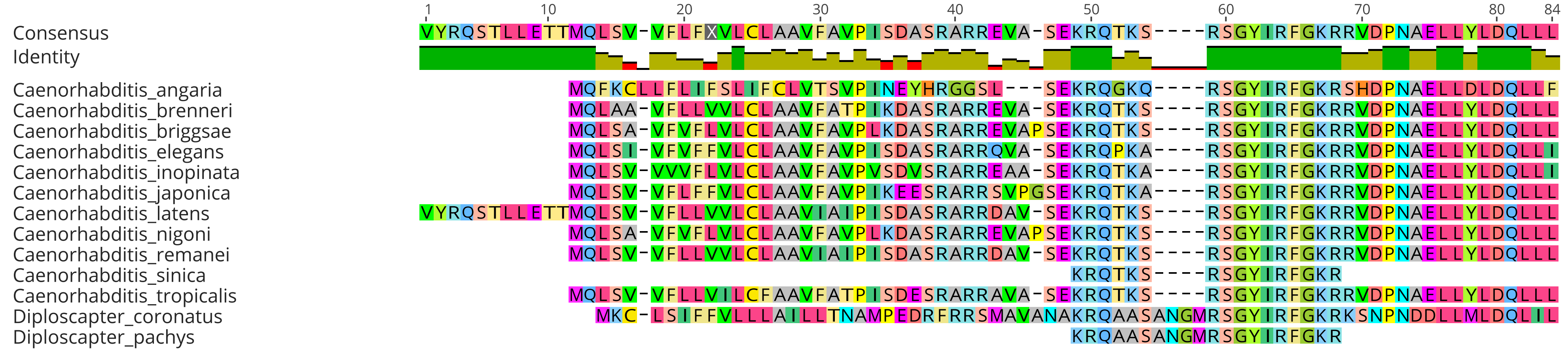
1
10
20
30
40
50
60
70
80
84
Consensus
Identity
Caenorhabditis_angaria
Caenorhabditis_brenneri
Caenorhabditis_briggsae
Caenorhabditis_elegans
Caenorhabditis_inopinata
Caenorhabditis_japonica
Caenorhabditis_latens
Caenorhabditis_nigoni
Caenorhabditis_remanei
Caenorhabditis_sinica
Caenorhabditis_tropicalis
Diploscapter_coronatus
Diploscapter_pachys

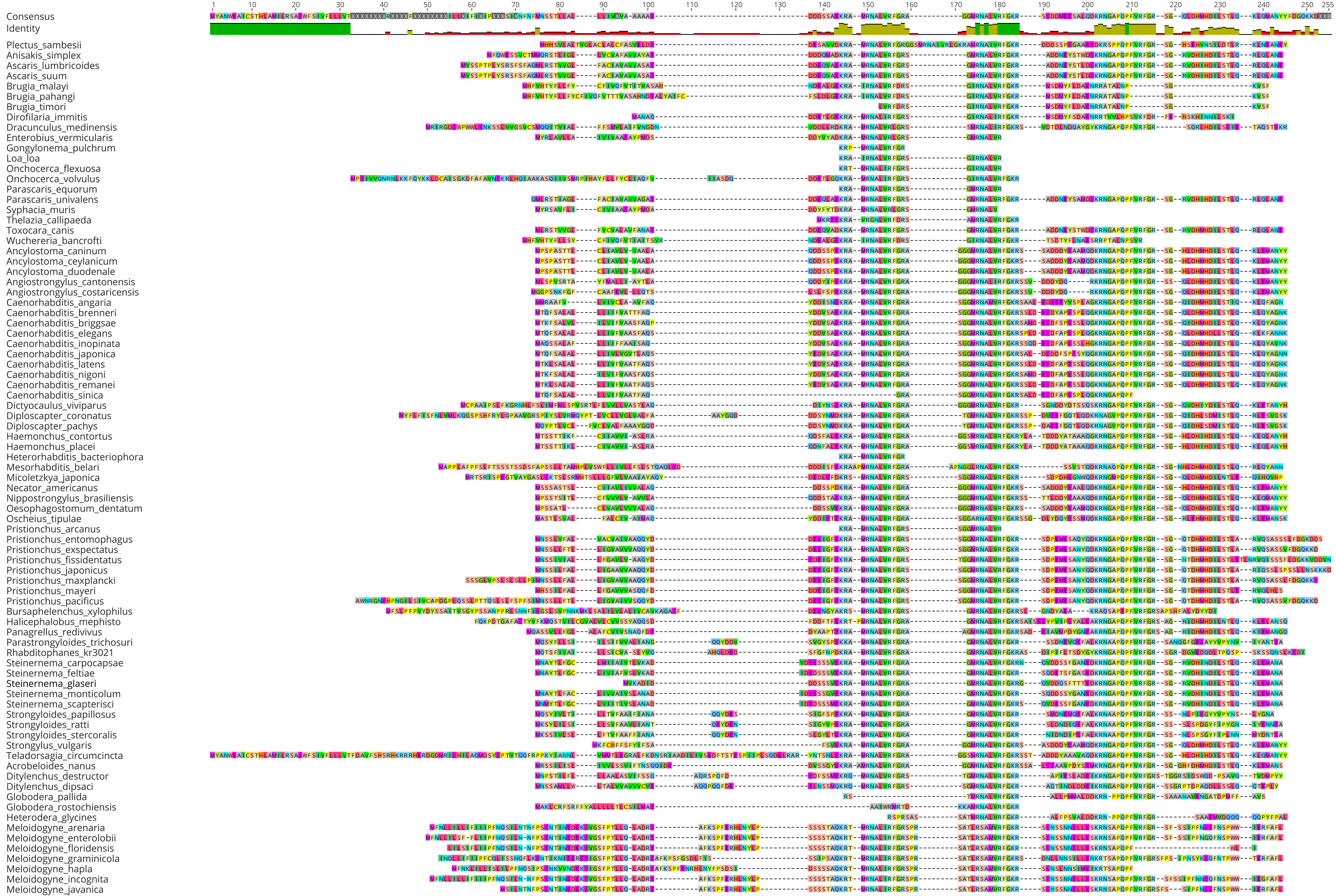
Consensus
Identity
Plectus_sambesii
Anisakis_simplex
Ascaris_lumbricoides
Ascaris_suum
Brugia_malayi
Brugia_pahangi
Brugia_timori
Dirofilaria_immitis
Dracunculus_medinensis
Enterobius_vermicularis
Gongylonema_pulchrum
Loa_loa
Onchocerca_flexuosa
Onchocerca_volvulus
Parascaris_equorum
Parascaris_univalens
Syphacia_muris
Thelazia_callipaeda
Toxocara_canis
Wuchereria_bancrofti
Ancylostoma_caninum
Ancylostoma_ceylanicum
Ancylostoma_duodenale
Angiostrongylus_cantonensis
Angiostrongylus_costaricensis
Caenorhabditis_angaria
Caenorhabditis_brenneri
Caenorhabditis_briggsae
Caenorhabditis_elegans
Caenorhabditis_inopinata
Caenorhabditis_japonica
Caenorhabditis_latens
Caenorhabditis_nigoni
Caenorhabditis_remanei
Caenorhabditis_sinica
Dictyocaulus_viviparus
Diploscapter_coronatus
Diploscapter_pachys
Haemonchus_contortus
Haemonchus_placei
Heterorhabditis_bacteriophora
Mesorhabditis_belari
Micoletzkya_japonica
Necator_americanus
Nippostrongylus_brasiliensis
Oesophagostomum_dentatum
Oscheius_tipulae
Pristionchus_arcanus
Pristionchus_entomophagus
Pristionchus_exspectatus
Pristionchus_fissidentatus
Pristionchus_japonicus
Pristionchus_maxplancki
Pristionchus_mayeri
Pristionchus_pacificus
Bursaphelenchus_xylophilus
Halicephalobus_mephisto
Panagrellus_redivivus
Parastrongyloides_trichosuri
Rhabditophanes_kr3021
Steinernema_carpocapsae
Steinernema_feltiae
Steinernema_glaseri
Steinernema_monticolum
Steinernema_scapterisci
Strongyloides_papillosus
Strongyloides_ratti
Strongyloides_stercoralis
Strongylus_vulgaris
Teladorsagia_circumcincta
Acrobeloides_nanus
Ditylenchus_destructor
Ditylenchus_dipsaci
Globodera_pallida
Globodera_rostochiensis
Heterodera_glycines
Meloidogyne_arenaria
Meloidogyne_enterolobii
Meloidogyne_floridensis
Meloidogyne_graminicola
Meloidogyne_hapla
Meloidogyne_incognita
Meloidogyne_javanica

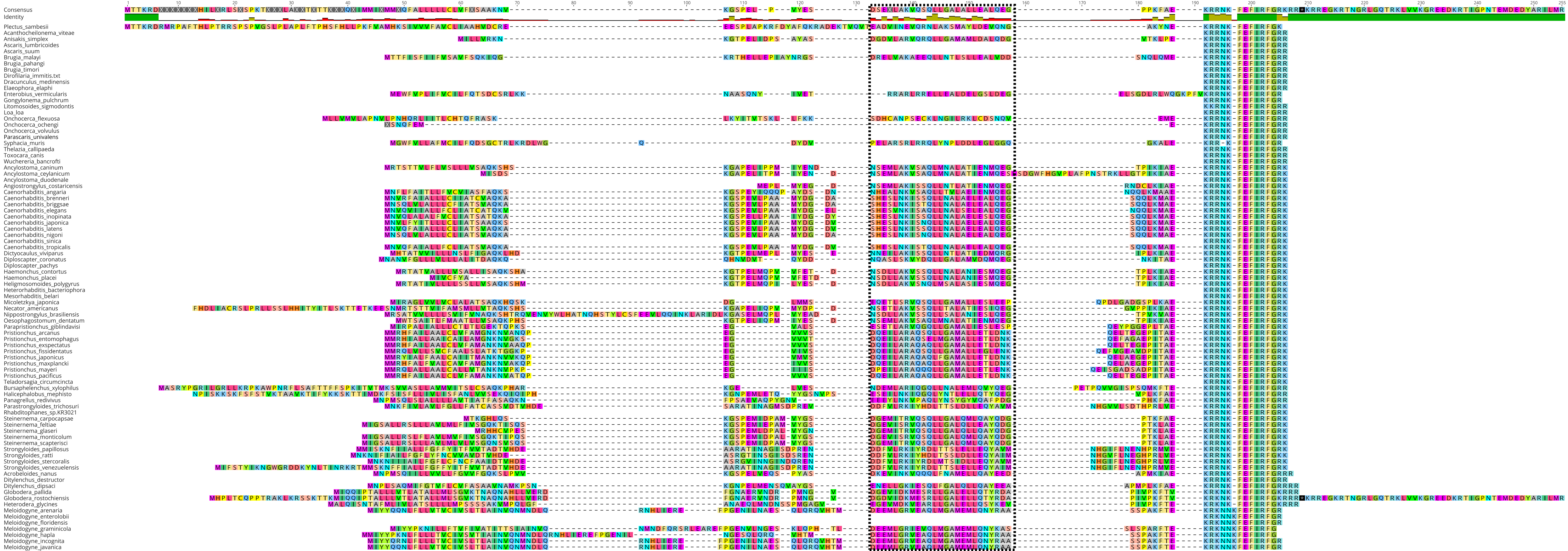
Consensus
Identity
Plectus_sambesii
Acanthocheilonema_viteae
Anisakis_simplex
Ascaris_lumbricoides
Ascaris_suum
Brugia_malayi
Brugia_pahangi
Brugia_timori
Dirofilaria_immitis.txt
Dracunculus_medinensis
Elaeophora_elaphi
Enterobius_vermicularis
Gongylonema_pulchrum
Litomosoides_sigmodontis
Loa_loa
Onchocerca_flexuosa
Onchocerca_ochengi
Onchocerca_volvulus
Parascaris_univalens
Syphacia_muris
Thelazia_callipaeda
Toxocara_canis
Wuchereria_bancrofti
Ancylostoma_caninum
Ancylostoma_ceylanicum
Ancylostoma_duodenale
Angiostrongylus_costaricensis
Caenorhabditis_angaria
Caenorhabditis_brenneri
Caenorhabditis_briggsae
Caenorhabditis_elegans
Caenorhabditis_inopinata
Caenorhabditis_japonica
Caenorhabditis_latens
Caenorhabditis_nigoni
Caenorhabditis_sinica
Caenorhabditis_tropicalis
Dictyocaulus_viviparus
Diploscapter_coronatus
Diploscapter_pachys
Haemonchus_contortus
Haemonchus_placei
Heligmosomoides_polygyrus
Heterorhabditis_bacteriophora
Mesorhabditis_belari
Micoletzkya_japonica
Necator_americanus
Nippostrongylus_brasiliensis
Oesophagostomum_dentatum
Parapristionchus_giblindavisi
Pristionchus_arcanus
Pristionchus_entomophagus
Pristionchus_exspectatus
Pristionchus_fissidentatus
Pristionchus_japonicus
Pristionchus_maxplancki
Pristionchus_mayeri
Pristionchus_pacificus
Teladorsagia_circumcincta
Bursaphelenchus_xylophilus
Halicephalobus_mephisto
Panagrellus_redivivus
Parastrongyloides_trichosuri
Rhabditophanes_sp.KR3021
Steinernema_carpocapsae
Steinernema_feltiae
Steinernema_glaseri
Steinernema_monticolum
Steinernema_scapterisci
Strongyloides_papillosus
Strongyloides_ratti
Strongyloides_stercoralis
Strongyloides_venezuelensis
Acrobeloides_nanus
Ditylenchus_destructor
Ditylenchus_dipsaci
Globodera_pallida
Globodera_rostochiensis
Heterodera_glycines
Meloidogyne_arenaria
Meloidogyne_enterolobii
Meloidogyne_floridensis
Meloidogyne_graminicola
Meloidogyne_hapla
Meloidogyne_incognita
Meloidogyne_javanica

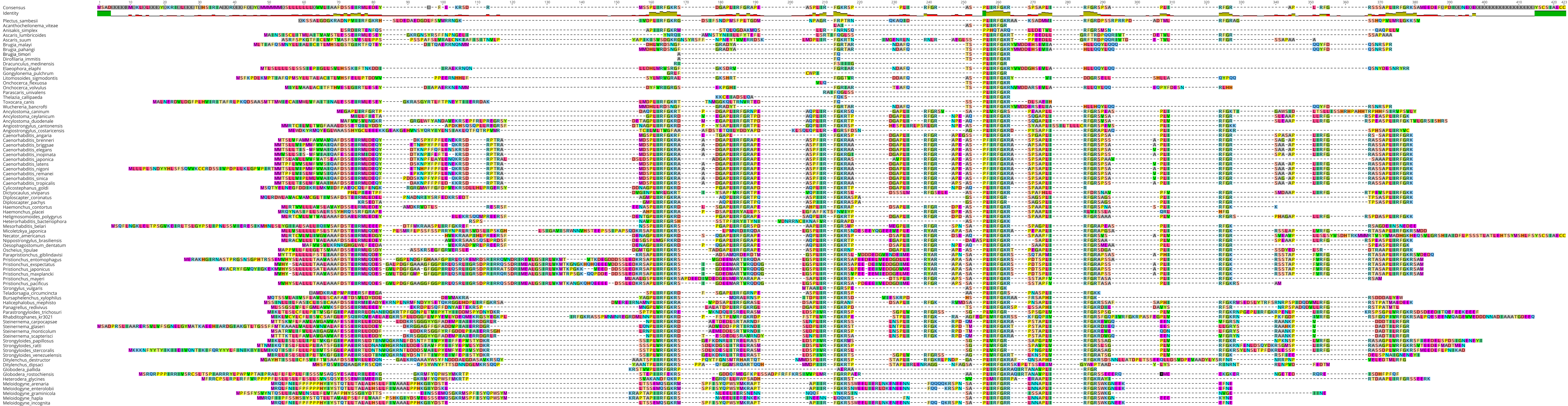
Consensus
Identity
Plectus_sambesii
Acanthocheilonema_viteae
Anisakis_simplex
Ascaris_lumbricoides
Ascaris_suum
Brugia_malayi
Brugia_pahangi
Brugia_timori
Dirofilaria_immitis
Dracunculus_medinensis
Elaeophora_elaphi
Gongylonema_pulchrum
Litomosoides_sigmodontis
Onchocerca_flexuosa
Onchocerca_volvulus
Parascaris_univalens
Thelazia_callipaeda
Toxocara_canis
Wuchereria_bancrofti
Ancylostoma_caninum
Ancylostoma_ceylanicum
Ancylostoma_duodenale
Angiostrongylus_cantonensis
Angiostrongylus_costaricensis
Caenorhabditis_angaria
Caenorhabditis_brenneri
Caenorhabditis_briggsae
Caenorhabditis_elegans
Caenorhabditis_inopinata
Caenorhabditis_japonica
Caenorhabditis_latens
Caenorhabditis_nigoni
Caenorhabditis_remanei
Caenorhabditis_sinica
Caenorhabditis_tropicalis
Cylicostephanus_goldi
Dictyocaulus_viviparus
Diploscapter_coronatus
Diploscapter_pachys
Haemonchus_contortus
Haemonchus_placei
Heligmosomoides_polygyrus
Heterorhabditis_bacteriophora
Mesorhabditis_belari
Micoletzkya_japonica
Necator_americanus
Nippostrongylus_brasiliensis
Oesophagostomum_dentatum
Oscheius_tipulae
Parapristionchus_giblindavisi
Pristionchus_entomophagus
Pristionchus_exspectatus
Pristionchus_japonicus
Pristionchus_maxplancki
Pristionchus_mayeri
Pristionchus_pacificus
Strongylus_vulgaris
Teladorsagia_circumcincta
Bursaphelenchus_xylophilus
Halicephalobus_mephisto
Panagrellus_redivivus
Parastrongyloides_trichosuri
Rhabditophanes_kr3021
Steinernema_carpocapsae
Steinernema_glaseri
Steinernema_monticolum
Steinernema_scapterisci
Strongyloides_papillosus
Strongyloides_ratti
Strongyloides_stercoralis
Strongyloides_venezuelensis
Ditylenchus_destructor
Ditylenchus_dipsaci
Globodera_pallida
Globodera_rostochiensis
Heterodera_glycines
Meloidogyne_arenaria
Meloidogyne_enterolobii
Meloidogyne_graminicola
Meloidogyne_hapla
Meloidogyne_incognita

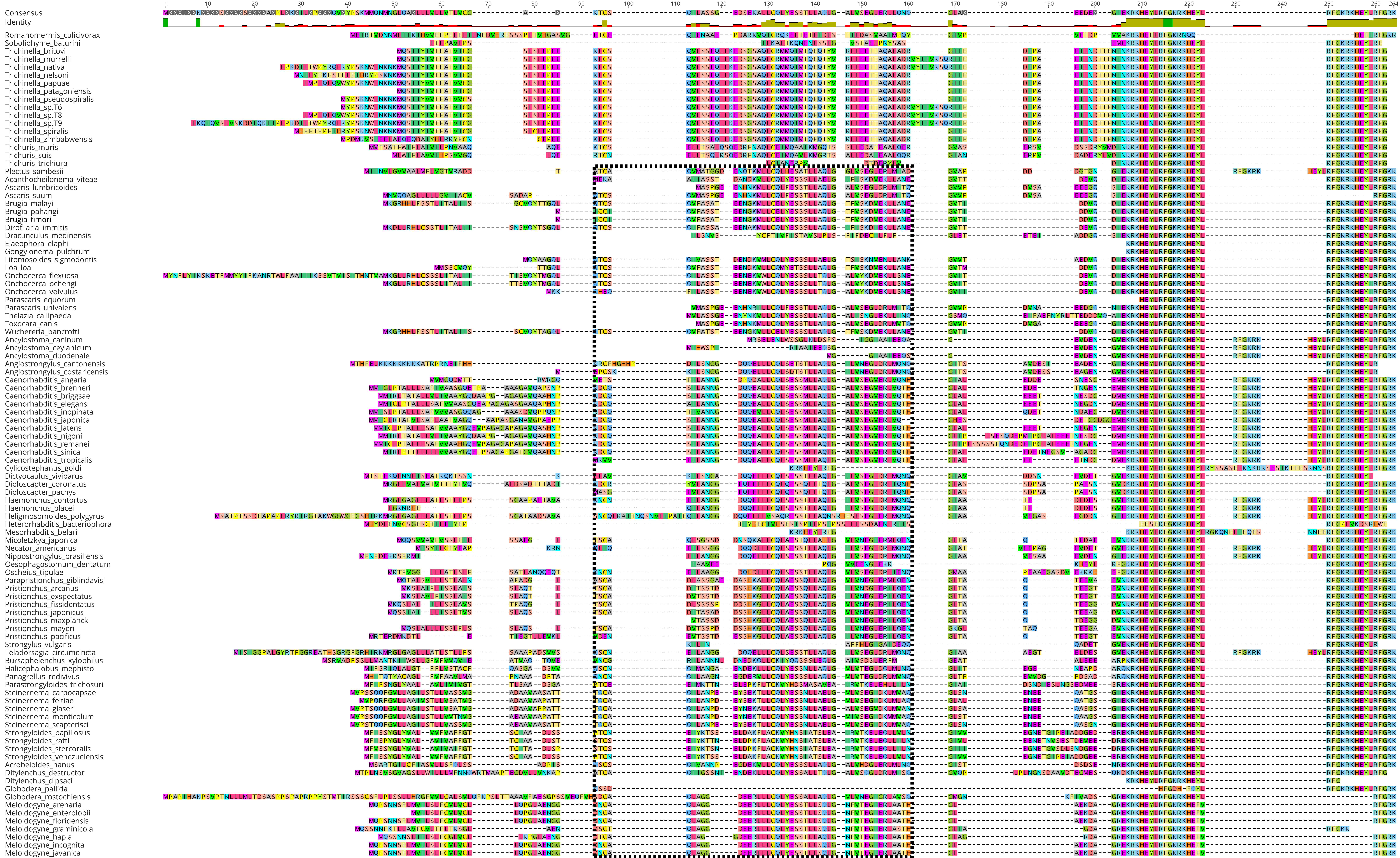
Consensus
Identity
1 10 20 30 40 50 60 70 80 90 100 110 120 130 140 150 160 170 180 190 200 210 220 230 240 250 260
Romanomermis_culicivorax
Soboliphyme_baturini
Trichinella_britovi
Trichinella_murrelli
Trichinella_nativa
Trichinella_nelsoni
Trichinella_papuae
Trichinella_patagoniensis
Trichinella_pseudospiralis
Trichinella_sp.T6
Trichinella_sp.T8
Trichinella_sp.T9
Trichinella_spiralis
Trichinella_zimbabwensis
Trichuris_muris
Trichuris_suis
Trichuris_trichiura
Plectus_sambesii
Acanthocheilonema_viteae
Ascaris_lumbricoides
Ascaris_suum
Brugia_malayi
Brugia_pahangi
Brugia_timori
Dirofilaria_immitis
Dracunculus_medinensis
Elaeophora_elaphi
Gongylonema_pulchrum
Litomosoides_sigmodontis
Loa_loa
Onchocerca_flexuosa
Onchocerca_ochengi
Onchocerca_volvulus
Parascaris_equorum
Parascaris_univalens
Thelazia_callipaeda
Toxocara_canis
Wuchereria_bancrofti
Ancylostoma_caninum
Ancylostoma_ceylanicum
Ancylostoma_duodenale
Angiostrongylus_cantonensis
Angiostrongylus_costaricensis
Caenorhabditis_angaria
Caenorhabditis_brenneri
Caenorhabditis_briggsae
Caenorhabditis_elegans
Caenorhabditis_inopinata
Caenorhabditis_japonica
Caenorhabditis_latens
Caenorhabditis_nigoni
Caenorhabditis_remanei
Caenorhabditis_sinica
Caenorhabditis_tropicalis
Cylicostephanus_goldi
Dictyocaulus_viviparus
Diploscapter_coronatus
Diploscapter_pachys
Haemonchus_contortus
Haemonchus_placei
Heligmosomoides_polygyrus
Heterorhabditis_bacteriophora
Mesorhabditis_belari
Micoletzkya_japonica
Necator_americanus
Nippostrongylus_brasiliensis
Oesophagostomum_dentatum
Oscheius_tipulae
Parapristionchus_giblindavisi
Pristionchus_arcanus
Pristionchus_exspectatus
Pristionchus_fissidentatus
Pristionchus_japonicus
Pristionchus_maxplancki
Pristionchus_mayeri
Pristionchus_pacificus
Strongylus_vulgaris
Teladorsagia_circumcincta
Bursaphelenchus_xylophilus
Halicephalobus_mephisto
Panagrellus_redivivus
Parastrongyloides_trichosuri
Steinernema_carpocapsae
Steinernema_feltiae
Steinernema_glaseri
Steinernema_monticolum
Steinernema_scapterisci
Strongyloides_papillosus
Strongyloides_ratti
Strongyloides_stercoralis
Strongyloides_venezuelensis
Acrobeloides_nanus
Ditylenchus_destructor
Ditylenchus_dipsaci
Globodera_pallida
Globodera_rostochiensis
Meloidogyne_arenaria
Meloidogyne_enterolobii
Meloidogyne_floridensis
Meloidogyne_graminicola
Meloidogyne_hapla
Meloidogyne_incognita
Meloidogyne_javanica

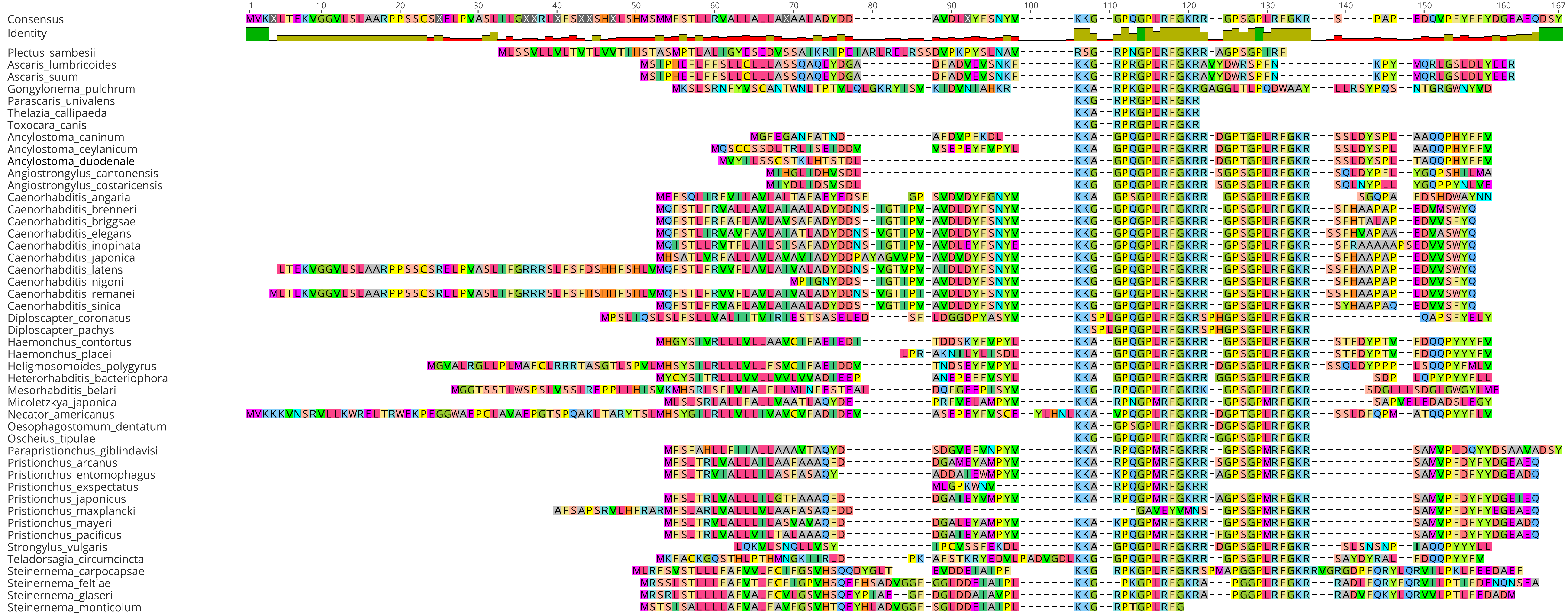

1
10
20
30
40
50
60
70
80
90
100
110
120
130
140
150
160
167
Consensus
Identity
Plectus_sambesii
Ascaris_lumbricoides
Ascaris_suum
Gongylonema_pulchrum
Parascaris_univalens
Thelazia_callipaeda
Toxocara_canis
Ancylostoma_caninum
Ancylostoma_ceylanicum
Ancylostoma_duodenale
Angiostrongylus_cantonensis
Angiostrongylus_costaricensis
Caenorhabditis_angaria
Caenorhabditis_brenneri
Caenorhabditis_briggsae
Caenorhabditis_elegans
Caenorhabditis_inopinata
Caenorhabditis_japonica
Caenorhabditis_latens
Caenorhabditis_nigoni
Caenorhabditis_remanei
Caenorhabditis_sinica
Diploscapter_coronatus
Diploscapter_pachys
Haemonchus_contortus
Haemonchus_placei
Heligmosomoides_polygyrus
Heterorhabditis_bacteriophora
Mesorhabditis_belari
Micoletzkya_japonica
Necator_americanus
Oesophagostomum_dentatum
Oscheius_tipulae
Parapristionchus_giblindavisi
Pristionchus_arcanus
Pristionchus_entomophagus
Pristionchus_exspectatus
Pristionchus_japonicus
Pristionchus_maxplancki
Pristionchus_mayeri
Pristionchus_pacificus
Strongylus_vulgaris
Teladorsagia_circumcincta
Steinernema_carpocapsae
Steinernema_feltiae
Steinernema_glaseri
Steinernema_monticolum

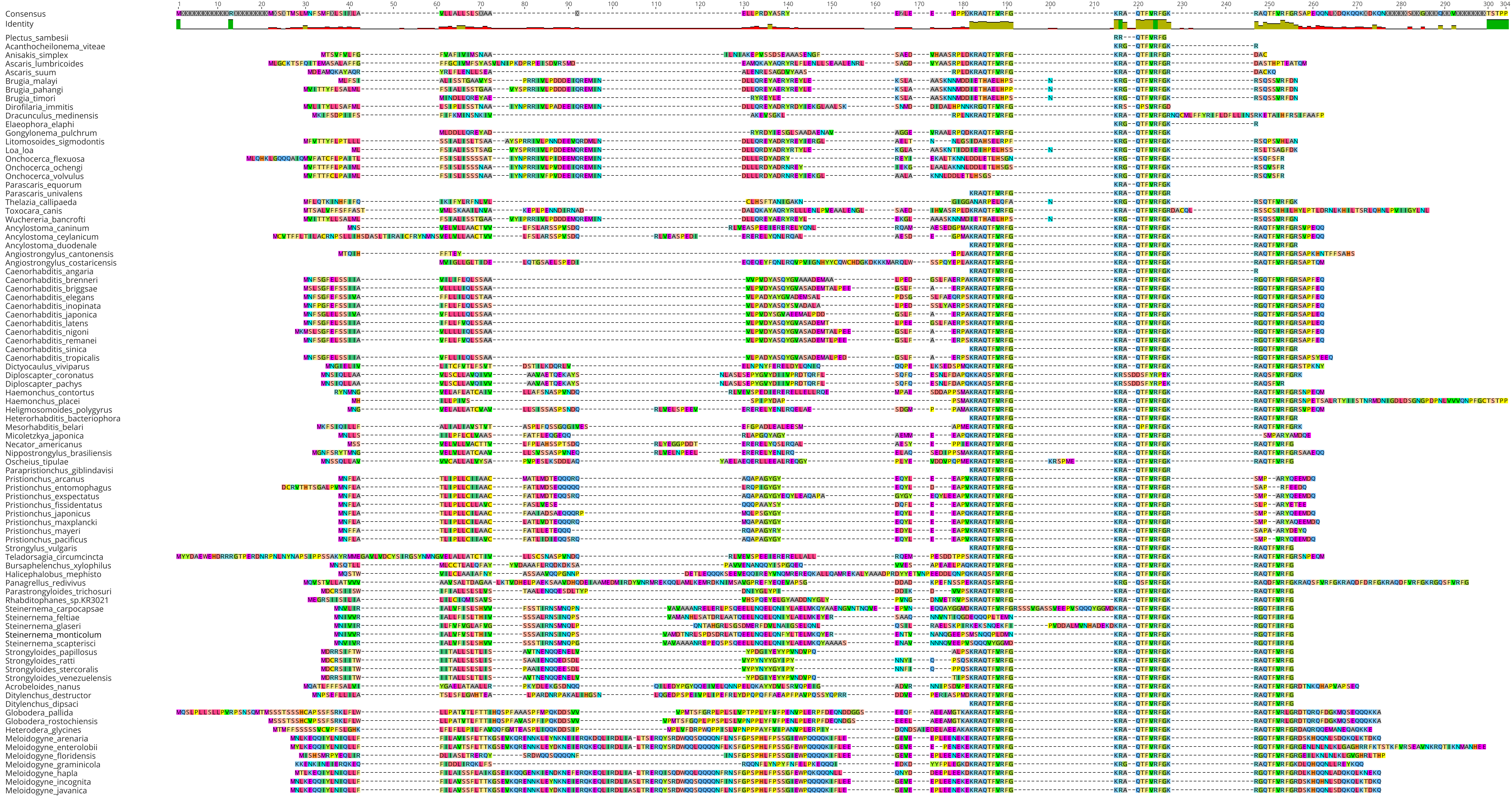
Consensus
Identity
Plectus_sambesii
Acanthocheilonema_viteae
Anisakis_simplex
Ascaris_lumbricoides
Ascaris_suum
Brugia_malayi
Brugia_pahangi
Brugia_timori
Dirofilaria_immitis
Dracunculus_medinensis
Elaeophora_elaphi
Gongylonema_pulchrum
Litomosoides_sigmodontis
Loa_loa
Onchocerca_flexuosa
Onchocerca_ochengi
Onchocerca_volvulus
Parascaris_equorum
Parascaris_univalens
Thelazia_callipaeda
Toxocara_canis
Wuchereria_bancrofti
Ancylostoma_caninum
Ancylostoma_ceylanicum
Ancylostoma_duodenale
Angiostrongylus_cantonensis
Angiostrongylus_costaricensis
Caenorhabditis_angaria
Caenorhabditis_brenneri
Caenorhabditis_briggsae
Caenorhabditis_elegans
Caenorhabditis_inopinata
Caenorhabditis_japonica
Caenorhabditis_latens
Caenorhabditis_nigoni
Caenorhabditis_remanei
Caenorhabditis_sinica
Caenorhabditis_tropicalis
Dictyocaulus_viviparus
Diploscapter_coronatus
Diploscapter_pachys
Haemonchus_contortus
Haemonchus_placei
Heligmosomoides_polygyrus
Heterorhabditis_bacteriophora
Mesorhabditis_belari
Micoletzkya_japonica
Necator_americanus
Nippostrongylus_brasiliensis
Oscheius_tipulae
Parapristionchus_giblindavisi
Pristionchus_arcanus
Pristionchus_entomophagus
Pristionchus_exspectatus
Pristionchus_fissidentatus
Pristionchus_japonicus
Pristionchus_maxplancki
Pristionchus_mayeri
Pristionchus_pacificus
Strongylus_vulgaris
Teladorsagia_circumcincta
Bursaphelenchus_xylophilus
Halicephalobus_mephisto
Panagrellus_redivivus
Parastrongyloides_trichosuri
Rhabditophanes_sp.KR3021
Steinernema_carpocapsae
Steinernema_feltiae
Steinernema_glaseri
Steinernema_monticolum
Steinernema_scapterisci
Strongyloides_papillosus
Strongyloides_ratti
Strongyloides_stercoralis
Strongyloides_venezuelensis
Acrobeloides_nanus
Ditylenchus_destructor
Ditylenchus_dipsaci
Globodera_pallida
Globodera_rostochiensis
Heterodera_glycines
Meloidogyne_arenaria
Meloidogyne_enterolobii
Meloidogyne_floridensis
Meloidogyne_graminicola
Meloidogyne_hapla
Meloidogyne_incognita
Meloidogyne_javanica

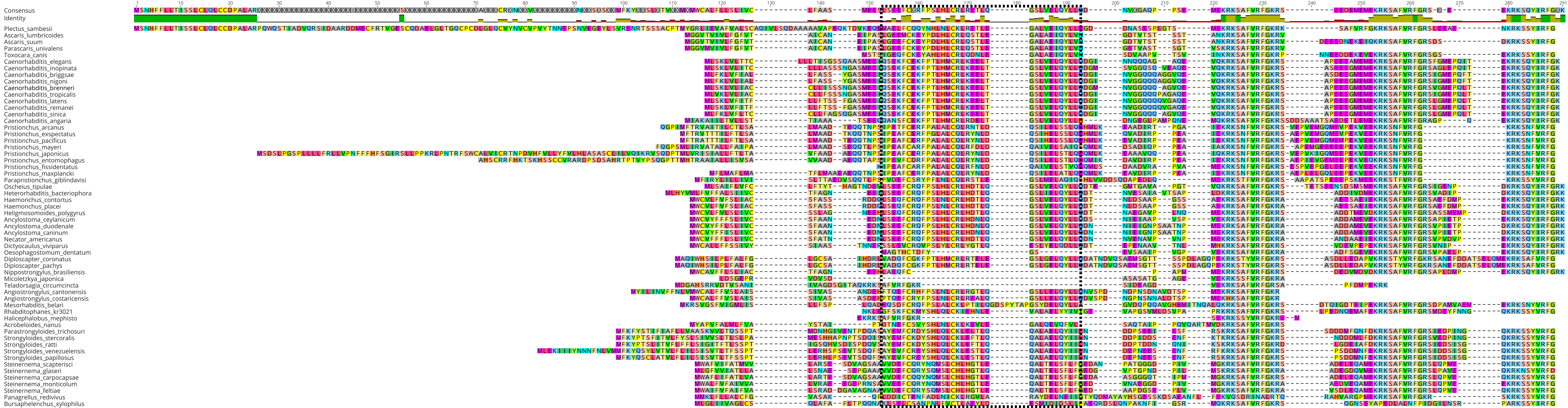
Consensus
Identity
Plectus_sambesii
Ascaris_lumbricoides
Ascaris_suum
Parascaris_univalens
Toxocara_canis
Caenorhabditis_elegans
Caenorhabditis_inopinata
Caenorhabditis_briggsae
Caenorhabditis_nigoni
Caenorhabditis_brenneri
Caenorhabditis_tropicalis
Caenorhabditis_latens
Caenorhabditis_remanei
Caenorhabditis_sinica
Caenorhabditis_angaria
Pristionchus_arcanus
Pristionchus_exspectatus
Pristionchus_pacificus
Pristionchus_mayeri
Pristionchus_japonicus
Pristionchus_entomophagus
Pristionchus_fissidentatus
Pristionchus_maxplancki
Parapristionchus_giblindavisi
Oscheius_tipulae
Heterorhabditis_bacteriophora
Haemonchus_contortus
Haemonchus_placei
Heligmosomoides_polygyrus
Ancylostoma_ceylanicum
Ancylostoma_duodenale
Ancylostoma_caninum
Necator_americanus
Dictyocaulus_viviparus
Oesophagostomum_dentatum
Diploscapter_coronatus
Diploscapter_pachys
Nippostrongylus_brasiliensis
Micoletzkya_japonica
Teladorsagia_circumcincta
Angiostrongylus_cantonensis
Angiostrongylus_costaricensis
Mesorhabditis_belari
Rhabditophanes_kr3021
Halicephalobus_mephisto
Acrobeloides_nanus
Parastrongyloides_trichosuri
Strongyloides_stercoralis
Strongyloides_ratti
Strongyloides_venezuelensis
Strongyloides_papillosus
Steinernema_scapterisci
Steinernema_glaseri
Steinernema_carpocapsae
Steinernema_monticolum
Steinernema_feltiae
Panagrellus_redivivus
Bursaphelenchus_xylophilus

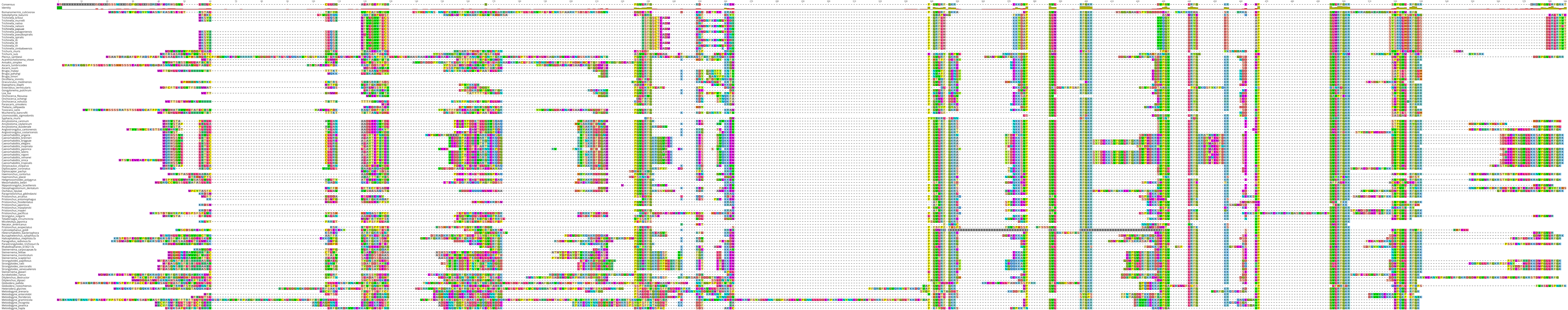
Consensus
Identity
Romanomermis_culicivorax
Soboliphyme_baturini
Trichinella_britovi
Trichinella_murrelli
Trichinella_nativa
Trichinella_nelsoni
Trichinella_papuae
Trichinella_patagoniensis
Trichinella_pseudospiralis
Trichinella_spiralis
Trichinella_t6
Trichinella_t8
Trichinella_t9
Trichinella_zimbabwensis
Trichuris_muris
Trichuris_suis
Plectus_sambesii
Acanthocheilonema_viteae
Anisakis_simplex
Ascaris_lumbricoides
Ascaris_suum
Brugia_malayi
Brugia_pahangi
Brugia_timori
Dirofilaria_immitis
Dracunculus_medinensis
Elaeophora_elaphi
Enterobius_vermicularis
Gongylonema_pulchrum
Loa_loa
Onchocerca_flexuosa
Onchocerca_ochengi
Onchocerca_volvulus
Parascaris_univalens
Thelazia_callipaeda
Toxocara_canis
Wuchereria_bancrofti
Litomosoides_sigmodontis
Syphacia_muris
Ancylostoma_caninum
Ancylostoma_ceylanicum
Ancylostoma_duodenale
Angiostrongylus_cantonensis
Angiostrongylus_costaricensis
Caenorhabditis_angaria
Caenorhabditis_brenneri
Caenorhabditis_briggsae
Caenorhabditis_elegans
Caenorhabditis_inopinata
Caenorhabditis_japonica
Caenorhabditis_latens
Caenorhabditis_nigoni
Caenorhabditis_remanei
Caenorhabditis_sinica
Caenorhabditis_tropicalis
Dictyocaulus_viviparus
Diploscapter_coronatus
Diploscapter_pachys
Haemonchus_contortus
Haemonchus_placei
Heligmosomoides_polygyrus
Mesorhabditis_belari
Nippostrongylus_brasiliensis
Oesophagostomum_dentatum
Oscheius_tipulae
Parapristionchus_giblindavisi
Pristionchus_arcanus
Pristionchus_entomophagus
Pristionchus_fissidentatus
Pristionchus_japonicus
Pristionchus_maxplancki
Pristionchus_mayeri
Pristionchus_pacificus
Strongylus_vulgaris
Teladorsagia_circumcincta
Micoletzkya_japonica
Necator_americanus
Pristionchus_exspectatus
Cylicostephanus_goldi
Heterorhabditis_bacteriophora
Bursaphelenchus_xylophilus.fa
Halicephalobus_mephisto.fa
Panagrellus_redivivus.fa
Parastrongyloides_trichosuri.fa
Rhabditophanes_kr3021.fa
Steinernema_carpocapsae.fa
Steinernema_feltiae
Steinernema_monticolum
Steinernema_scapterisci
Strongyloides_papillosus
Strongyloides_ratti
Strongyloides_stercoralis
Strongyloides_venezuelensis
Steinernema_glaseri
Acrobeloides_nanus
Ditylenchus_destructor
Ditylenchus_dipsaci
Globodera_pallida
Globodera_rostochiensis
Heterodera_glycines
Meloidogyne_arenaria
Meloidogyne_enterolobii
Meloidogyne_floridensis
Meloidogyne_graminicola
Meloidogyne_incognita
Meloidogyne_javanica
Meloidogyne_hapla

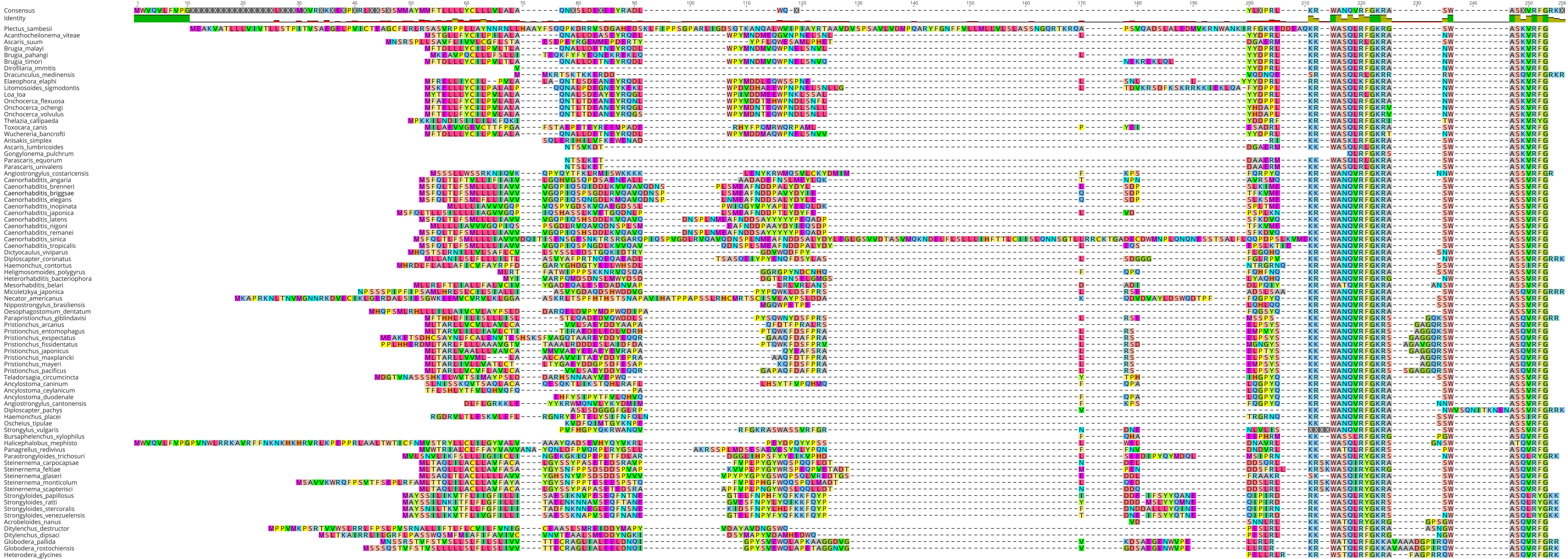

Consensus
Identity
1 10 20 30 40 50 60 70 80 90 100 110 120 130 140 150 160 170 180 190 200 210 220 230 240 250
Plectus_sambesii
Acanthocheilonema_viteae
Ascaris_suum
Brugia_malayi
Brugia_pahangi
Brugia_timori
Dirofilaria_immitis
Dracunculus_medinensis
Elaeophora_elaphi
Litomosoides_sigmodontis
Loa_loa
Onchocerca_flexuosa
Onchocerca_ochengi
Onchocerca_volvulus
Thelazia_callipaeda
Toxocara_canis
Wuchereria_bancrofti
Anisakis_simplex
Ascaris_lumbricoides
Gongylonema_pulchrum
Parascaris_equorum
Parascaris_univalens
Angiostrongylus_costaricensis
Caenorhabditis_angaria
Caenorhabditis_brenneri
Caenorhabditis_briggsae
Caenorhabditis_elegans
Caenorhabditis_inopinata
Caenorhabditis_japonica
Caenorhabditis_latens
Caenorhabditis_nigoni
Caenorhabditis_remanei
Caenorhabditis_sinica
Caenorhabditis_tropicalis
Dictyocaulus_viviparus
Diploscapter_coronatus
Haemonchus_contortus
Heligmosomoides_polygyrus
Heterorhabditis_bacteriophora
Mesorhabditis_belari
Micoletzkya_japonica
Necator_americanus
Nippostrongylus_brasiliensis
Oesophagostomum_dentatum
Parapristionchus_giblindavisi
Pristionchus_arcanus
Pristionchus_entomophagus
Pristionchus_exspectatus
Pristionchus_fissidentatus
Pristionchus_japonicus
Pristionchus_maxplancki
Pristionchus_mayeri
Pristionchus_pacificus
Teladorsagia_circumcincta
Ancylostoma_caninum
Ancylostoma_ceylanicum
Ancylostoma_duodenale
Angiostrongylus_cantonensis
Diploscapter_pachys
Haemonchus_placei
Oscheius_tipulae
Strongylus_vulgaris
Bursaphelenchus_xylophilus
Halicephalobus_mephisto
Panagrellus_redivivus
Parastrongyloides_trichosuri
Steinernema_carpocapsae
Steinernema_feltiae
Steinernema_glaseri
Steinernema_monticolum
Steinernema_scapterisci
Strongyloides_papillosus
Strongyloides_ratti
Strongyloides_stercoralis
Strongyloides_venezuelensis
Acrobeloides_nanus
Ditylenchus_destructor
Ditylenchus_dipsaci
Globodera_pallida
Globodera_rostochiensis
Heterodera_glycines

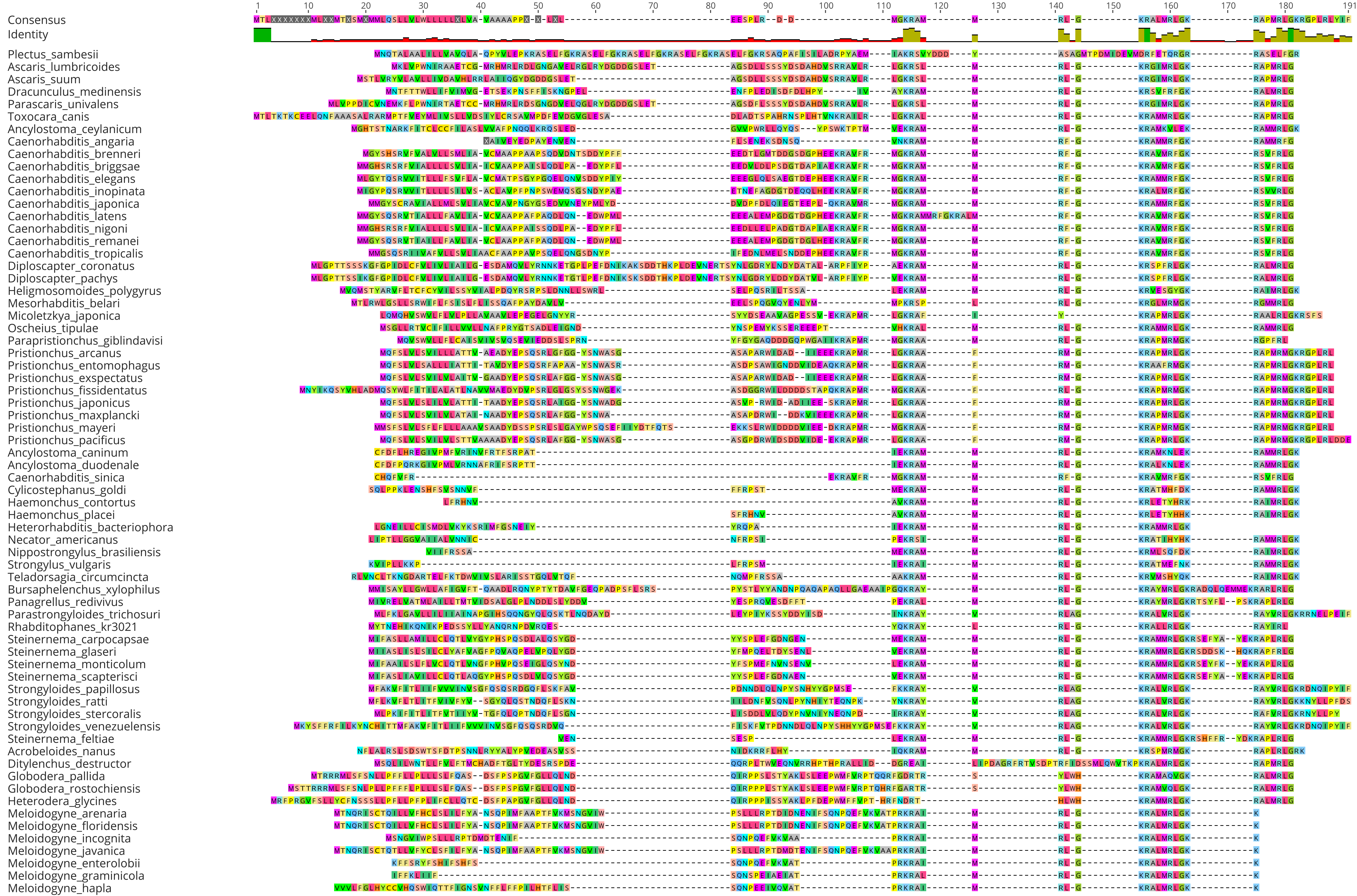

1 10 20 30 40 50 60 70 80 90 100 110 120 130 140 150 160 170 180 191
Consensus
Identity
Plectus_sambesii
Ascaris_lumbricoides
Ascaris_suum
Dracunculus_medinensis
Parascaris_univalens
Toxocara_canis
Ancylostoma_ceylanicum
Caenorhabditis_angaria
Caenorhabditis_brenneri
Caenorhabditis_briggsae
Caenorhabditis_elegans
Caenorhabditis_inopinata
Caenorhabditis_japonica
Caenorhabditis_latens
Caenorhabditis_nigoni
Caenorhabditis_remanei
Caenorhabditis_tropicalis
Diploscapter_coronatus
Diploscapter_pachys
Heligmosomoides_polygyrus
Mesorhabditis_belari
Micoletzkya_japonica
Oscheius_tipulae
Parapristionchus_giblindavisi
Pristionchus_arcanus
Pristionchus_entomophagus
Pristionchus_exspectatus
Pristionchus_fissidentatus
Pristionchus_japonicus
Pristionchus_maxplancki
Pristionchus_mayeri
Pristionchus_pacificus
Ancylostoma_caninum
Ancylostoma_duodenale
Caenorhabditis_sinica
Cylicostephanus_goldi
Haemonchus_contortus
Haemonchus_placei
Heterorhabditis_bacteriophora
Necator_americanus
Nippostrongylus_brasiliensis
Strongylus_vulgaris
Teladorsagia_circumcincta
Bursaphelenchus_xylophilus
Panagrellus_redivivus
Parastrongyloides_trichosuri
Rhabditophanes_kr3021
Steinernema_carpocapsae
Steinernema_glaseri
Steinernema_monticolum
Steinernema_scapterisci
Strongyloides_papillosus
Strongyloides_ratti
Strongyloides_stercoralis
Strongyloides_venezuelensis
Steinernema_feltiae
Acrobeloides_nanus
Ditylenchus_destructor
Globodera_pallida
Globodera_rostochiensis
Heterodera_glycines
Meloidogyne_arenaria
Meloidogyne_floridensis
Meloidogyne_incognita
Meloidogyne_javanica
Meloidogyne_enterolobii
Meloidogyne_graminicola
Meloidogyne_hapla

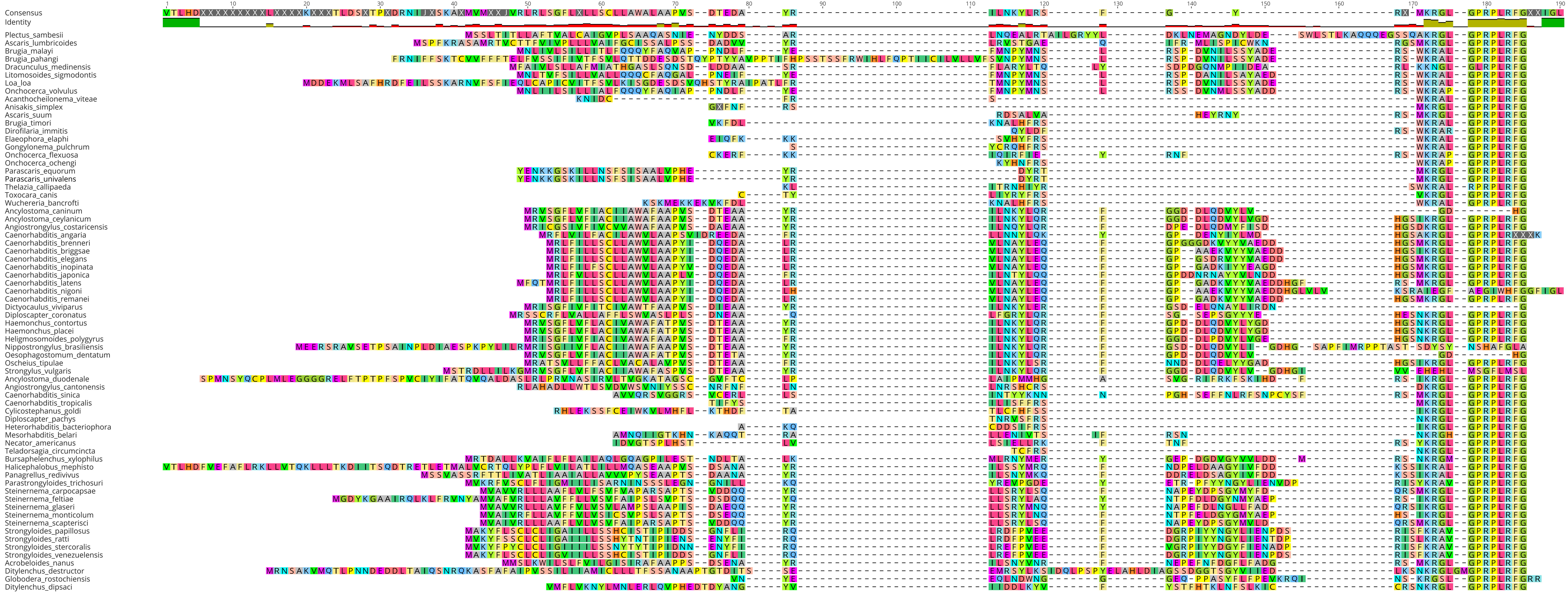

Consensus
Identity
Plectus_sambesii
Ascaris_lumbricoides
Brugia_malayi
Brugia_pahangi
Dracunculus_medinensis
Litomosoides_sigmodontis
Loa_loa
Onchocerca_volvulus
Acanthocheilonema_viteae
Anisakis_simplex
Ascaris_suum
Brugia_timori
Dirofilaria_immitis
Elaeophora_elaphi
Gongylonema_pulchrum
Onchocerca_flexuosa
Onchocerca_ochengi
Parascaris_equorum
Parascaris_univalens
Thelazia_callipaeda
Toxocara_canis
Wuchereria_bancrofti
Ancylostoma_caninum
Ancylostoma_ceylanicum
Angiostrongylus_costaricensis
Caenorhabditis_angaria
Caenorhabditis_brenneri
Caenorhabditis_briggsae
Caenorhabditis_elegans
Caenorhabditis_inopinata
Caenorhabditis_japonica
Caenorhabditis_latens
Caenorhabditis_nigoni
Caenorhabditis_remanei
Dictyocaulus_viviparus
Diploscapter_coronatus
Haemonchus_contortus
Haemonchus_placei
Heligmosomoides_polygyrus
Nippostrongylus_brasiliensis
Oesophagostomum_dentatum
Oscheius_tipulae
Strongylus_vulgaris
Ancylostoma_duodenale
Angiostrongylus_cantonensis
Caenorhabditis_sinica
Caenorhabditis_tropicalis
Cylicostephanus_goldi
Diploscapter_pachys
Heterorhabditis_bacteriophora
Mesorhabditis_belari
Necator_americanus
Teladorsagia_circumcincta
Bursaphelenchus_xylophilus
Halicephalobus_mephisto
Panagrellus_redivivus
Parastrongyloides_trichosuri
Steinernema_carpocapsae
Steinernema_feltiae
Steinernema_glaseri
Steinernema_monticolum
Steinernema_scapterisci
Strongyloides_papillosus
Strongyloides_ratti
Strongyloides_stercoralis
Strongyloides_venezuelensis
Acrobeloides_nanus
Ditylenchus_destructor
Globodera_rostochiensis
Ditylenchus_dipsaci

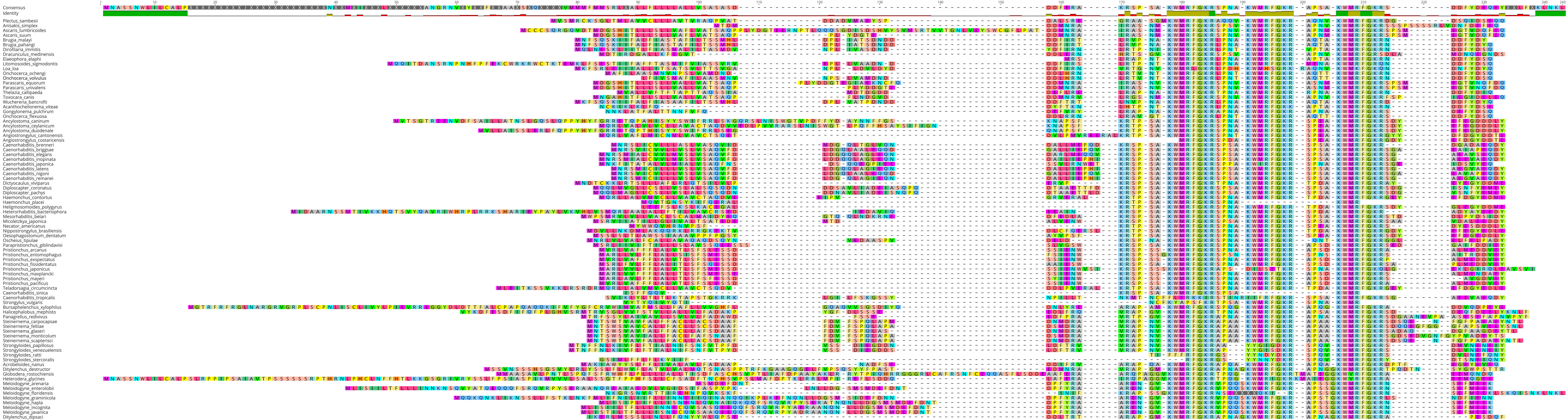

Consensus
Identity
Plectus_sambesii
Anisakis_simplex
Ascaris_lumbricoides
Ascaris_suum
Brugia_malayi
Brugia_pahangi
Dirofilaria_immitis
Dracunculus_medinensis
Elaeophora_elaphi
Litomosoides_sigmodontis
Loa_loa
Onchocerca_ochengi
Onchocerca_volvulus
Parascaris_equorum
Parascaris_univalens
Thelazia_callipaeda
Toxocara_canis
Wuchereria_bancrofti
Acanthocheilonema_viteae
Gongylonema_pulchrum
Onchocerca_flexuosa
Ancylostoma_caninum
Ancylostoma_ceylanicum
Ancylostoma_duodenale
Angiostrongylus_cantonensis
Angiostrongylus_costaricensis
Caenorhabditis_brenneri
Caenorhabditis_briggsae
Caenorhabditis_elegans
Caenorhabditis_inopinata
Caenorhabditis_japonica
Caenorhabditis_latens
Caenorhabditis_nigoni
Caenorhabditis_remanei
Dictyocaulus_viviparus
Diploscapter_coronatus
Diploscapter_pachys
Haemonchus_contortus
Haemonchus_placei
Heligmosomoides_polygyrus
Heterorhabditis_bacteriophora
Mesorhabditis_belari
Micoletzkya_japonica
Necator_americanus
Nippostrongylus_brasiliensis
Oesophagostomum_dentatum
Oscheius_tipulae
Parapristionchus_giblindavisi
Pristionchus_arcanus
Pristionchus_entomophagus
Pristionchus_exspectatus
Pristionchus_fissidentatus
Pristionchus_japonicus
Pristionchus_maxplancki
Pristionchus_mayeri
Pristionchus_pacificus
Teladorsagia_circumcincta
Caenorhabditis_sinica
Caenorhabditis_tropicalis
Strongylus_vulgaris
Bursaphelenchus_xylophilus
Halicephalobus_mephisto
Panagrellus_redivivus
Steinernema_carpocapsae
Steinernema_feltiae
Steinernema_glaseri
Steinernema_monticolum
Steinernema_scapterisci
Strongyloides_papillosus
Strongyloides_venezuelensis
Strongyloides_ratti
Strongyloides_stercoralis
Acrobeloides_nanus
Ditylenchus_destructor
Globodera_rostochiensis
Heterodera_glycines
Meloidogyne_arenaria
Meloidogyne_enterolobii
Meloidogyne_floridensis
Meloidogyne_graminicola
Meloidogyne_hapla
Meloidogyne_incognita
Meloidogyne_javanica
Ditylenchus_dipsaci

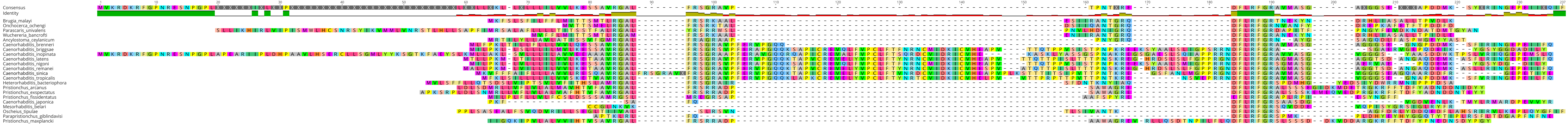
Consensus
Identity
Brugia_malayi
Onchocerca_ochengi
Parascaris_univalens
Wuchereria_bancrofti
Ancylostoma_ceylanicum
Caenorhabditis_brenneri
Caenorhabditis_briggsae
Caenorhabditis_inopinata
Caenorhabditis_latens
Caenorhabditis_nigoni
Caenorhabditis_remanei
Caenorhabditis_sinica
Caenorhabditis_tropicalis
Heterorhabditis_bacteriophora
Pristionchus_arcanus
Pristionchus_exspectatus
Pristionchus_fissidentatus
Caenorhabditis_japonica
Mesorhabditis_belari
Oscheius_tipulae
Parapristionchus_giblindavisi
Pristionchus_maxplancki

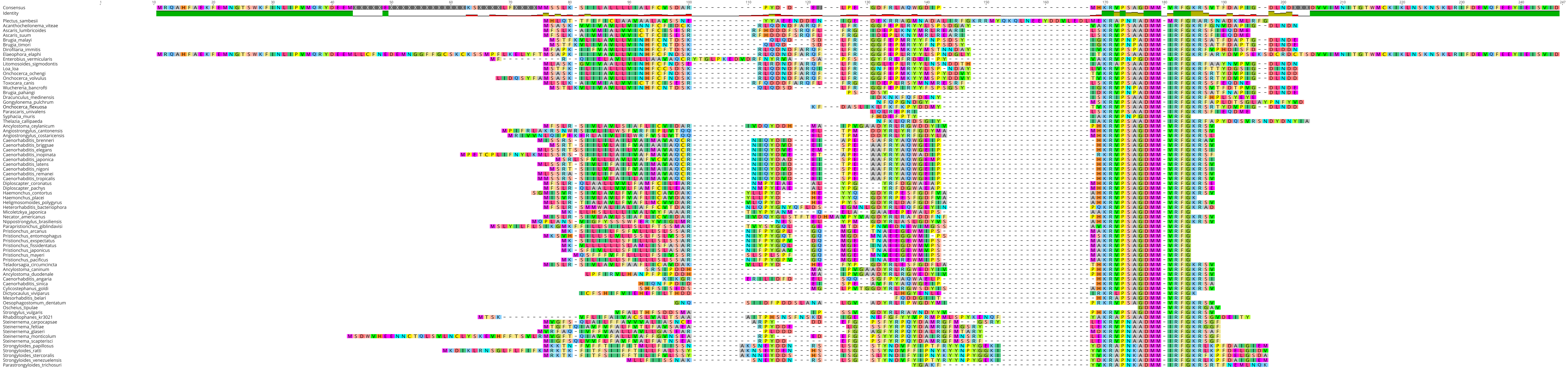
Consensus
Identity
Plectus_sambesii
Acanthocheilonema_viteae
Ascaris_lumbricoides
Ascaris_suum
Brugia_malayi
Brugia_timori
Dirofilaria_immitis
Elaeophora_elaphi
Enterobius_vermicularis
Litomosoides_sigmodontis
Loa_loa
Onchocerca_ochengi
Onchocerca_volvulus
Toxocara_canis
Wuchereria_bancrofti
Brugia_pahangi
Dracunculus_medinensis
Gongylonema_pulchrum
Onchocerca_flexuosa
Parascaris_univalens
Syphacia_muris
Thelazia_callipaeda
Ancylostoma_ceylanicum
Angiostrongylus_cantonensis
Angiostrongylus_costaricensis
Caenorhabditis_brenneri
Caenorhabditis_briggsae
Caenorhabditis_elegans
Caenorhabditis_inopinata
Caenorhabditis_japonica
Caenorhabditis_latens
Caenorhabditis_nigoni
Caenorhabditis_remanei
Caenorhabditis_tropicalis
Diploscapter_coronatus
Diploscapter_pachys
Haemonchus_contortus
Haemonchus_placei
Heligmosomoides_polygyrus
Heterorhabditis_bacteriophora
Micoletzkya_japonica
Necator_americanus
Nippostrongylus_brasiliensis
Parapristionchus_giblindavisi
Pristionchus_arcanus
Pristionchus_entomophagus
Pristionchus_exspectatus
Pristionchus_fissidentatus
Pristionchus_japonicus
Pristionchus_mayeri
Pristionchus_pacificus
Teladorsagia_circumcincta
Ancylostoma_caninum
Ancylostoma_duodenale
Caenorhabditis_angaria
Caenorhabditis_sinica
Cylicostephanus_goldi
Dictyocaulus_viviparus
Mesorhabditis_belari
Oesophagostomum_dentatum
Oscheius_tipulae
Strongylus_vulgaris
Rhabditophanes_kr3021
Steinernema_carpocapsae
Steinernema_feltiae
Steinernema_glaseri
Steinernema_monticolum
Steinernema_scapterisci
Strongyloides_papillosus
Strongyloides_ratti
Strongyloides_stercoralis
Strongyloides_venezuelensis
Parastrongyloides_trichosuri

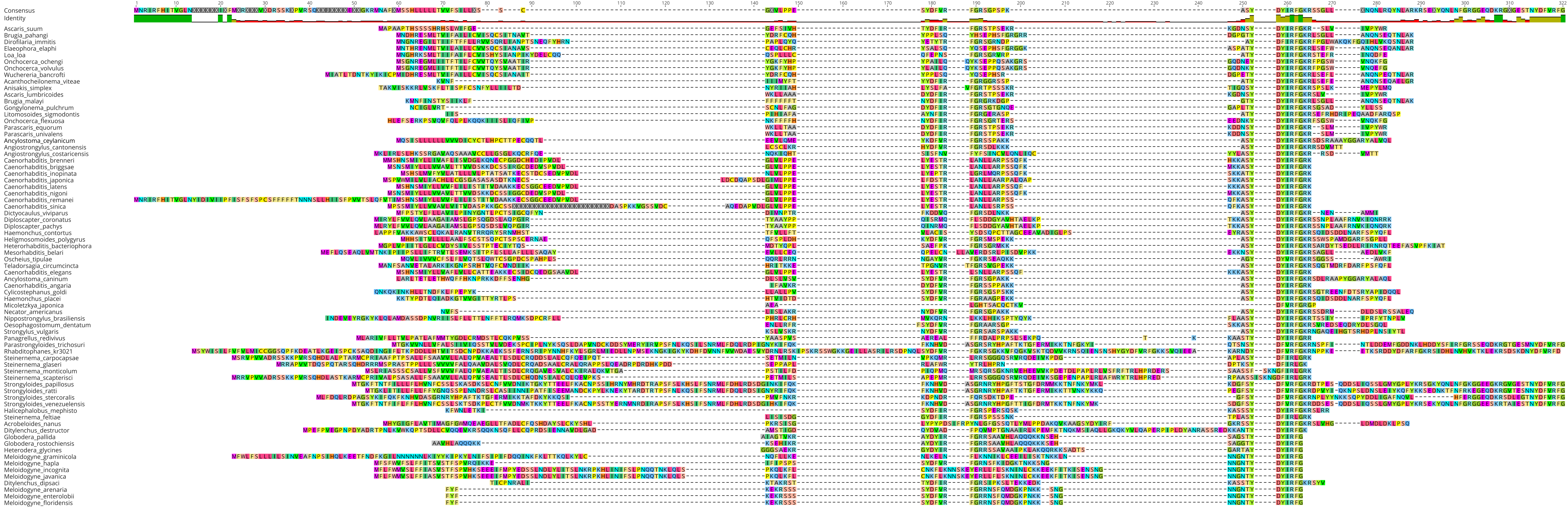
Consensus
Identity
Ascaris_suum
Brugia_pahangi
Dirofilaria_immitis
Elaeophora_elaphi
Loa_loa
Onchocerca_ochengi
Onchocerca_volvulus
Wuchereria_bancrofti
Acanthocheilonema_viteae
Anisakis_simplex
Ascaris_lumbricoides
Brugia_malayi
Gongylonema_pulchrum
Litomosoides_sigmodontis
Onchocerca_flexuosa
Parascaris_equorum
Parascaris_univalens
Ancylostoma_ceylanicum
Angiostrongylus_cantonensis
Angiostrongylus_costaricensis
Caenorhabditis_brenneri
Caenorhabditis_briggsae
Caenorhabditis_inopinata
Caenorhabditis_japonica
Caenorhabditis_latens
Caenorhabditis_nigoni
Caenorhabditis_remanei
Caenorhabditis_sinica
Dictyocaulus_viviparus
Diploscapter_coronatus
Diploscapter_pachys
Haemonchus_contortus
Heligmosomoides_polygyrus
Heterorhabditis_bacteriophora
Mesorhabditis_belari
Oscheius_tipulae
Teladorsagia_circumcincta
Caenorhabditis_elegans
Ancylostoma_caninum
Caenorhabditis_angaria
Cylicostephanus_goldi
Haemonchus_placei
Micoletzkya_japonica
Necator_americanus
Nippostrongylus_brasiliensis
Oesophagostomum_dentatum
Strongylus_vulgaris
Panagrellus_redivivus
Parastrongyloides_trichosuri
Rhabditophanes_kr3021
Steinernema_carpocapsae
Steinernema_glaseri
Steinernema_monticolum
Steinernema_scapterisci
Strongyloides_papillosus
Strongyloides_ratti
Strongyloides_stercoralis
Strongyloides_venezuelensis
Halicephalobus_mephisto
Steinernema_feltiae
Acrobeloides_nanus
Ditylenchus_destructor
Globodera_pallida
Globodera_rostochiensis
Heterodera_glycines
Meloidogyne_graminicola
Meloidogyne_hapla
Meloidogyne_incognita
Meloidogyne_javanica
Ditylenchus_dipsaci
Meloidogyne_arenaria
Meloidogyne_enterolobii
Meloidogyne_floridensis

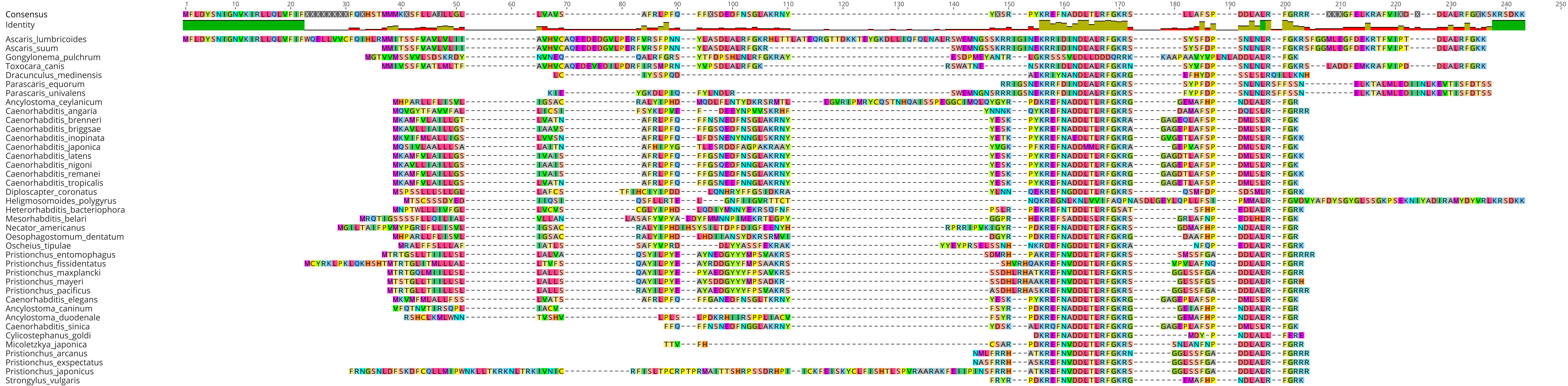
Consensus
Identity
Ascaris_lumbricoides
Ascaris_suum
Gongylonema_pulchrum
Toxocara_canis
Dracunculus_medinensis
Parascaris_equorum
Parascaris_univalens
Ancylostoma_ceylanicum
Caenorhabditis_angaria
Caenorhabditis_brenneri
Caenorhabditis_briggsae
Caenorhabditis_inopinata
Caenorhabditis_japonica
Caenorhabditis_latens
Caenorhabditis_nigoni
Caenorhabditis_remanei
Caenorhabditis_tropicalis
Diploscapter_coronatus
Heligmosomoides_polygyrus
Heterorhabditis_bacteriophora
Mesorhabditis_belari
Necator_americanus
Oesophagostomum_dentatum
Oscheius_tipulae
Pristionchus_entomophagus
Pristionchus_fissidentatus
Pristionchus_maxplancki
Pristionchus_mayeri
Pristionchus_pacificus
Caenorhabditis_elegans
Ancylostoma_caninum
Ancylostoma_duodenale
Caenorhabditis_sinica
Cylicostephanus_goldi
Micoletzkya_japonica
Pristionchus_arcanus
Pristionchus_exspectatus
Pristionchus_japonicus
Strongylus_vulgaris

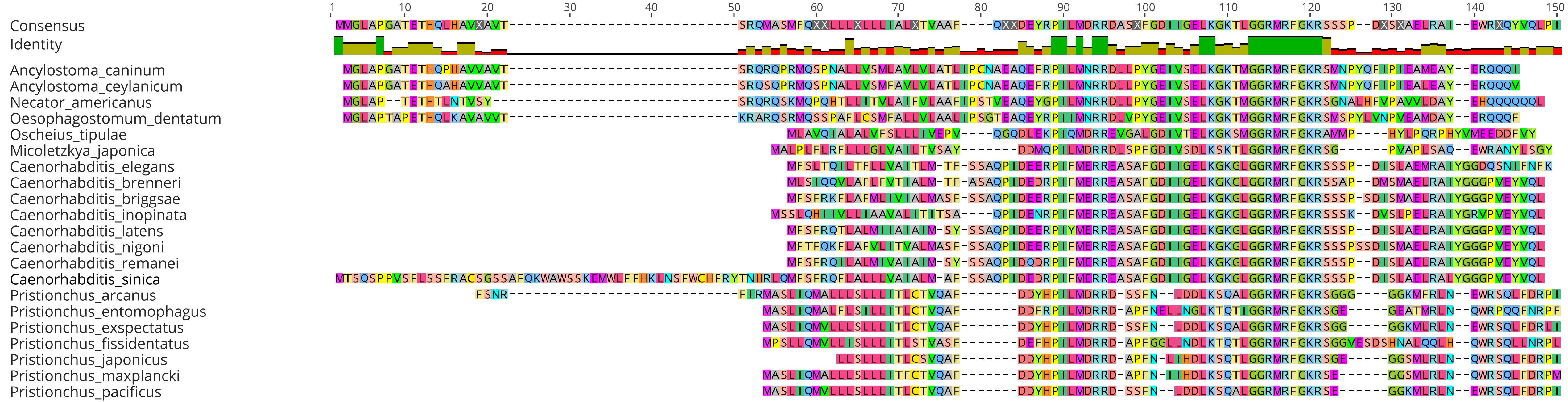

1 10 20 30 40 50 60 70 80 90 100 110 120 130 140 150
Consensus
Identity
Ancylostoma_caninum
Ancylostoma_ceylanicum
Necator_americanus
Oesophagostomum_dentatum
Oscheius_tipulae
Micoletzkya_japonica
Caenorhabditis_elegans
Caenorhabditis_brenneri
Caenorhabditis_briggsae
Caenorhabditis_inopinata
Caenorhabditis_latens
Caenorhabditis_nigoni
Caenorhabditis_remanei
Caenorhabditis_sinica
Pristionchus_arcanus
Pristionchus_entomophagus
Pristionchus_exspectatus
Pristionchus_fissidentatus
Pristionchus_japonicus
Pristionchus_maxplancki
Pristionchus_pacificus

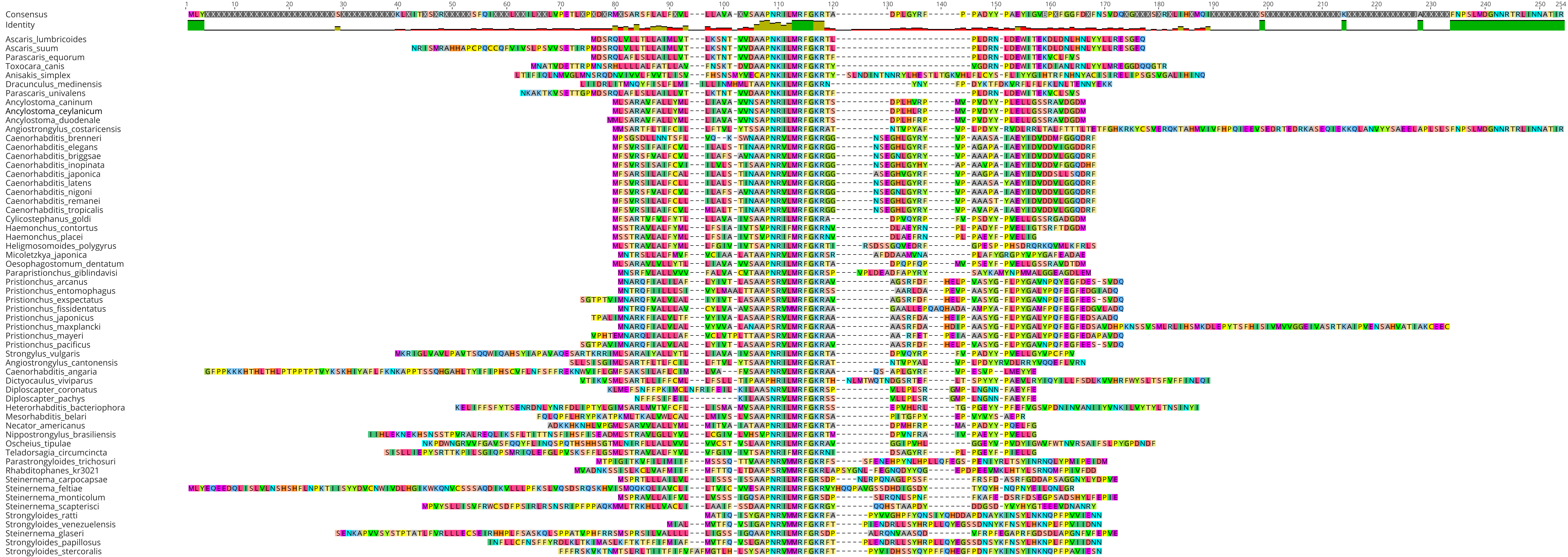
Consensus
Identity
1 10 20 30 40 50 60 70 80 90 100 110 120 130 140 150 160 170 180 190 200 210 220 230 240 250 254
Ascaris_lumbricoides
Ascaris_suum
Parascaris_equorum
Toxocara_canis
Anisakis_simplex
Dracunculus_medinensis
Parascaris_univalens
Ancylostoma_caninum
Ancylostoma_ceylanicum
Ancylostoma_duodenale
Angiostrongylus_costaricensis
Caenorhabditis_brenneri
Caenorhabditis_elegans
Caenorhabditis_briggsae
Caenorhabditis_inopinata
Caenorhabditis_japonica
Caenorhabditis_latens
Caenorhabditis_nigoni
Caenorhabditis_remanei
Caenorhabditis_tropicalis
Cylicostephanus_goldi
Haemonchus_contortus
Haemonchus_placei
Heligmosomoides_polygyrus
Micoletzkya_japonica
Oesophagostomum_dentatum
Parapristionchus_giblindavisi
Pristionchus_arcanus
Pristionchus_entomophagus
Pristionchus_exspectatus
Pristionchus_fissidentatus
Pristionchus_japonicus
Pristionchus_maxplancki
Pristionchus_mayeri
Pristionchus_pacificus
Strongylus_vulgaris
Angiostrongylus_cantonensis
Caenorhabditis_angaria
Dictyocaulus_viviparus
Diploscapter_coronatus
Diploscapter_pachys
Heterorhabditis_bacteriophora
Mesorhabditis_belari
Necator_americanus
Nippostrongylus_brasiliensis
Oscheius_tipulae
Teladorsagia_circumcincta
Parastrongyloides_trichosuri
Rhabditophanes_kr3021
Steinernema_carpocapsae
Steinernema_feltiae
Steinernema_monticolum
Steinernema_scapterisci
Strongyloides_ratti
Strongyloides_venezuelensis
Steinernema_glaseri
Strongyloides_papillosus
Strongyloides_stercoralis

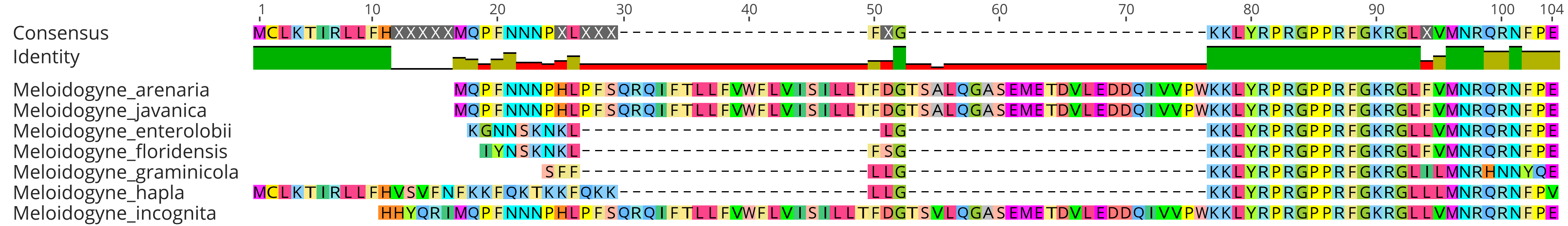

1
10
20
30
40
50
60
70
80
90
100
104
Consensus
Identity
Meloidogyne_arenaria
Meloidogyne_javanica
Meloidogyne_enterolobii
Meloidogyne_floridensis
Meloidogyne_graminicola
Meloidogyne_hapla
Meloidogyne_incognita

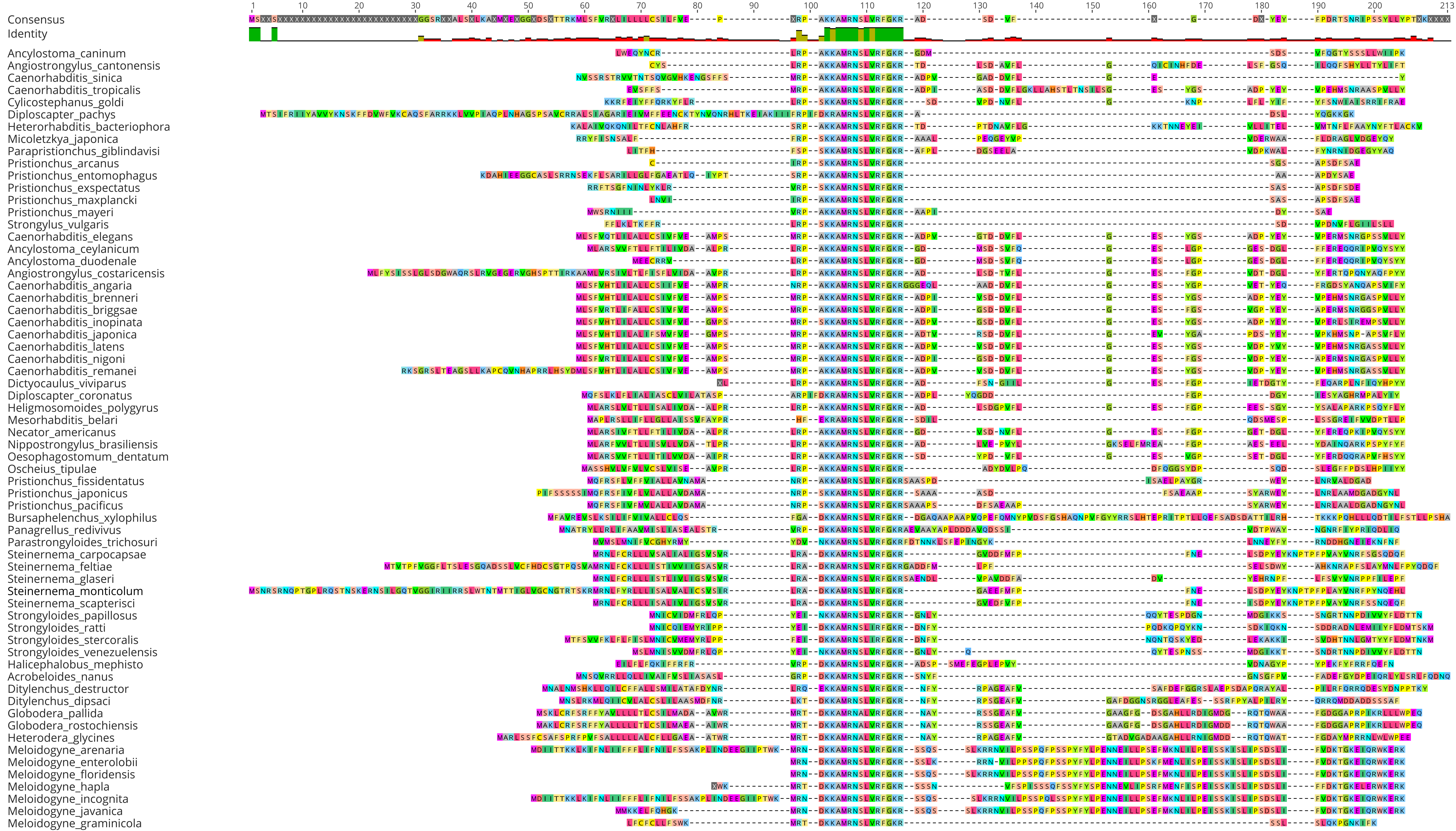

1 10 20 30 40 50 60 70 80 90 100 110 120 130 140 150 160 170 180 190 200 213
Consensus
Identity
Ancylostoma_caninum
Angiostrongylus_cantonensis
Caenorhabditis_sinica
Caenorhabditis_tropicalis
Cylicostephanus_goldi
Diploscapter_pachys
Heterorhabditis_bacteriophora
Micoletzkya_japonica
Parapristionchus_giblindavisi
Pristionchus_arcanus
Pristionchus_entomophagus
Pristionchus_exspectatus
Pristionchus_maxplancki
Pristionchus_mayeri
Strongylus_vulgaris
Caenorhabditis_elegans
Ancylostoma_ceylanicum
Ancylostoma_duodenale
Angiostrongylus_costaricensis
Caenorhabditis_angaria
Caenorhabditis_brenneri
Caenorhabditis_briggsae
Caenorhabditis_inopinata
Caenorhabditis_japonica
Caenorhabditis_latens
Caenorhabditis_nigoni
Caenorhabditis_remanei
Dictyocaulus_viviparus
Diploscapter_coronatus
Heligmosomoides_polygyrus
Mesorhabditis_belari
Necator_americanus
Nippostrongylus_brasiliensis
Oesophagostomum_dentatum
Oscheius_tipulae
Pristionchus_fissidentatus
Pristionchus_japonicus
Pristionchus_pacificus
Bursaphelenchus_xylophilus
Panagrellus_redivivus
Parastrongyloides_trichosuri
Steinernema_carpocapsae
Steinernema_feltiae
Steinernema_glaseri
Steinernema_monticolum
Steinernema_scapterisci
Strongyloides_papillosus
Strongyloides_ratti
Strongyloides_stercoralis
Strongyloides_venezuelensis
Halicephalobus_mephisto
Acrobeloides_nanus
Ditylenchus_destructor
Ditylenchus_dipsaci
Globodera_pallida
Globodera_rostochiensis
Heterodera_glycines
Meloidogyne_arenaria
Meloidogyne_enterolobii
Meloidogyne_floridensis
Meloidogyne_hapla
Meloidogyne_incognita
Meloidogyne_javanica
Meloidogyne_graminicola

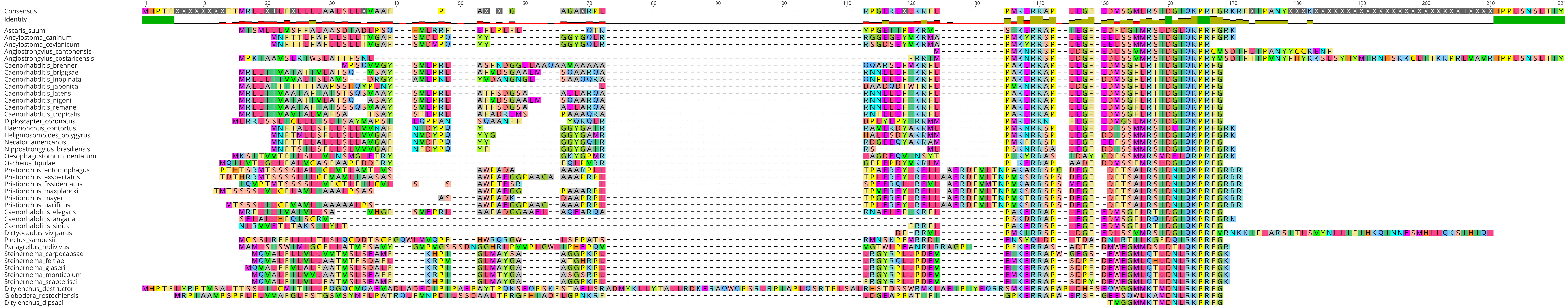
Consensus
Identity
Ascaris_suum
Ancylostoma_caninum
Ancylostoma_ceylanicum
Angiostrongylus_cantonensis
Angiostrongylus_costaricensis
Caenorhabditis_brenneri
Caenorhabditis_briggsae
Caenorhabditis_inopinata
Caenorhabditis_japonica
Caenorhabditis_latens
Caenorhabditis_nigoni
Caenorhabditis_remanei
Caenorhabditis_tropicalis
Diploscapter_coronatus
Haemonchus_contortus
Heligmosomoides_polygyrus
Necator_americanus
Nippostrongylus_brasiliensis
Oesophagostomum_dentatum
Oscheius_tipulae
Pristionchus_entomophagus
Pristionchus_exspectatus
Pristionchus_fissidentatus
Pristionchus_maxplancki
Pristionchus_mayeri
Pristionchus_pacificus
Caenorhabditis_elegans
Caenorhabditis_angaria
Caenorhabditis_sinica
Dictyocaulus_viviparus
Plectus_sambesii
Panagrellus_redivivus
Steinernema_carpocapsae
Steinernema_feltiae
Steinernema_glaseri
Steinernema_monticolum
Steinernema_scapterisci
Ditylenchus_destructor
Globodera_rostochiensis
Ditylenchus_dipsaci

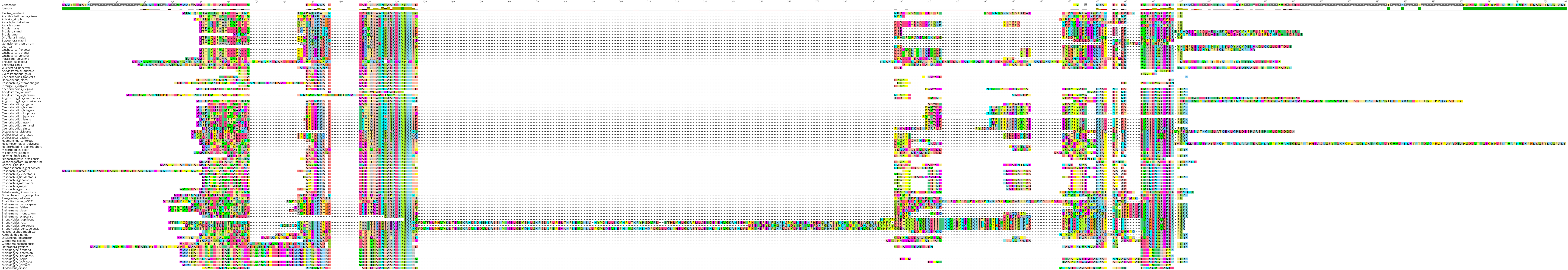

Consensus
Identity
Plectus_sambesii
Acanthocheilonema_viteae
Anisakis_simplex
Ascaris_lumbricoides
Ascaris_suum
Brugia_malayi
Brugia_pahangi
Brugia_timori
Dirofilaria_immitis
Elaeophora_elaphi
Gongylonema_pulchrum
Loa_loa
Onchocerca_flexuosa
Onchocerca_ochengi
Onchocerca_volvulus
Parascaris_univalens
Thelazia_callipaeda
Toxocara_canis
Wuchereria_bancrofti
Ancylostoma_duodenale
Cylicostephanus_goldi
Caenorhabditis_tropicalis
Haemonchus_placei
Pristionchus_entomophagus
Strongylus_vulgaris
Caenorhabditis_elegans
Ancylostoma_caninum
Ancylostoma_ceylanicum
Angiostrongylus_cantonensis
Angiostrongylus_costaricensis
Caenorhabditis_angaria
Caenorhabditis_brenneri
Caenorhabditis_briggsae
Caenorhabditis_inopinata
Caenorhabditis_japonica
Caenorhabditis_latens
Caenorhabditis_nigoni
Caenorhabditis_remanei
Caenorhabditis_sinica
Dictyocaulus_viviparus
Diploscapter_coronatus
Diploscapter_pachys
Haemonchus_contortus
Heligmosomoides_polygyrus
Heterorhabditis_bacteriophora
Mesorhabditis_belari
Micoletzkya_japonica
Necator_americanus
Nippostrongylus_brasiliensis
Oesophagostomum_dentatum
Oscheius_tipulae
Parapristionchus_giblindavisi
Pristionchus_arcanus
Pristionchus_exspectatus
Pristionchus_fissidentatus
Pristionchus_japonicus
Pristionchus_maxplancki
Pristionchus_mayeri
Pristionchus_pacificus
Teladorsagia_circumcincta
Bursaphelenchus_xylophilus
Panagrellus_redivivus
Rhabditophanes_kr3021
Steinernema_carpocapsae
Steinernema_feltiae
Steinernema_glaseri
Steinernema_monticolum
Steinernema_scapterisci
Strongyloides_papillosus
Strongyloides_ratti
Strongyloides_stercoralis
Strongyloides_venezuelensis
Halicephalobus_mephisto
Acrobeloides_nanus
Ditylenchus_destructor
Globodera_pallida
Globodera_rostochiensis
Heterodera_glycines
Meloidogyne_arenaria
Meloidogyne_enterolobii
Meloidogyne_floridensis
Meloidogyne_hapla
Meloidogyne_incognita
Meloidogyne_javanica
Ditylenchus_dipsaci
