# Supplementary material for: Exploitation of phylum-spanning omics resources reveals complexity in the nematode FLP signalling system and provides insights into flp-gene evolution
Source: BMC Genomics. 2024 Dec 19;25:1220. doi: 10.1186/s12864-024-11111-6 (PMC11658156; doi:10.1186/s12864-024-11111-6)
Supplement: Supplementary file 6 — Supplementary Material 6 [file 12864_2024_11111_MOESM6_ESM.pdf]

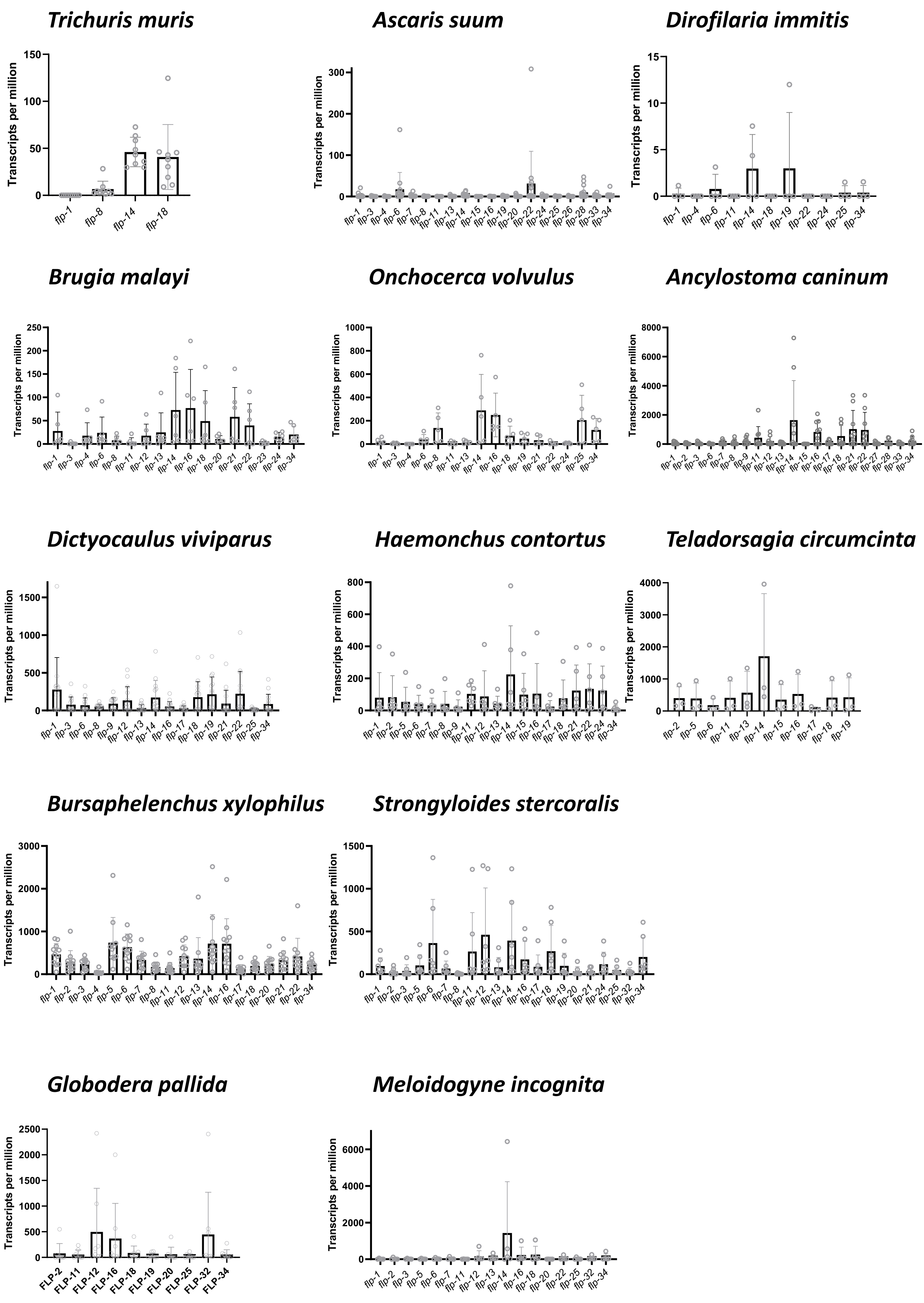
Trichuris muris
Transcripts per million
0 50 100 150
flp-1 flp-8 flp-14 flp-18
Ascaris suum
Transcripts per million
0 100 200 300
flp-1 flp-3 flp-4 flp-6 flp-7 flp-8 flp-11 flp-13 flp-14 flp-15 flp-16 flp-19 flp-20 flp-22 flp-24 flp-25 flp-26 flp-28 flp-33 flp-34
Dirofilaria immitis
Transcripts per million
0 5 10 15
flp-1 flp-4 flp-6 flp-11 flp-14 flp-18 flp-19 flp-22 flp-24 flp-25 flp-34
Brugia malayi
Transcripts per million
0 50 100 150 200 250
flp-1 flp-3 flp-4 flp-6 flp-8 flp-11 flp-12 flp-13 flp-14 flp-16 flp-18 flp-20 flp-21 flp-22 flp-23 flp-24 flp-34
Onchocerca volvulus
Transcripts per million
0 200 400 600 800 1000
flp-1 flp-3 flp-4 flp-6 flp-8 flp-11 flp-13 flp-14 flp-16 flp-18 flp-19 flp-21 flp-22 flp-24 flp-25 flp-34
Ancylostoma caninum
Transcripts per million
0 2000 4000 6000 8000
flp-1 flp-2 flp-3 flp-6 flp-7 flp-8 flp-9 flp-11 flp-12 flp-13 flp-14 flp-15 flp-16 flp-17 flp-18 flp-21 flp-22 flp-27 flp-28 flp-33 flp-34
Dictyocaulus viviparus
Transcripts per million
0 500 1000 1500
flp-1 flp-3 flp-6 flp-8 flp-9 flp-12 flp-13 flp-14 flp-16 flp-17 flp-18 flp-19 flp-21 flp-22 flp-25 flp-34
Haemonchus contortus
Transcripts per million
0 200 400 600 800
flp-1 flp-2 flp-5 flp-6 flp-7 flp-8 flp-9 flp-11 flp-12 flp-13 flp-14 flp-15 flp-16 flp-17 flp-18 flp-21 flp-22 flp-24 flp-34
Teladorsagia circumcinta
Transcripts per million
0 1000 2000 3000 4000
flp-2 flp-5 flp-6 flp-11 flp-13 flp-14 flp-15 flp-16 flp-17 flp-18 flp-19
Bursaphelenchus xylophilus
Transcripts per million
0 1000 2000 3000
flp-1 flp-2 flp-3 flp-4 flp-5 flp-6 flp-7 flp-8 flp-11 flp-12 flp-13 flp-14 flp-16 flp-17 flp-18 flp-20 flp-21 flp-22 flp-34
Strongyloides stercoralis
Transcripts per million
0 500 1000 1500
flp-1 flp-2 flp-3 flp-5 flp-6 flp-7 flp-8 flp-11 flp-12 flp-13 flp-14 flp-16 flp-17 flp-18 flp-19 flp-20 flp-21 flp-24 flp-25 flp-32 flp-34
Globodera pallida
Transcripts per million
0 500 1000 1500 2000 2500
FLP-2 FLP-11 FLP-12 FLP-16 FLP-18 FLP-19 FLP-20 FLP-25 FLP-32 FLP-34
Meloidogyne incognita
Transcripts per million
0 2000 4000 6000
flp-1 flp-2 flp-3 flp-5 flp-6 flp-7 flp-11 flp-12 flp-13 flp-14 flp-16 flp-18 flp-20 flp-22 flp-25 flp-32 flp-34
