# Supplementary figures and images for: Exploitation of phylum-spanning omics resources reveals complexity in the nematode FLP signalling system and provides insights into flp-gene evolution
Source: BMC Genomics. 2024 Dec 19;25:1220. doi: 10.1186/s12864-024-11111-6 (PMC11658156; doi:10.1186/s12864-024-11111-6)

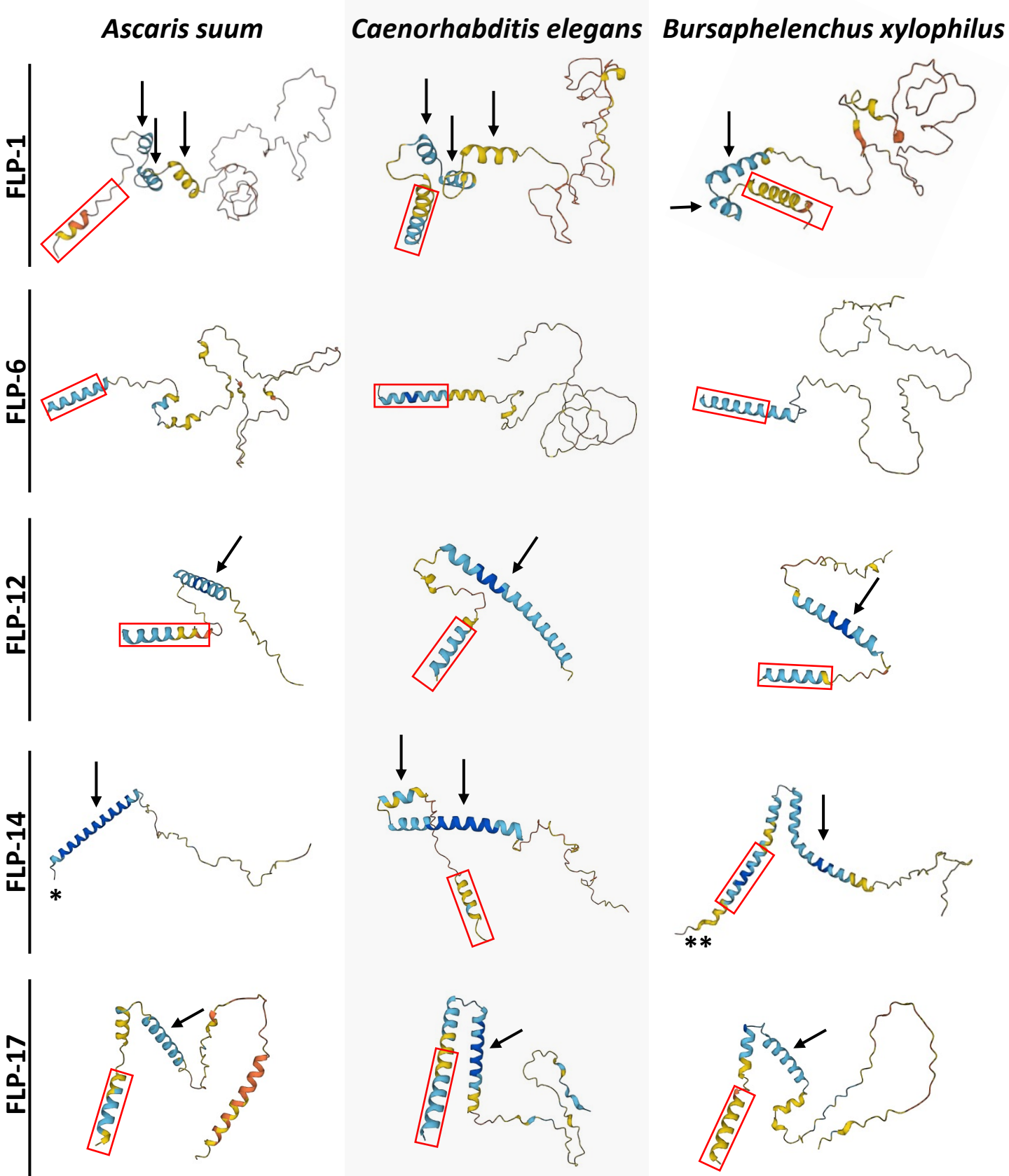

Model Confidence

Very high

High

Low

Very low

Supplement: Supplementary file 7 — Supplementary Material 7 [file 12864_2024_11111_MOESM7_ESM.pdf]

# FLP-17

# FLP-6

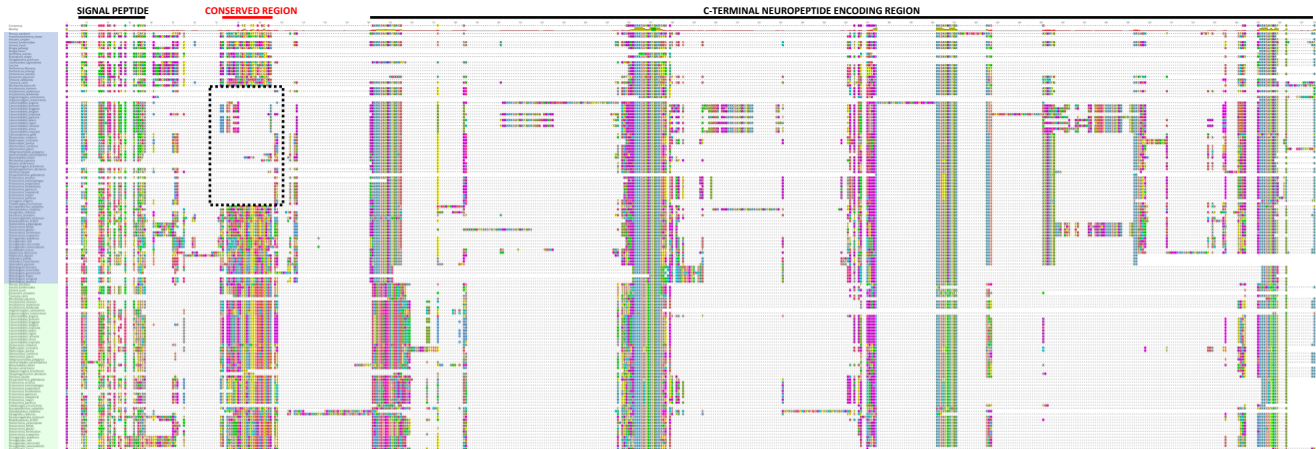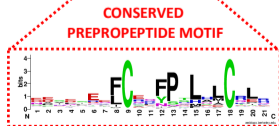

Supplement: Supplementary file 8 — Supplementary Material 8 [file 12864_2024_11111_MOESM8_ESM.pdf]
