# Supplementary material for: Exploitation of phylum-spanning omics resources reveals complexity in the nematode FLP signalling system and provides insights into flp-gene evolution
Source: BMC Genomics. 2024 Dec 19;25:1220. doi: 10.1186/s12864-024-11111-6 (PMC11658156; doi:10.1186/s12864-024-11111-6)

FLP-1 peptide alignment

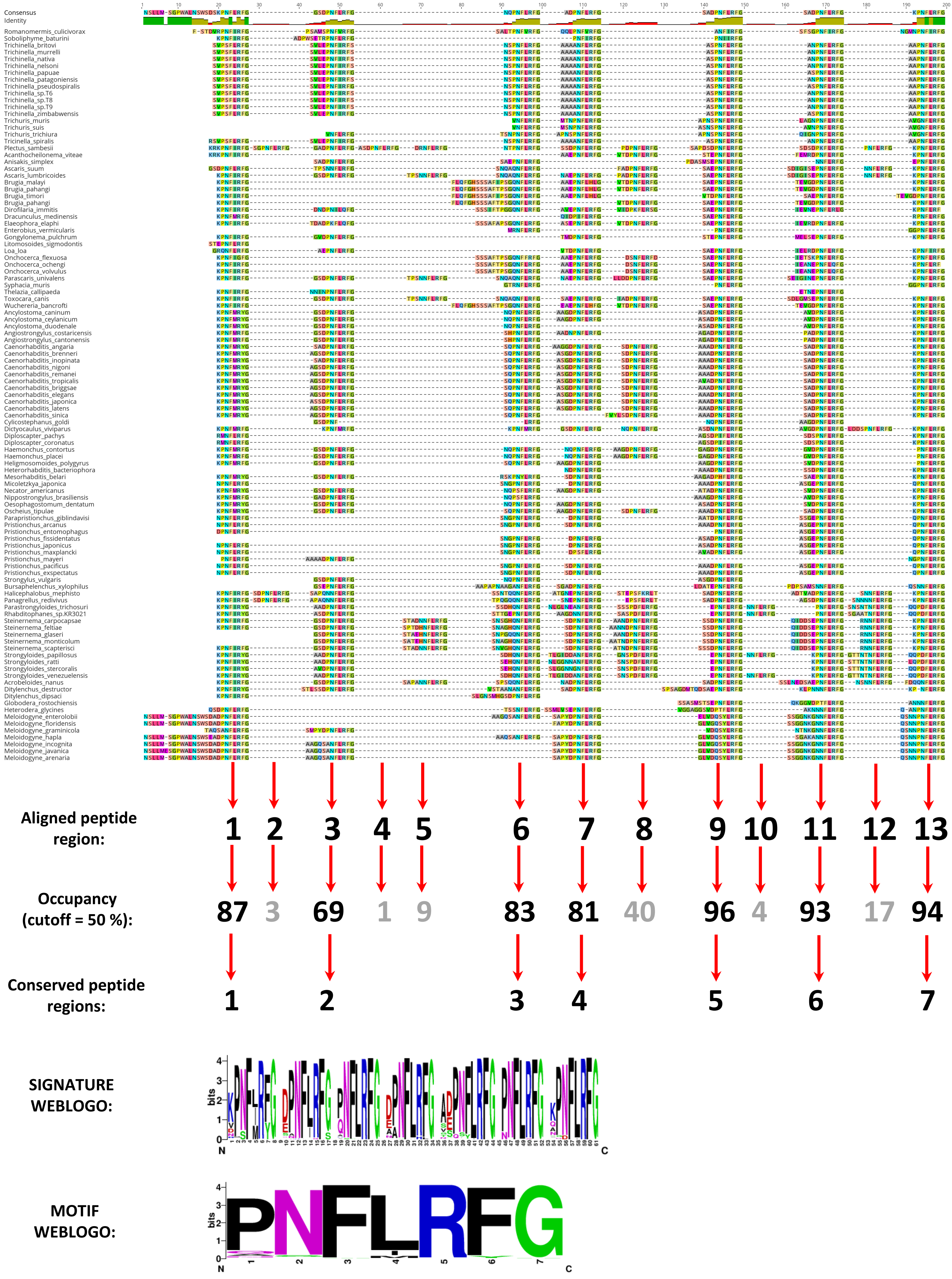

FLP-2 peptide alignment

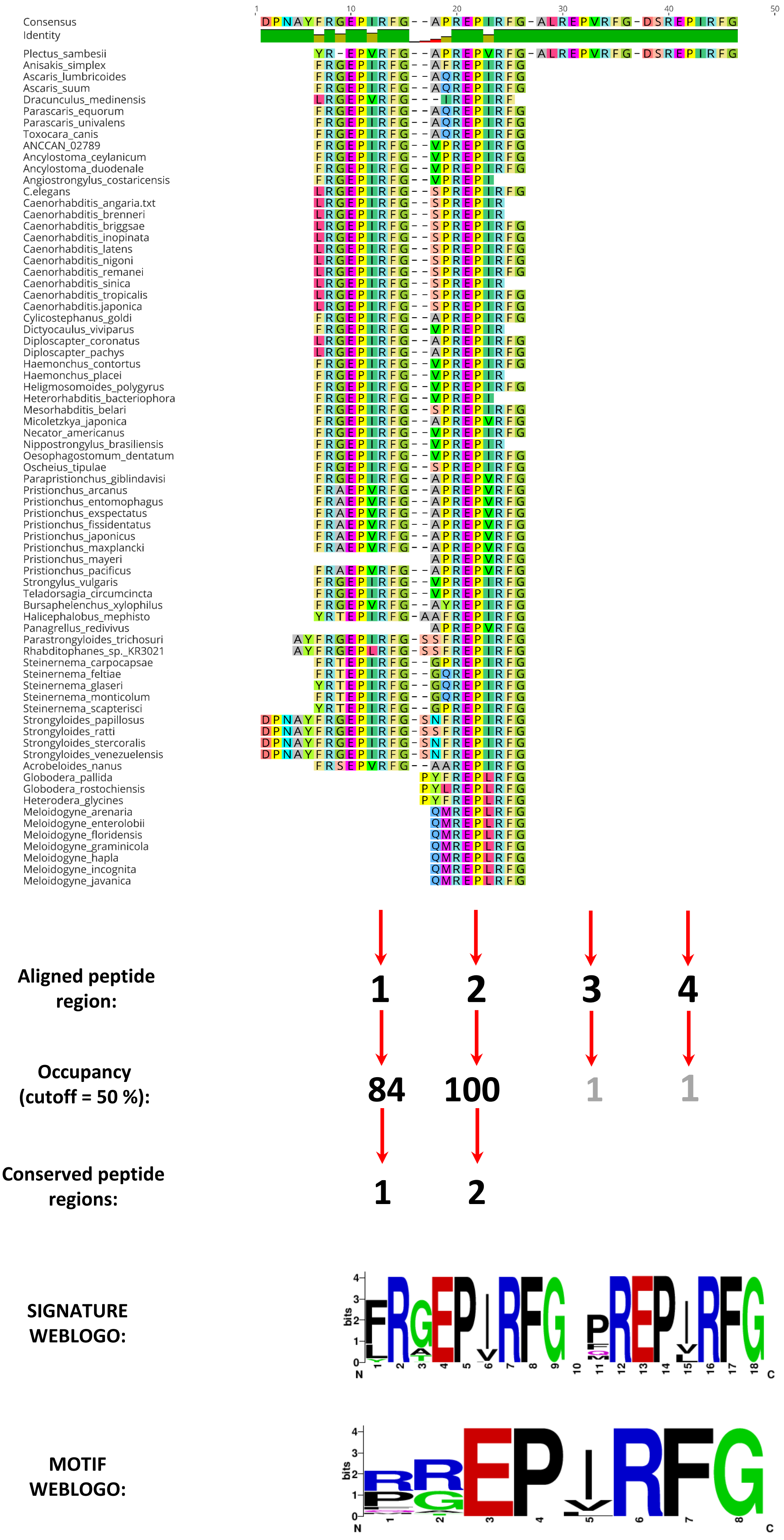

FLP-3 peptide alignment

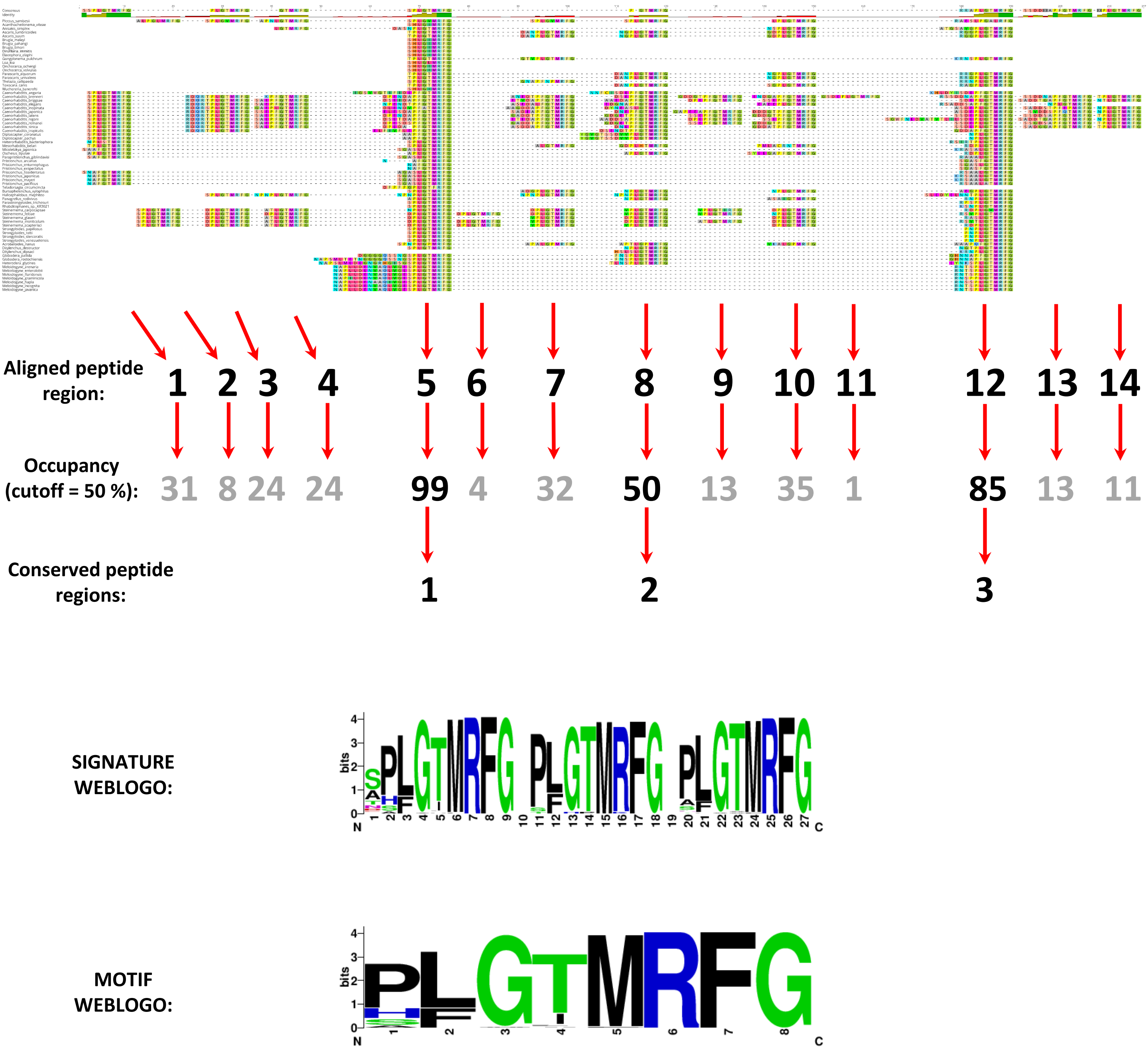

FLP-4 peptide alignment

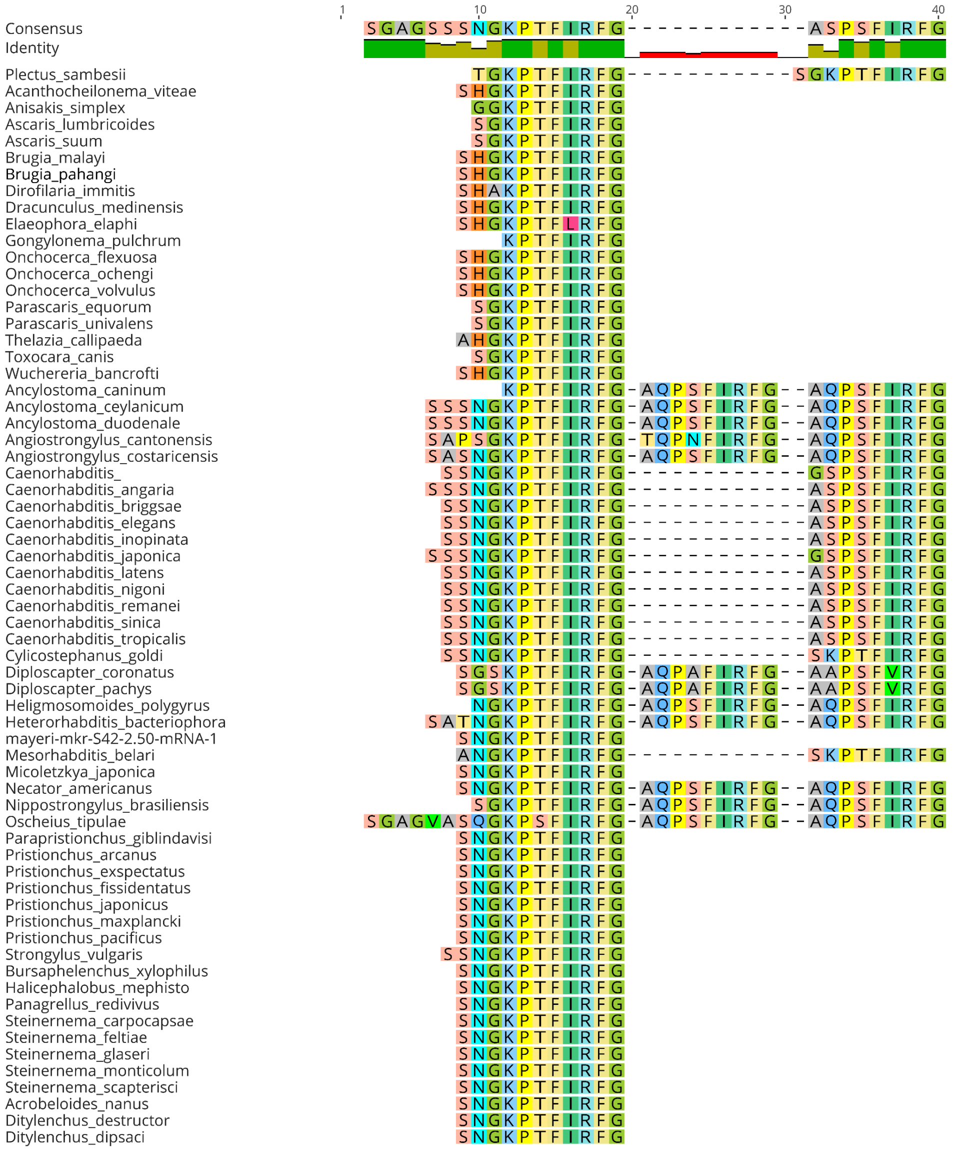

Aligned peptide region: 1 2 3

Occupancy (cutoff = 50 %): 84 18 40

Conserved peptide regions: 1

SIGNATURE/MOTIF WEBLOGO:

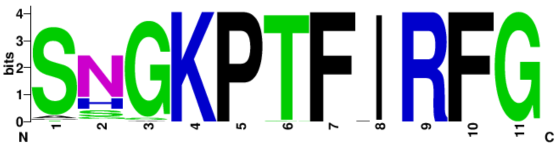

FLP-5 peptide alignment

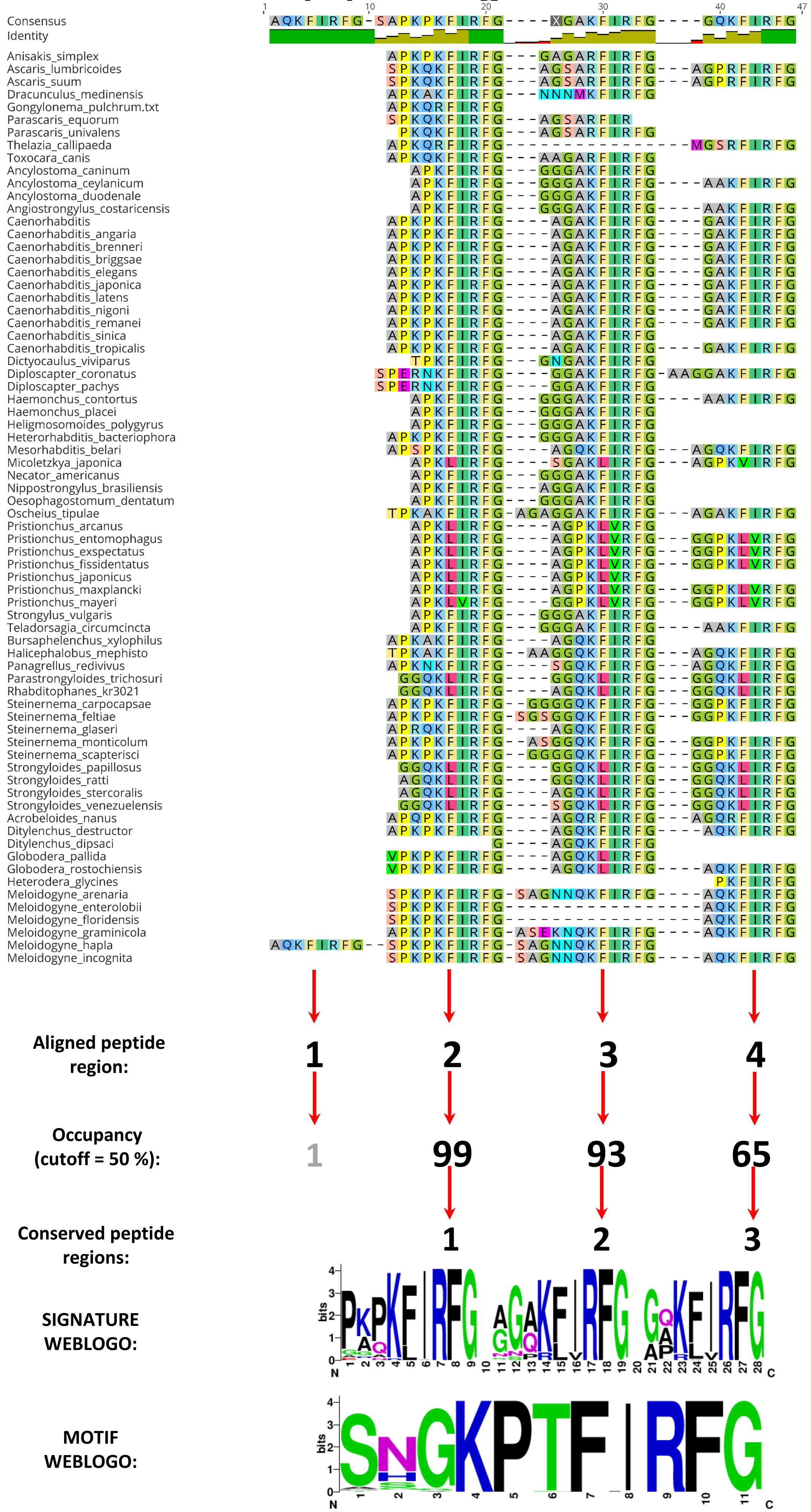

FLP-6 peptide alignment

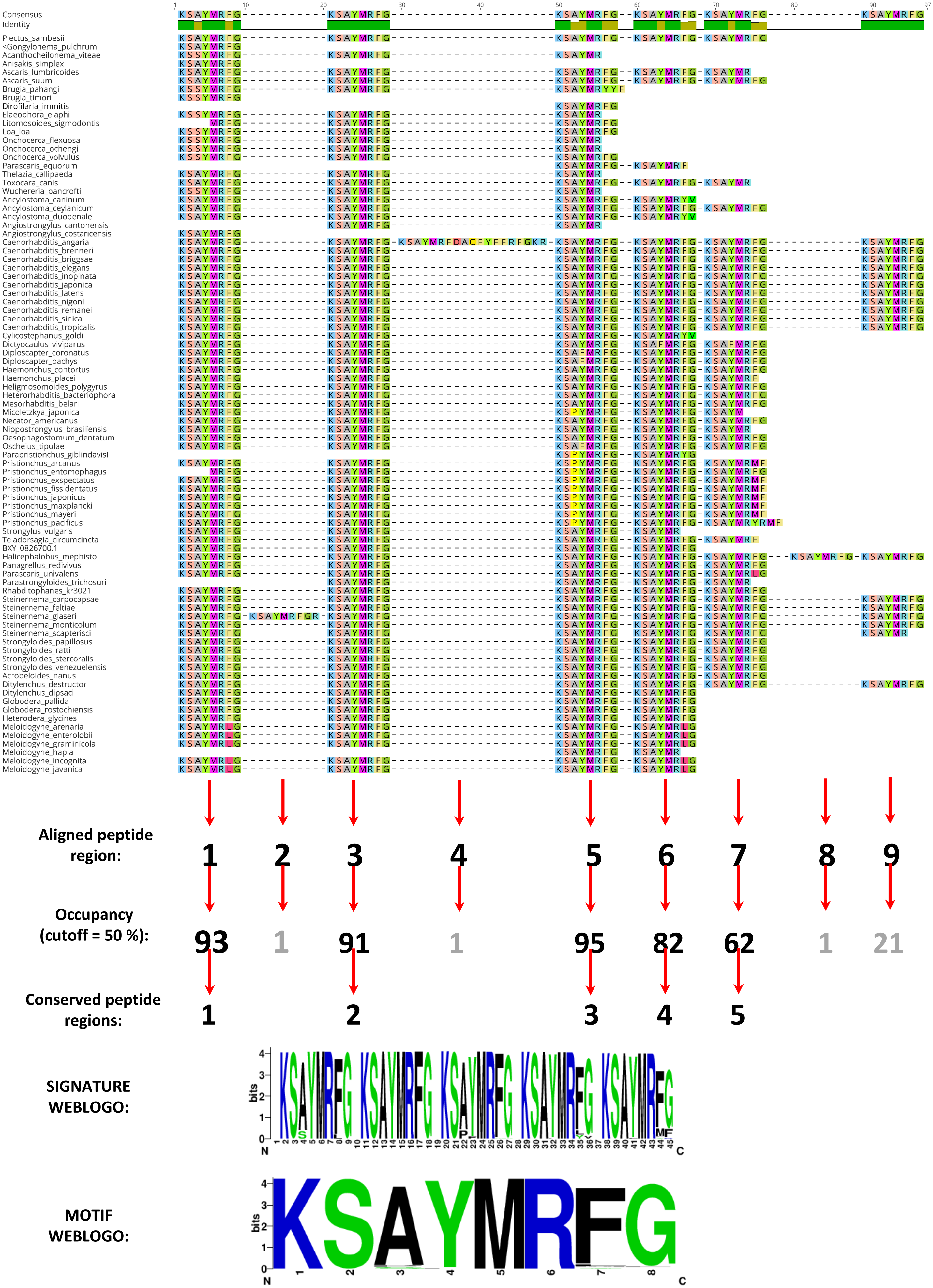

FLP-7 peptide alignment

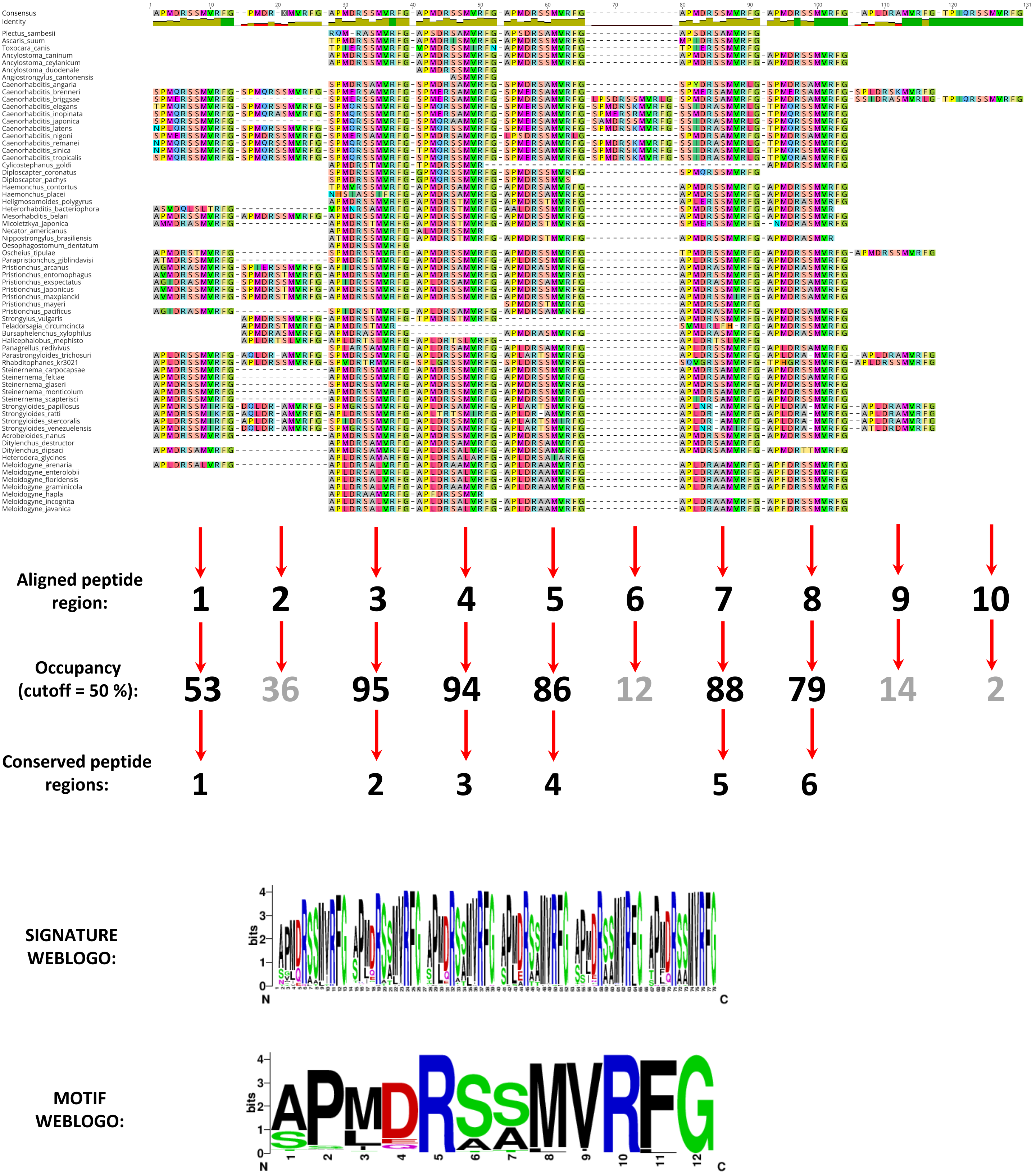

FLP-8 peptide alignment

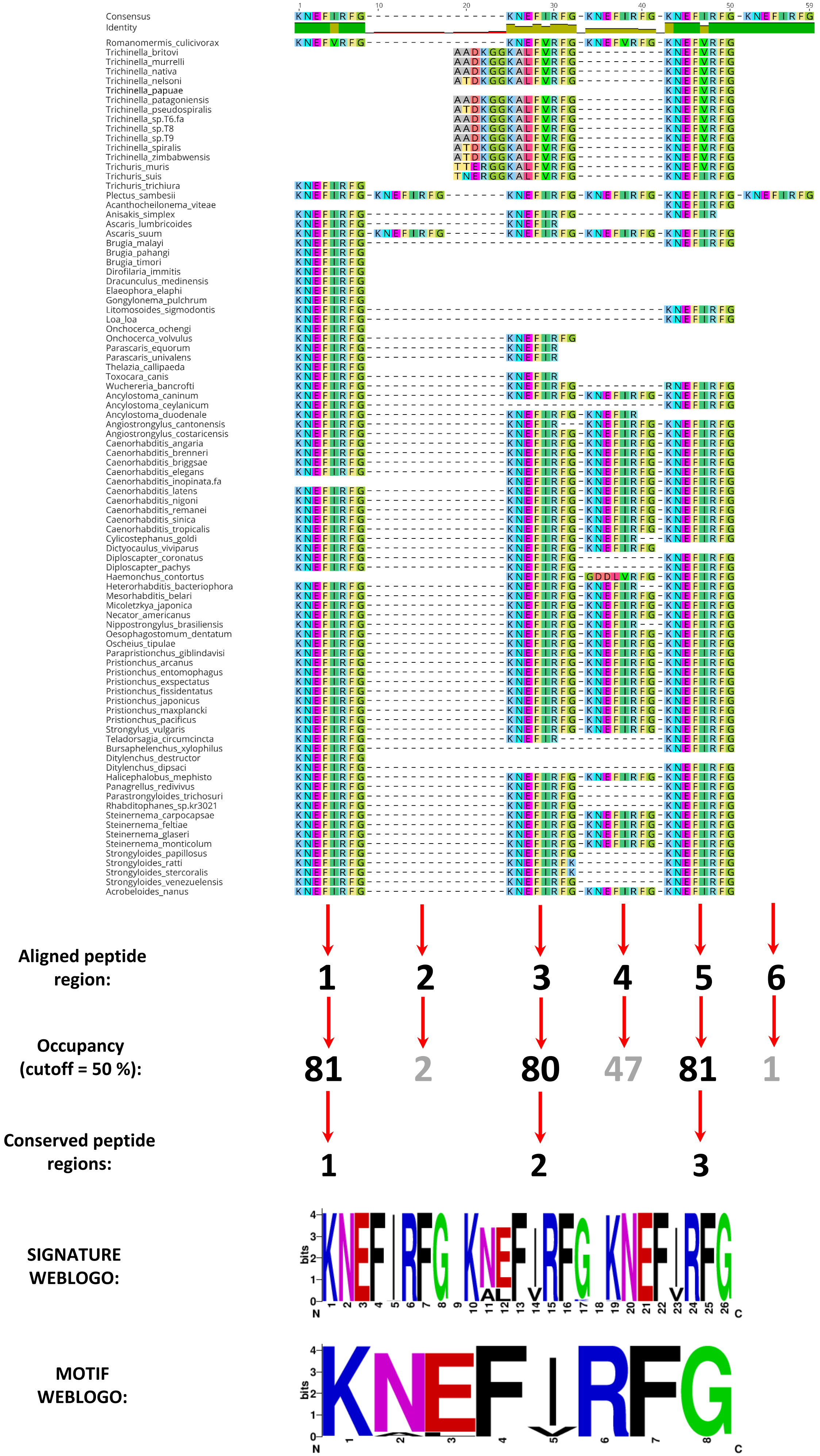

FLP-9 peptide alignment

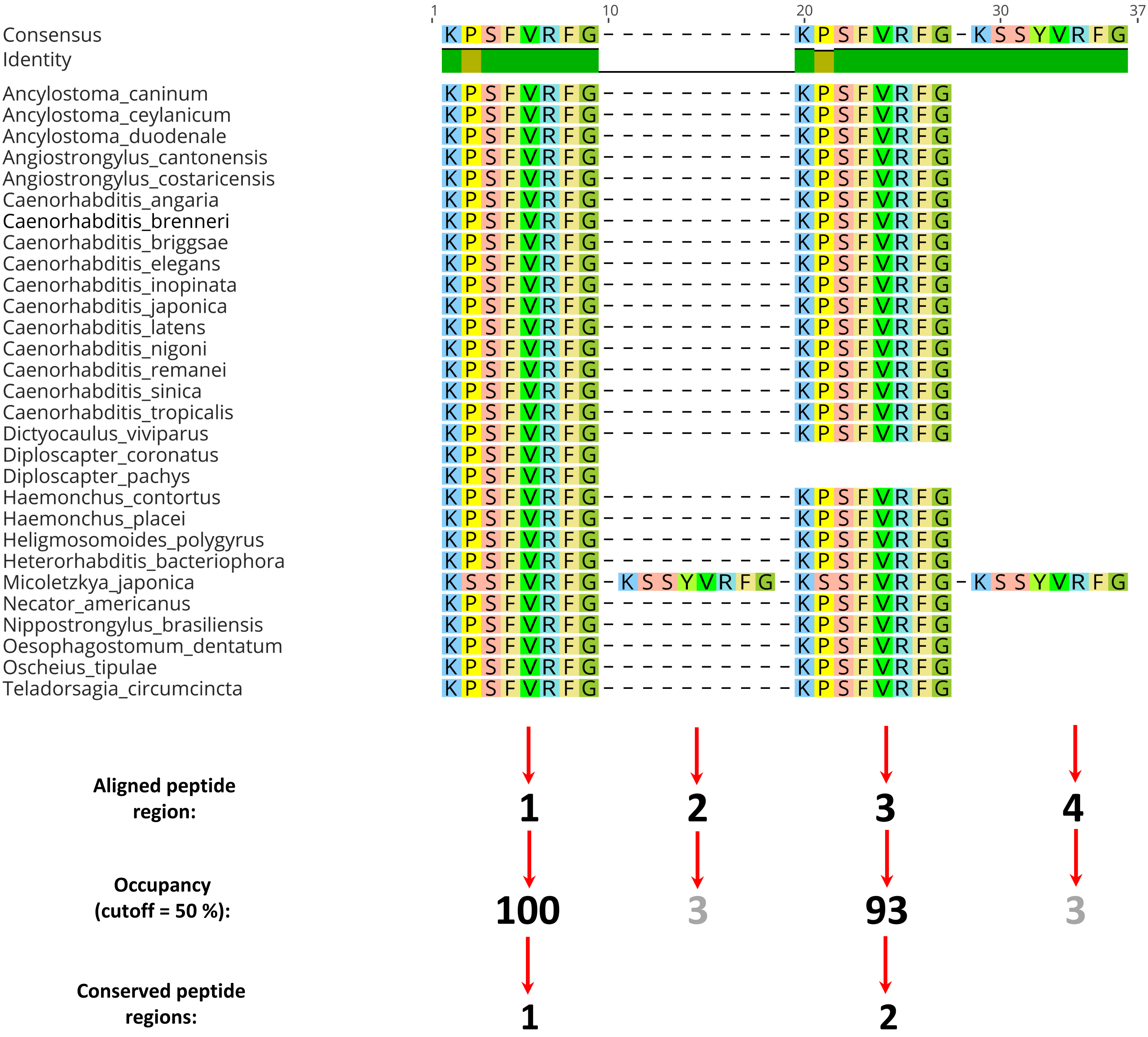

SIGNATURE WEBLOGO:

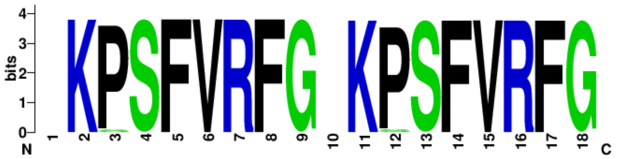

MOTIF WEBLOGO:

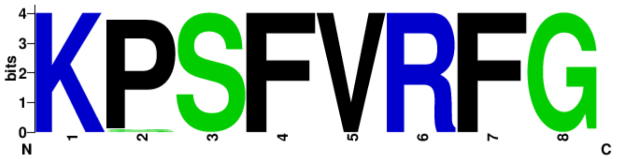

FLP-10 peptide alignment

	Sequence (positions 1–16)
Consensus	QAASQTKSRSGYIRFG
Identity	
Caenorhabditis_angaria	----QGKQRSGYIRFG
Caenorhabditis_brenneri	----QTKSRSGYIRFG
Caenorhabditis_briggsae	----QTKSRSGYIRFG
Caenorhabditis_elegans	----QPKARSGYIRFG
Caenorhabditis_inopinata	----QTKARSGYIRFG
Caenorhabditis_japonica	----QTKARSGYIRFG
Caenorhabditis_latens	----QTKSRSGYIRFG
Caenorhabditis_nigoni	----QTKSRSGYIRFG
Caenorhabditis_remanei	----QTKSRSGYIRFG
Caenorhabditis_sinica	----QTKSRSGYIRFG
Caenorhabditis_tropicalis	----QTKSRSGYIRFG
Diploscapter_coronatus	QAASANGMRSGYIRFG
Diploscapter_pachys	QAASANGMRSGYIRFG

Aligned peptide region: **1**

Occupancy (cutoff = 50 %): **100**

Conserved peptide regions: **1**

SIGNATURE/MOTIF WEBLOGO:

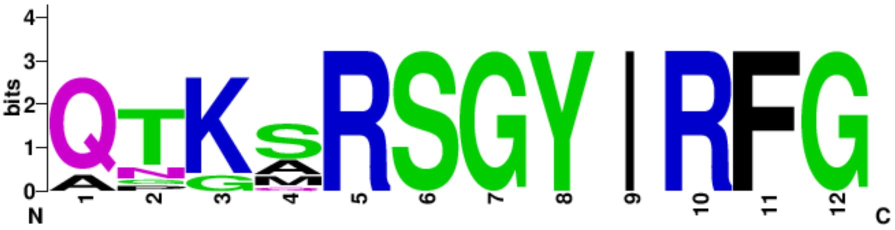

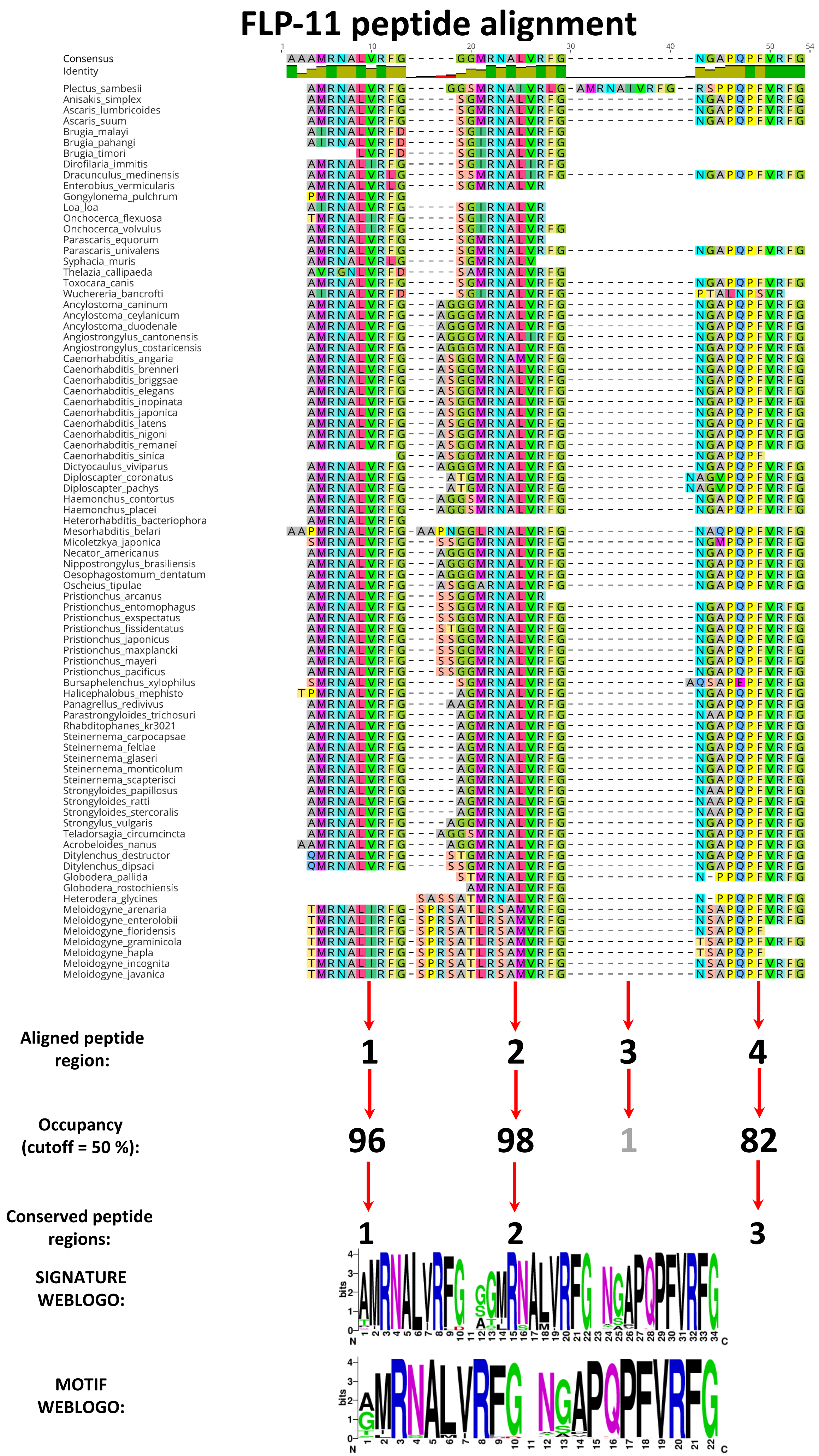
FLP-11 peptide alignment
Consensus
Identity
Plectus_sambesii
Anisakis_simplex
Ascaris_lumbricoides
Ascaris_suum
Brugia_malayi
Brugia_pahangi
Brugia_timori
Dirofilaria_immitis
Dracunculus_medinensis
Enterobius_vermicularis
Gongylonema_pulchrum
Loa_loa
Onchocerca_flexuosa
Onchocerca_volvulus
Parascaris_equorum
Parascaris_univalens
Syphacia_muris
Thelazia_callipaeda
Toxocara_canis
Wuchereria_bancrofti
Ancylostoma_caninum
Ancylostoma_ceylanicum
Ancylostoma_duodenale
Angiostrongylus_cantonensis
Angiostrongylus_costaricensis
Caenorhabditis_angaria
Caenorhabditis_brenneri
Caenorhabditis_briggsae
Caenorhabditis_elegans
Caenorhabditis_inopinata
Caenorhabditis_japonica
Caenorhabditis_latens
Caenorhabditis_nigoni
Caenorhabditis_remanei
Caenorhabditis_sinica
Dictyocaulus_viviparus
Diploscapter_coronatus
Diploscapter_pachys
Haemonchus_contortus
Haemonchus_placei
Heterorhabditis_bacteriophora
Mesorhabditis_belari
Micoletzkya_japonica
Necator_americanus
Nippostrongylus_brasiliensis
Oesophagostomum_dentatum
Oscheius_tipulae
Pristionchus_arcanus
Pristionchus_entomophagus
Pristionchus_exspectatus
Pristionchus_fissidentatus
Pristionchus_japonicus
Pristionchus_maxplancki
Pristionchus_mayeri
Pristionchus_pacificus
Bursaphelenchus_xylophilus
Halicephalobus_mephisto
Panagrellus_redivivus
Parastrongyloides_trichosuri
Rhabditophanes_kr3021
Steinernema_carpocapsae
Steinernema_feltiae
Steinernema_glaseri
Steinernema_monticolum
Steinernema_scapterisci
Strongyloides_papillosus
Strongyloides_ratti
Strongyloides_stercoralis
Strongylus_vulgaris
Teladorsagia_circumcincta
Acrobeloides_nanus
Ditylenchus_destructor
Ditylenchus_dipsaci
Globodera_pallida
Globodera_rostochiensis
Heterodera_glycines
Meloidogyne_arenaria
Meloidogyne_enterolobii
Meloidogyne_floridensis
Meloidogyne_graminicola
Meloidogyne_hapla
Meloidogyne_incognita
Meloidogyne_javanica
Aligned peptide region:
1 2 3 4
Occupancy (cutoff = 50 %):
96 98 1 82
Conserved peptide regions:
1 2 3
SIGNATURE WEBLOGO:
MOTIF WEBLOGO:

FLP-12 peptide alignment

	1–11
Consensus	R-NKFEFIRFG
Identity	
Acanthocheilonema_viteae	R-NKFEFIRFG
Anisakis_simplex	R-NKFEFIRFG
Ascaris_lumbricoides	R-NKFEFIRFG
Ascaris_suum	R-NKFEFIRFG
Brugia_malayi	R-NKFEFIRFG
Brugia_pahangi	R-NKFEFIRFG
Brugia_timori	R-NKFEFIRFG
Dirofilaria_immitis.txt	R-NKFEFIRFG
Dracunculus_medinensis	R-NKFEFIRFG
Elaeophora_elaphi	R-NKFEFIRFG
Enterobius_vermicularis	R-NKFEFIRFG
Gongylonema_pulchrum	R-NKFEFIRFG
Litomosoides_sigmodontis	R-NKFEFIRFG
Loa_loa	R-NKFEFIRFG
Onchocerca_flexuosa	R-NKFEFIRFG
Onchocerca_ochengi	R-NKFEFIRFG
Onchocerca_volvulus	R-NKFEFIRFG
Parascaris_univalens	R-NKFEFIRFG
Syphacia_muris	R--KFEFIRFG
Thelazia_callipaeda	R-NKFEFIRFG
Toxocara_canis	R-NKFEFIRFG
Wuchereria_bancrofti	R-NKFEFIRFG
Ancylostoma_caninum	R-NKFEFIRFG
Ancylostoma_ceylanicum	R-NKFEFIRFG
Ancylostoma_duodenale	R-NKFEFIRFG
Angiostrongylus_costaricensis	R-NKFEFIRFG
Caenorhabditis_angaria	R-NKFEFIRFG
Caenorhabditis_brenneri	R-NKFEFIRFG
Caenorhabditis_briggsae	R-NKFEFIRFG
Caenorhabditis_elegans	R-NKFEFIRFG
Caenorhabditis_inopinata	R-NKFEFIRFG
Caenorhabditis_japonica	R-NKFEFIRFG
Caenorhabditis_latens	R-NKFEFIRFG
Caenorhabditis_nigoni	R-NKFEFIRFG
Caenorhabditis_sinica	R-NKFEFIRFG
Caenorhabditis_tropicalis	R-NKFEFIRFG
Dictyocaulus_viviparus	R-NKFEFIRFG
Diploscapter_coronatus	R-NKFEFIRFG
Diploscapter_pachys	R-NKFEFIRFG
Haemonchus_contortus	R-NKFEFIRFG
Haemonchus_placei	R-NKFEFIRFG
Heligmosomoides_polygyrus	R-NKFEFIRFG
Heterorhabditis_bacteriophora	R-NKFEFIRFG
Mesorhabditis_belari	R-NKFEFIRFG
Micoletzkya_japonica	R-NKFEFIRFG
Necator_americanus	R-NKFEFIRFG
Nippostrongylus_brasiliensis	R-NKFEFIRFG
Oesophagostomum_dentatum	R-NKFEFIRFG
Parapristionchus_giblindavisi	R-NKFEFIRFG
Pristionchus_arcanus	R-NKFEFIRFG
Pristionchus_entomophagus	R-NKFEFIRFG
Pristionchus_exspectatus	R-NKFEFIRFG
Pristionchus_fissidentatus	R-NKFEFIRFG
Pristionchus_japonicus	R-NKFEFIRFG
Pristionchus_maxplancki	R-NKFEFIRFG
Pristionchus_mayeri	R-NKFEFIRFG
Pristionchus_pacificus	R-NKFEFIRFG
Teladorsagia_circumcincta	R-NKFEFIRFG
Bursaphelenchus_xylophilus	R-NKFEFIRFG
Halicephalobus_mephisto	R-NKFEFIRFG
Panagrellus_redivivus	R-NKFEFIRFG
Parastrongyloides_trichosuri	R-NKFEFIRFG
Rhabditophanes_sp.KR3021	R-NKFEFIRFG
Steinernema_carpocapsae	R-NKFEFIRFG
Steinernema_feltiae	R-NKFEFIRFG
Steinernema_glaseri	R-NKFEFIRFG
Steinernema_monticolum	R-NKFEFIRFG
Steinernema_scapterisci	R-NKFEFIRFG
Strongyloides_papillosus	R-NKFEFIRFG
Strongyloides_ratti	R-NKFEFIRFG
Strongyloides_stercoralis	R-NKFEFIRFG
Strongyloides_venezuelensis	R-NKFEFIRFG
Acrobeloides_nanus	R-NKFEFIRFG
Ditylenchus_destructor	R-NKFEFIRFG
Ditylenchus_dipsaci	R-NKFEFIRFG
Globodera_pallida	K-NKFEFIRFG
Globodera_rostochiensis	K-NKFEFIRFG
Heterodera_glycines	K-NKFEFIRFG
Meloidogyne_arenaria	KNNKFEFIRFG
Meloidogyne_enterolobii	KNNKFEFIRFG
Meloidogyne_floridensis	KNNKFEFIRFG
Meloidogyne_graminicola	KNNKFEFIRFG
Meloidogyne_hapla	KNNKFEFIRFG
Meloidogyne_incognita	KNNKFEFIRFG
Meloidogyne_javanica	KNNKFEFIRFG

Aligned peptide region: 1

Occupancy (cutoff = 50 %): 100

Conserved peptide regions: 1

SIGNATURE/MOTIF WEBLOGO:

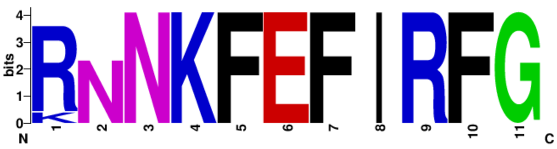

FLP-13 peptide alignment

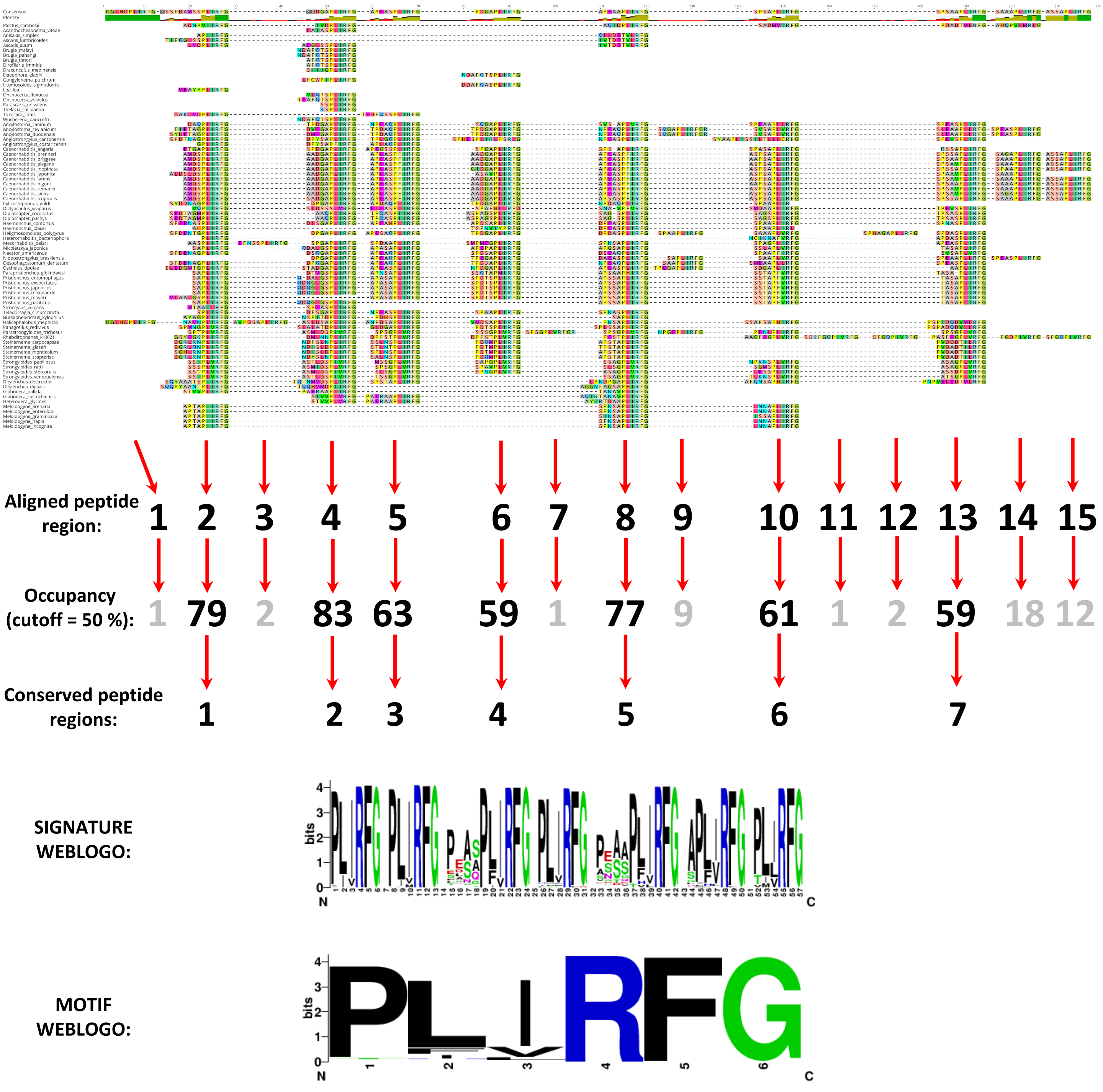

FLP-14 peptide alignment

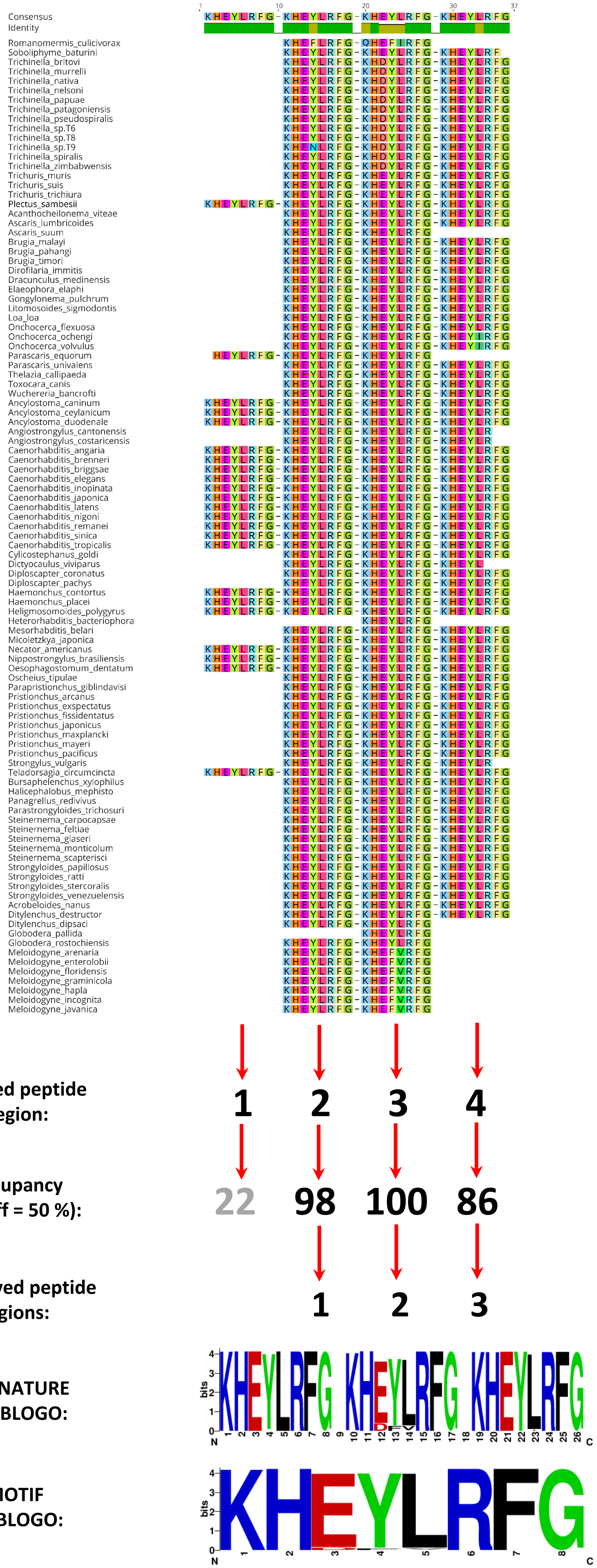

FLP-15 peptide alignment

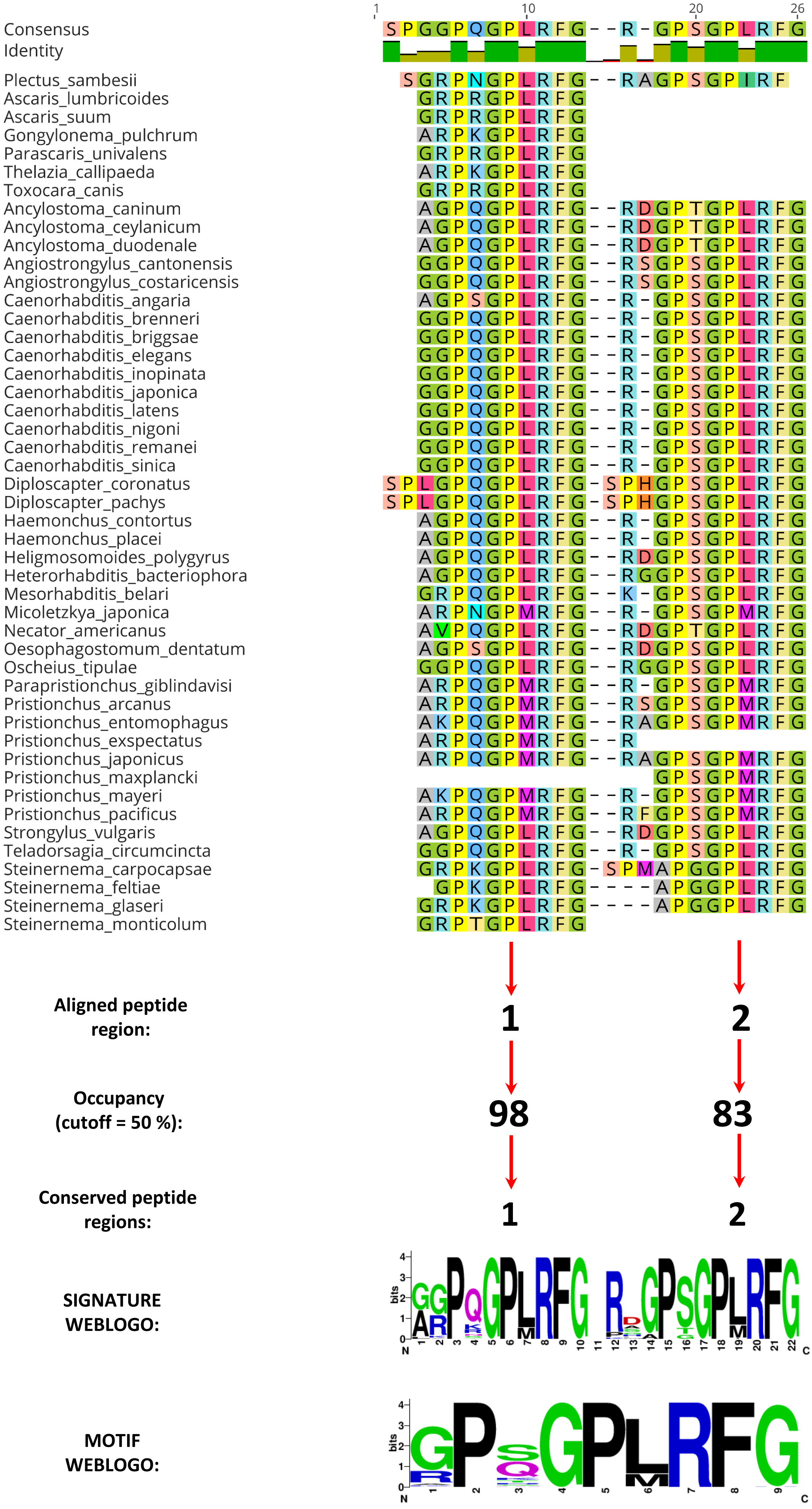

FLP-16 peptide alignment

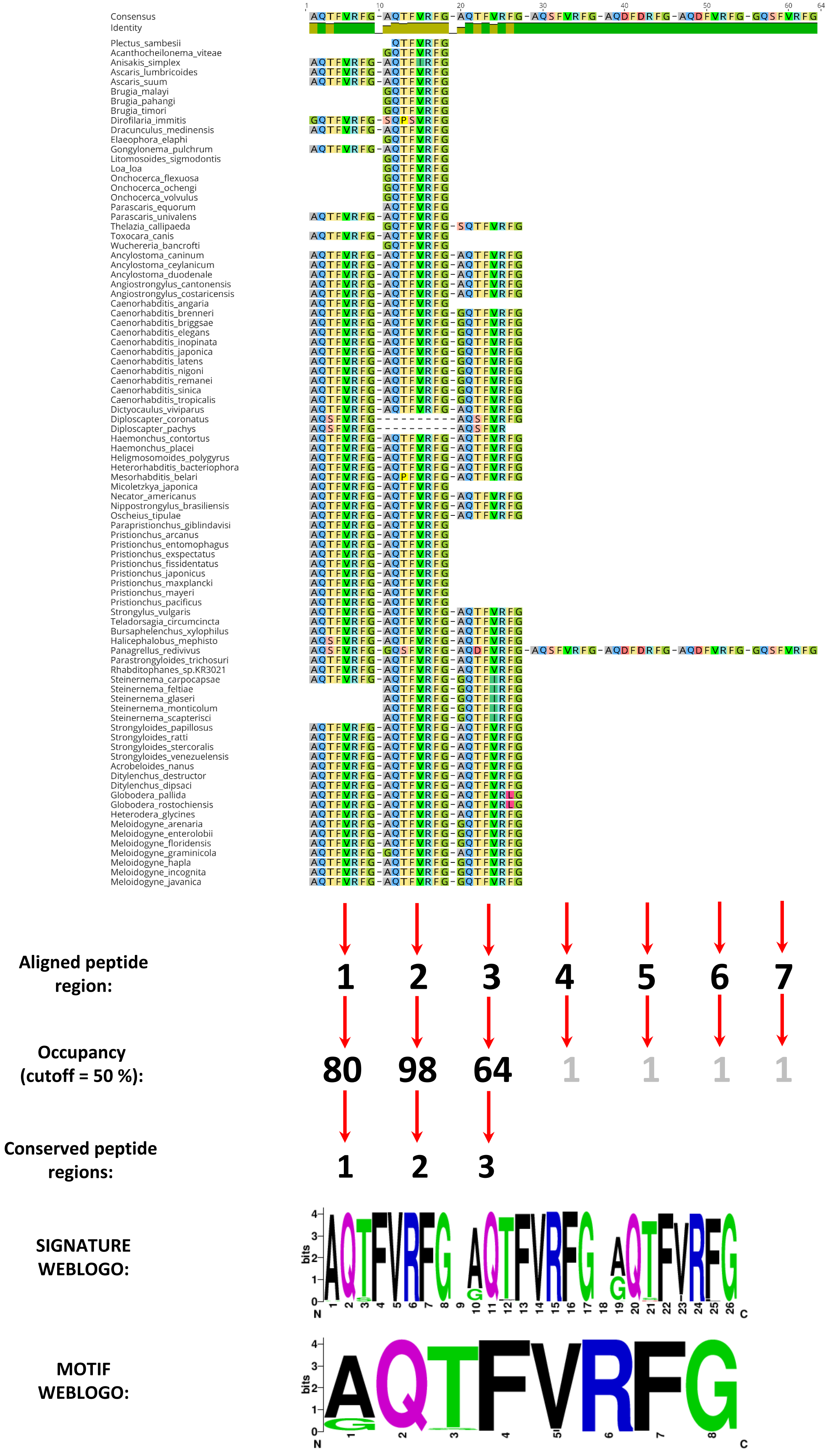

FLP-17 peptide alignment

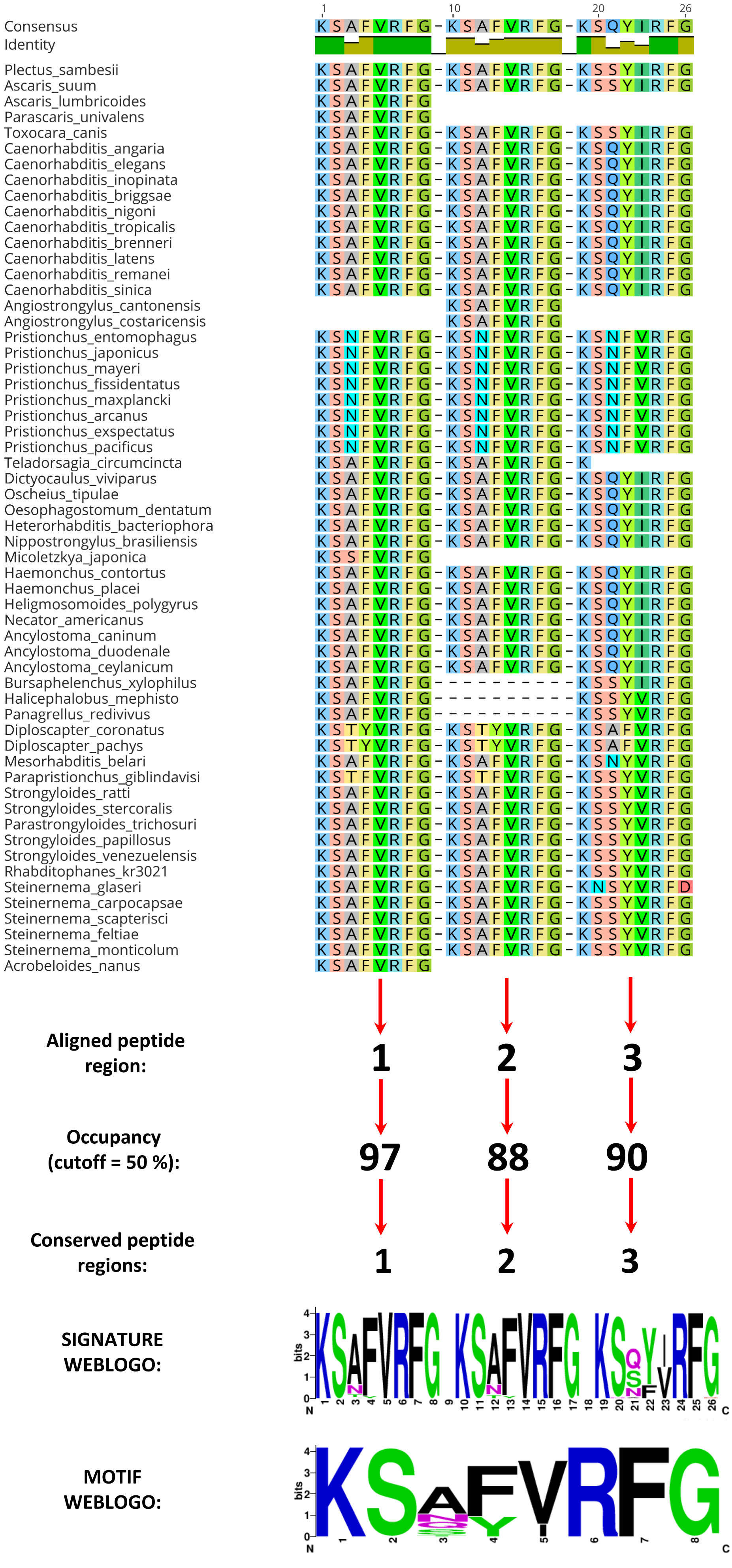

FLP-18 peptide alignment

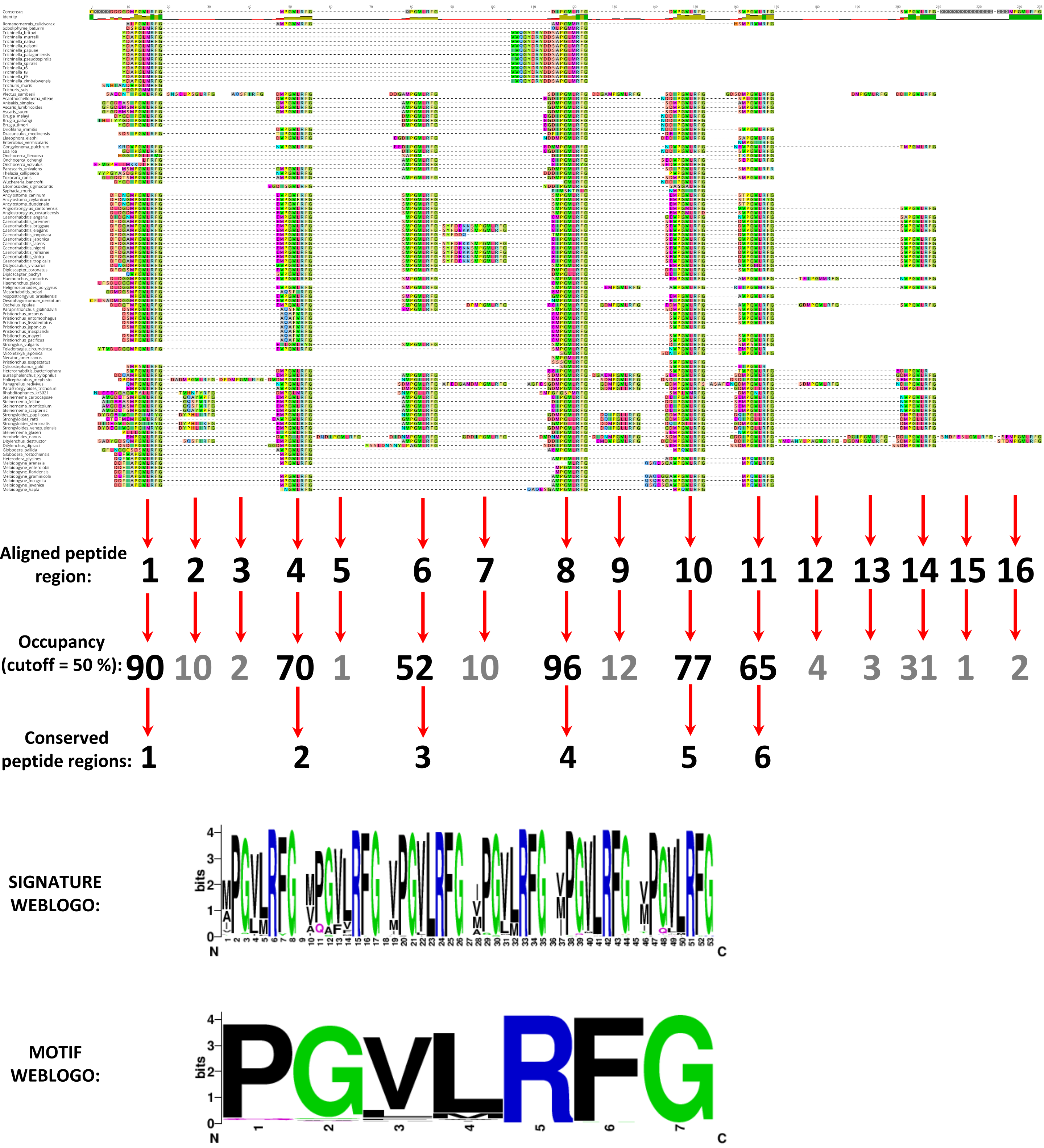

FLP-19 peptide alignment

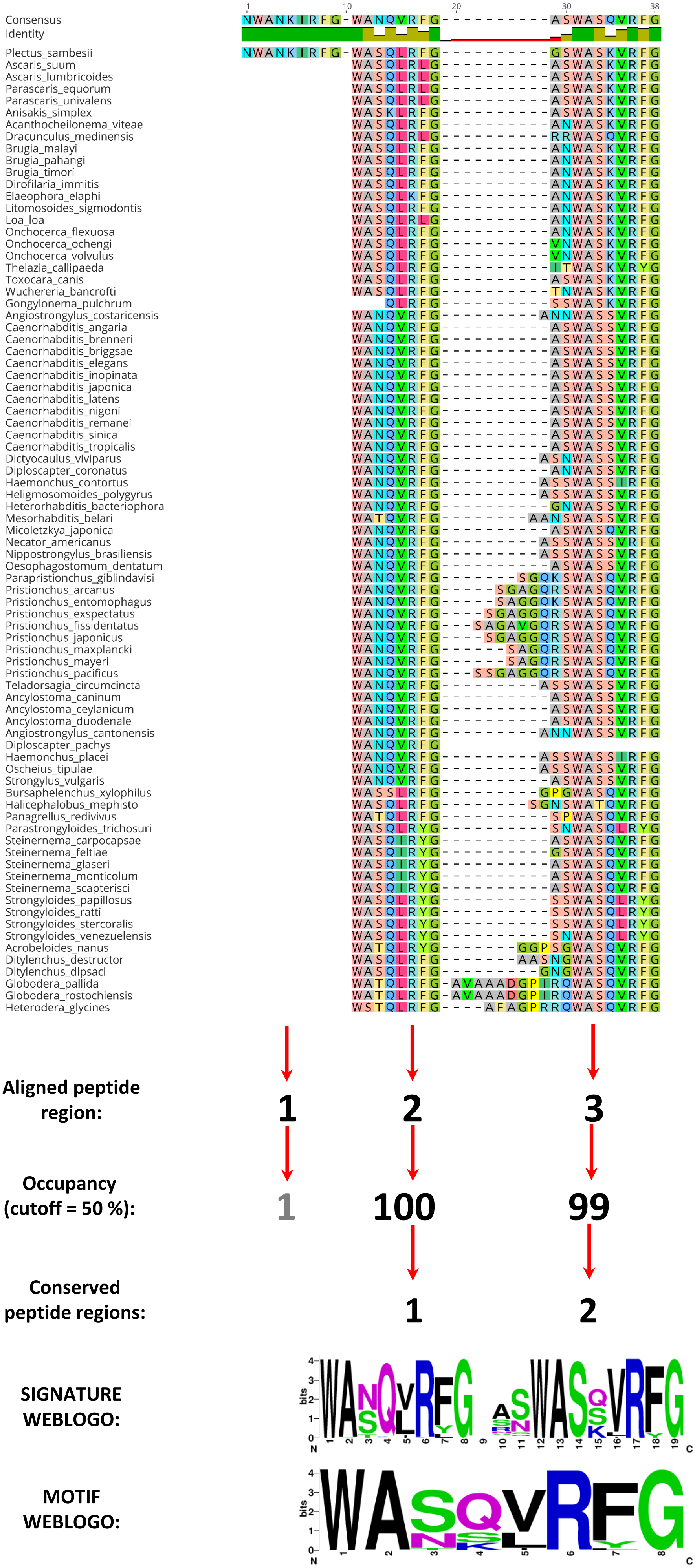

FLP-20 peptide alignment

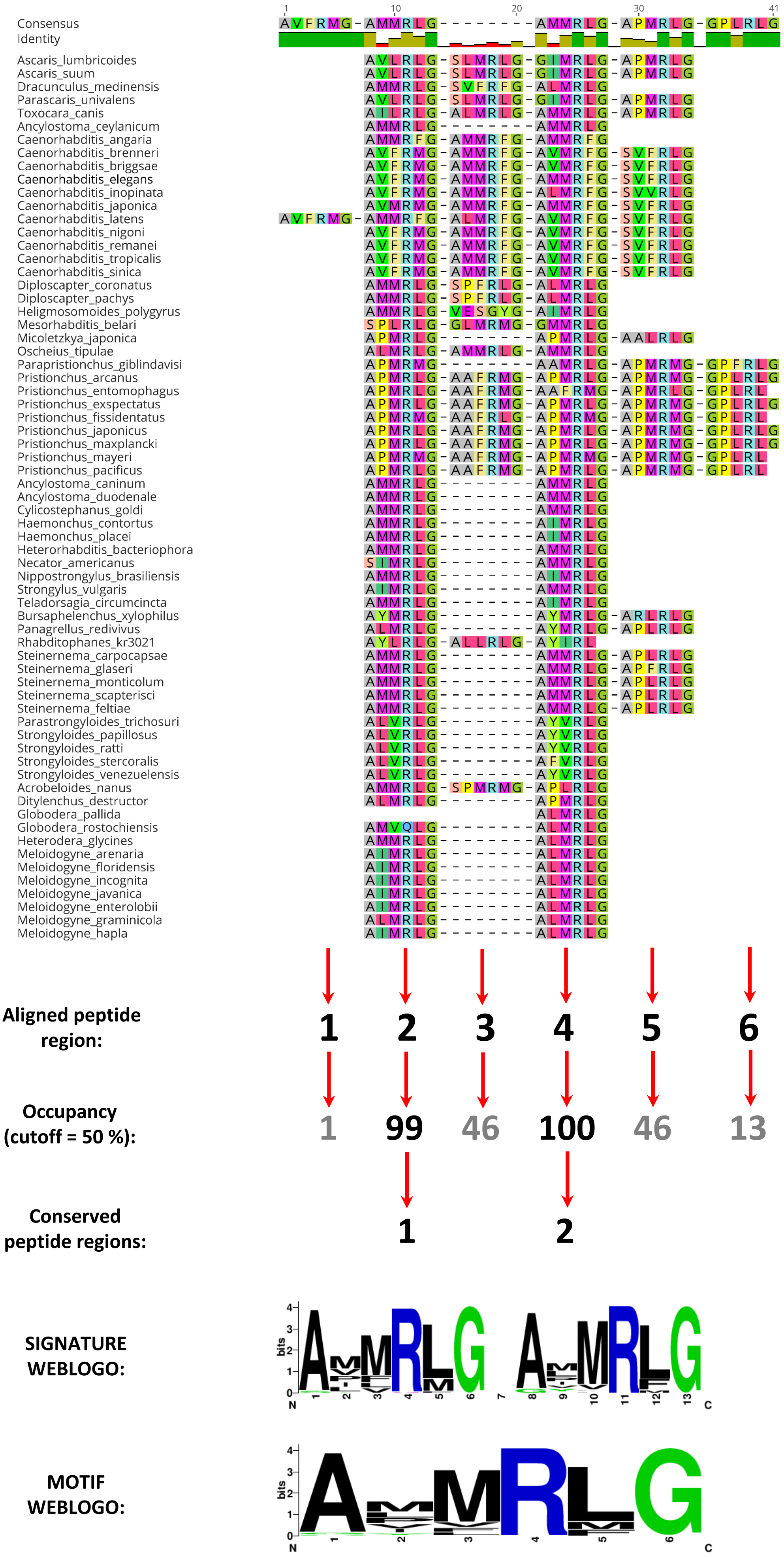

FLP-21 peptide alignment

	Sequence (positions 1–12)
Consensus	GXGLGPRPLRFG
Identity	
Plectus_sambesii	GLGPRPLRFG
Ascaris_lumbricoides	GLGPRPLRFG
Brugia_malayi	ALGPRPLRFG
Brugia_pahangi	ALGPRPLRFG
Dracunculus_medinensis	NGLGLRPLRFG
Litomosoides_sigmodontis	ALGPRPLRFG
Loa_loa	ALGPRPLRFG
Onchocerca_volvulus	APGPRPLRFG
Acanthocheilonema_viteae	ALGPRPLRFG
Anisakis_simplex	GLGPRPLRFG
Ascaris_suum	GLGPRPLRFG
Brugia_timori	ALGPRPLRFG
Dirofilaria_immitis	ARGPRPLRFG
Elaeophora_elaphi	ALGPRPLRFG
Gongylonema_pulchrum	ALGPRPLRFG
Onchocerca_flexuosa	APGPRPLRFG
Onchocerca_ochengi	APGPRPLRFG
Parascaris_equorum	GLGPRPLRFG
Parascaris_univalens	GLGPRPLRFG
Thelazia_callipaeda	ALRPRPLRFG
Toxocara_canis	GLGPRPLRFG
Wuchereria_bancrofti	ALGPRPLRFG
Ancylostoma_ceylanicum	GLGPRPLRFG
Angiostrongylus_costaricensis	GLGPRPLRFG
Caenorhabditis_angaria	GLGPRPLR
Caenorhabditis_brenneri	GLGPRPLRFG
Caenorhabditis_briggsae	GLGPRPLRFG
Caenorhabditis_elegans	GLGPRPLRFG
Caenorhabditis_inopinata	GLGPRPLRFG
Caenorhabditis_japonica	GLGPRPLRFG
Caenorhabditis_latens	GLGPRPLRFG
Caenorhabditis_remanei	GLGPRPLRFG
Diploscapter_coronatus	GLGPRPLRFG
Haemonchus_contortus	GLGPRPLRFG
Haemonchus_placei	GLGPRPLRFG
Heligmosomoides_polygyrus	GLGPRPLRFG
Oscheius_tipulae	GLGPRPLRFG
Ancylostoma_duodenale	GLGPRPLRFG
Angiostrongylus_cantonensis	GLGPRPLRFG
Caenorhabditis_sinica	GLGPRPLRFG
Caenorhabditis_tropicalis	GLGPRPLRFG
Cylicostephanus_goldi	GLGPRPLRFG
Diploscapter_pachys	GLGPRPLRFG
Heterorhabditis_bacteriophora	GLGPRPLRFG
Mesorhabditis_belari	GHGPRPLRFG
Necator_americanus	GLGPRPLRFG
Teladorsagia_circumcincta	GLGPRPLRFG
Bursaphelenchus_xylophilus	GMGPRPLRFG
Halicephalobus_mephisto	ALGPRPLRFG
Panagrellus_redivivus	ALGPRPLRFG
Parastrongyloides_trichosuri	AVGPRPLRFG
Steinernema_carpocapsae	GLGPRPLRFG
Steinernema_feltiae	GLGPRPLRFG
Steinernema_glaseri	GLGPRPLRFG
Steinernema_monticolum	GLGPRPLRFG
Steinernema_scapterisci	GLGPRPLRFG
Strongyloides_papillosus	AVGPRPLRFG
Strongyloides_ratti	AVGPRPLRFG
Strongyloides_stercoralis	AVGPRPLRFG
Strongyloides_venezuelensis	AVGPRPLRFG
Acrobeloides_nanus	GLGPRPLRFG
Ditylenchus_destructor	GLGMGPRPLRFG
Globodera_rostochiensis	GSLGPRPLRFG
Ditylenchus_dipsaci	GLGPRPLRFG

Aligned peptide region: 1

Occupancy (cutoff = 50 %): 100

Conserved peptide regions: 1

SIGNATURE/MOTIF WEBLOGO:

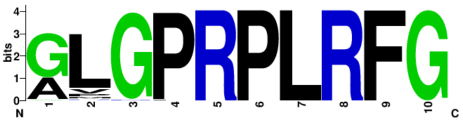

FLP-22 peptide alignment

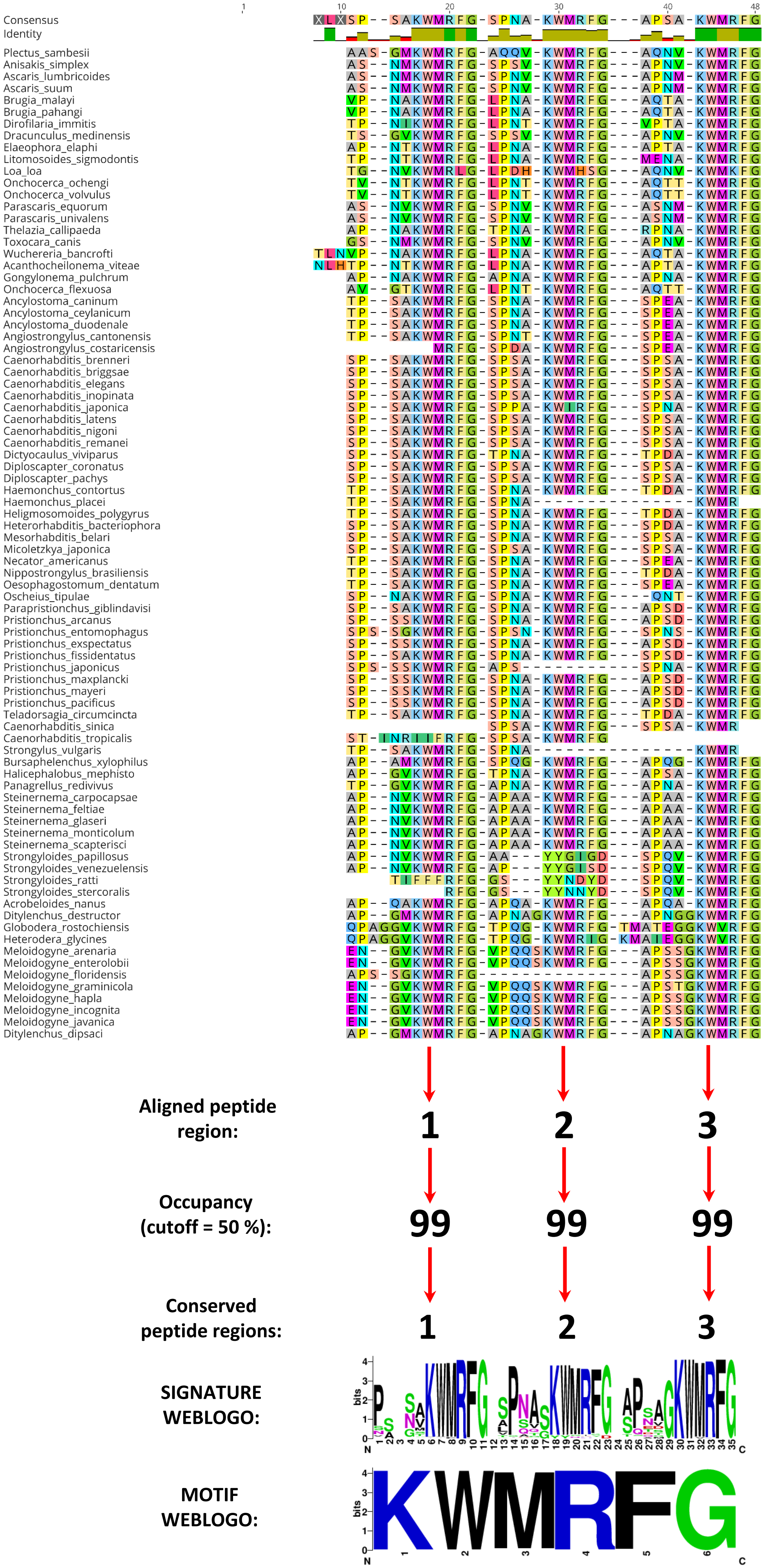

FLP-23 peptide alignment

Sequence	Alignment (positions 1–20)
Consensus	AV---PFERVPGQQDFLRFG
Identity	
Brugia_malayi	AALESIIRANTGRQDFLRFG
Onchocerca_ochengi	TALDSIIQANTGRQDFLRFG
Parascaris_univalens	WSLPNVLHDNIGRQDFLRFG
Wuchereria_bancrofti	AALENIIRANTGRQDFLRFG
Ancylostoma_ceylanicum	AAPPNYGRQDFLRFG
Caenorhabditis_brenneri	AV---PFERVPGQQDFLRFG
Caenorhabditis_briggsae	SV---PFERAPGQQDFLRFG
Caenorhabditis_inopinata	AA---PFERAVGQQDFLRFG
Caenorhabditis_latens	AV---PFERVPGQQDFLRFG
Caenorhabditis_nigoni	SV---PFERAPGQQDFLRFG
Caenorhabditis_remanei	AV---PFERVPGQQDFLRFG
Caenorhabditis_sinica	AV---PFERVPGQQDFLRFG
Caenorhabditis_tropicalis	AV---PFERVPGQQDFLRFG
Heterorhabditis_bacteriophora	AIRSFDNTKNYIAQDFLRFG
Pristionchus_arcanus	ADPSAWAGREDFLRFG
Pristionchus_exspectatus	ADPSAWAGREDFLRFG
Pristionchus_fissidentatus	SAPAAFSPYREEFLRFG
Caenorhabditis_elegans	AV---PFERVVGQQDFLRFG
Caenorhabditis_japonica	PKFSAFQDFLRFG
Mesorhabditis_belari	CCGLNKVKDFLRFG
Oscheius_tipulae	VNTLSIVANTKDFLRFG
Parapristionchus_giblindavisi	APTKLRLFQDFLRFG
Pristionchus_maxplancki	LLQ-SDTNPILFLQDFLRFG

Aligned peptide region: 1

Occupancy (cutoff = 50 %): 100

Conserved peptide regions: 1

SIGNATURE/MOTIF WEBLOGO:

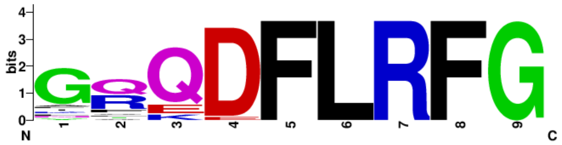

FLP-24 peptide alignment

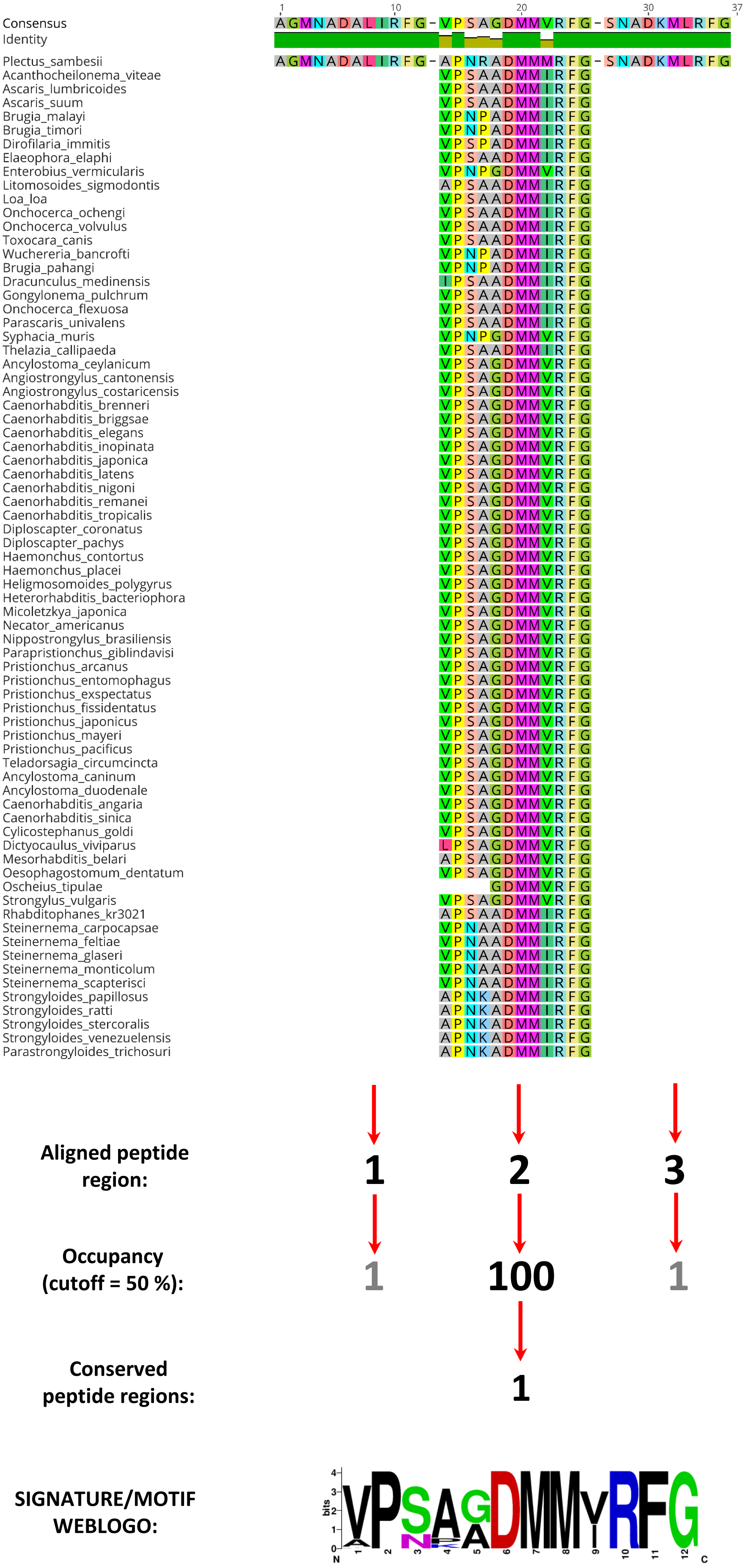

FLP-25 peptide alignment

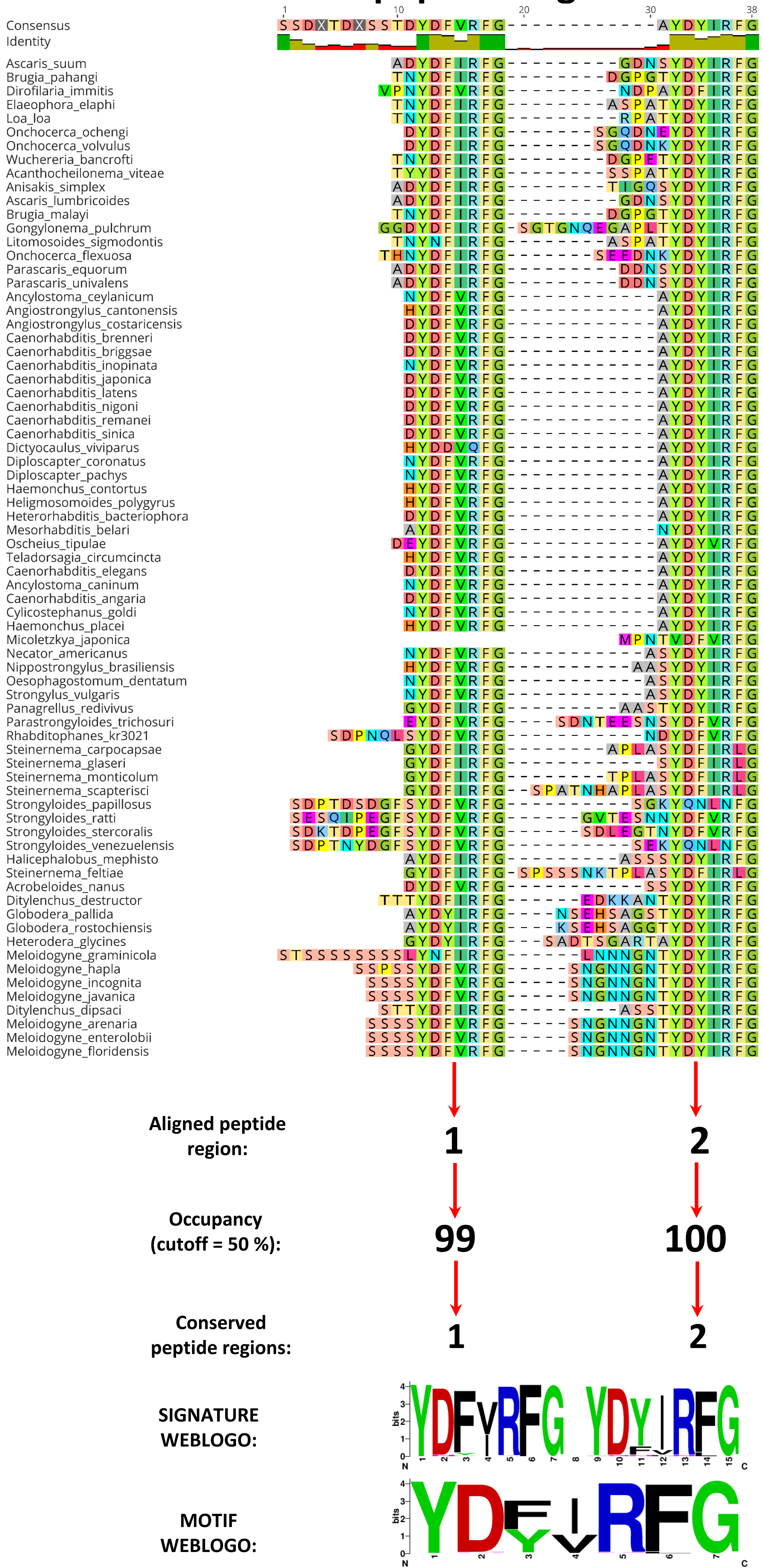

FLP-26 peptide alignment

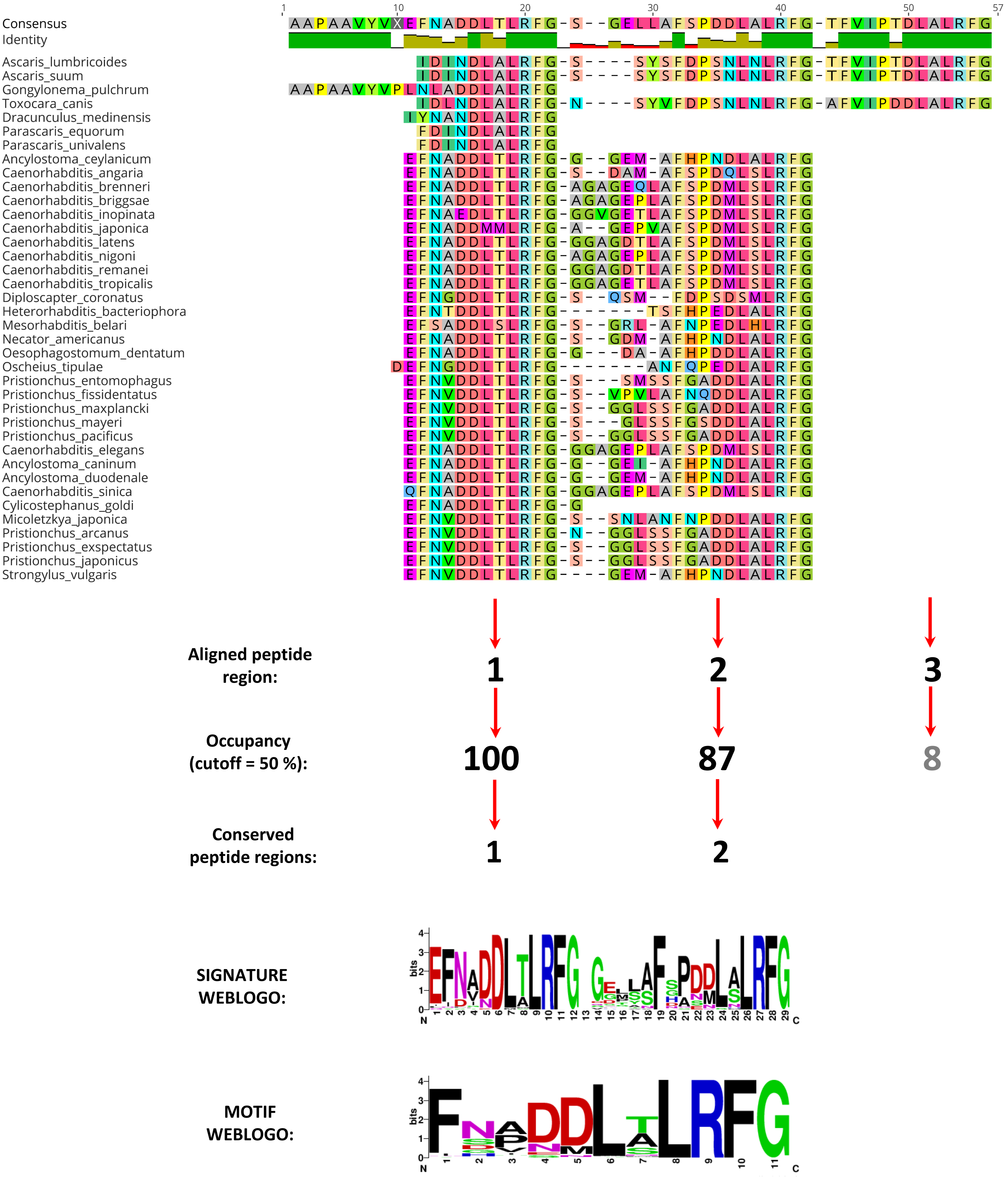

FLP-27 peptide alignment

	1 ... 10 ... 12
Consensus	SQTLGGRMRFG
Identity	
Ancylostoma_caninum	TMGGRMRFG
Ancylostoma_ceylanicum	TMGGRMRFG
Caenorhabditis_brenneri	GLGGRMRFG
Caenorhabditis_briggsae	GLGGRMRFG
Caenorhabditis_inopinata	GLGGRMRFG
Caenorhabditis_latens	GLGGRMRFG
Caenorhabditis_nigoni	GLGGRMRFG
Caenorhabditis_remanei	GLGGRMRFG
Caenorhabditis_sinica	GLGGRMRFG
Micoletzkya_japonica	TLGGRMRFG
Necator_americanus	TMGGRMRFG
Oesophagostomum_dentatum	TMGGRMRFG
Oscheius_tipulae	SMGGRMRFG
Pristionchus_arcanus	SQALGGRMRFG
Pristionchus_entomophagus	TQTIGGRMRFG
Pristionchus_exspectatus	SQALGGRMRFG
Pristionchus_fissidentatus	TQTLGGRMRFG
Pristionchus_japonicus	SQTLGGRMRFG
Pristionchus_maxplancki	SQTLGGRMRFG
Pristionchus_pacificus	SQALGGRMRFG
Caenorhabditis_elegans	GLGGRMRFG

Aligned peptide region: **1**

Occupancy (cutoff = 50 %): **100**

Conserved peptide regions: **1**

SIGNATURE/MOTIF WEBLOGO:

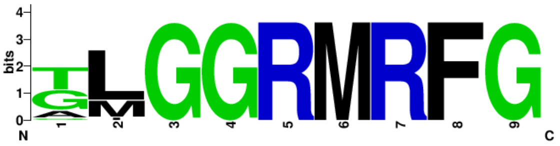

FLP-28 peptide alignment

	Sequence (positions 1–10)
Consensus	APNRILMRFG
Identity	
Ascaris_lumbricoides	APNKILMRFG
Ascaris_suum	APNKILMRFG
Parascaris_equorum	APNKILMRFG
Toxocara_canis	APNKILMRFG
Anisakis_simplex	APNKILMRFG
Dracunculus_medinensis	APNKILMRFG
Parascaris_univalens	APNKILMRFG
Ancylostoma_caninum	APNRILMRFG
Ancylostoma_ceylanicum	APNRILMRFG
Ancylostoma_duodenale	APNRILMRFG
Angiostrongylus_costaricensis	APNRILMRFG
Caenorhabditis_brenneri	APNRVLMRFG
Caenorhabditis_elegans	APNRVLMRFG
Caenorhabditis_briggsae	APNRVLMRFG
Caenorhabditis_inopinata	APNRVLMRFG
Caenorhabditis_japonica	APNRVLMRFG
Caenorhabditis_latens	APNRVLMRFG
Caenorhabditis_nigoni	APNRVLMRFG
Caenorhabditis_remanei	APNRVLMRFG
Caenorhabditis_tropicalis	APNRVLMRFG
Cylicostephanus_goldi	APNRILMRFG
Haemonchus_contortus	VPNRIFMRFG
Haemonchus_placei	VPNRIFMRFG
Heligmosomoides_polygyrus	APNRILMRFG
Micoletzkya_japonica	APNRVLMRFG
Oesophagostomum_dentatum	APNRILMRFG
Parapristionchus_giblindavisi	APNRVLMRFG
Pristionchus_arcanus	APSRVLMRFG
Pristionchus_entomophagus	APSRVLMRFG
Pristionchus_exspectatus	APSRVLMRFG
Pristionchus_fissidentatus	APSRVMMRFG
Pristionchus_japonicus	APSRVLMRFG
Pristionchus_maxplancki	APSRVLMRFG
Pristionchus_mayeri	APSRVLMRFG
Pristionchus_pacificus	APSRVLMRFG
Strongylus_vulgaris	APNRILMRFG
Angiostrongylus_cantonensis	APNRILMRFG
Caenorhabditis_angaria	APNRVLMRFG
Dictyocaulus_viviparus	APHRILMRFG
Diploscapter_coronatus	ASNRVLMRFG
Diploscapter_pachys	ASNRVLMRFG
Heterorhabditis_bacteriophora	APNRILMRFG
Mesorhabditis_belari	APNRILMRFG
Necator_americanus	APNRILMRFG
Nippostrongylus_brasiliensis	VPNRILMRFG
Oscheius_tipulae	APNRILMRFG
Teladorsagia_circumcincta	APNRIFMRFG
Parastrongyloides_trichosuri	APNRVMMRFG
Rhabditophanes_kr3021	APSRVMMRFG
Steinernema_carpocapsae	APNRILMRFG
Steinernema_feltiae	APNRILMRFG
Steinernema_monticolum	APNRILMRFG
Steinernema_scapterisci	APNRILMRFG
Strongyloides_ratti	APNRVMMRFG
Strongyloides_venezuelensis	APNRVMMRFG
Steinernema_glaseri	APNRILMRFG
Strongyloides_papillosus	APNRVMMRFG
Strongyloides_stercoralis	APNRVMMRFG

Aligned peptide region: 1

Occupancy (cutoff = 50 %): 100

Conserved peptide regions: 1

SIGNATURE/MOTIF WEBLOGO:

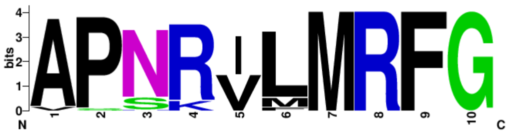

FLP-31 peptide alignment

	1										11
Consensus	L	Y	R	P	R	G	P	P	R	F	G
Identity											
Meloidogyne_arenaria	L	Y	R	P	R	G	P	P	R	F	G
Meloidogyne_javanica	L	Y	R	P	R	G	P	P	R	F	G
Meloidogyne_enterolobii	L	Y	R	P	R	G	P	P	R	F	G
Meloidogyne_floridensis	L	Y	R	P	R	G	P	P	R	F	G
Meloidogyne_graminicola	L	Y	R	P	R	G	P	P	R	F	G
Meloidogyne_hapla	L	Y	R	P	R	G	P	P	R	F	G
Meloidogyne_incognita	L	Y	R	P	R	G	P	P	R	F	G

Aligned peptide region: **1**

Occupancy (cutoff = 50 %): **100**

Conserved peptide regions: **1**

SIGNATURE/MOTIF WEBLOGO:

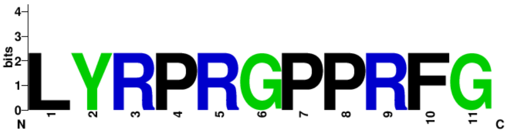

FLP-32 peptide alignment

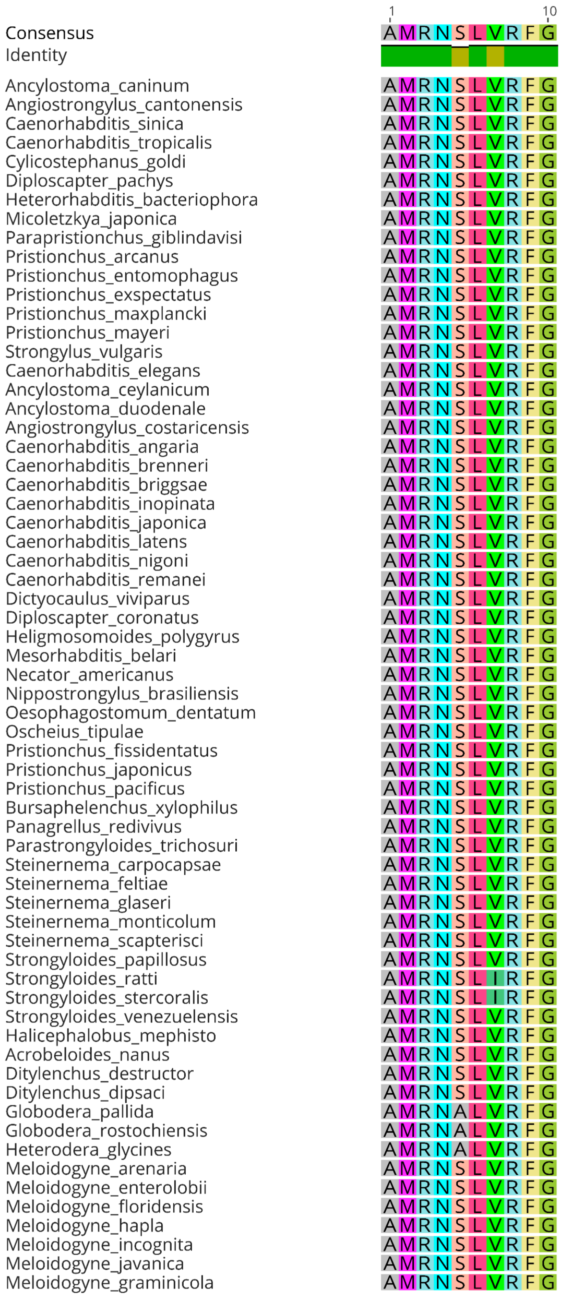

Aligned peptide region: **1**

Occupancy (cutoff = 50 %): **100**

Conserved peptide regions: **1**

SIGNATURE/MOTIF WEBLOGO:

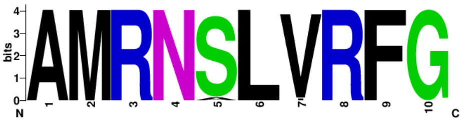

FLP-33 peptide alignment

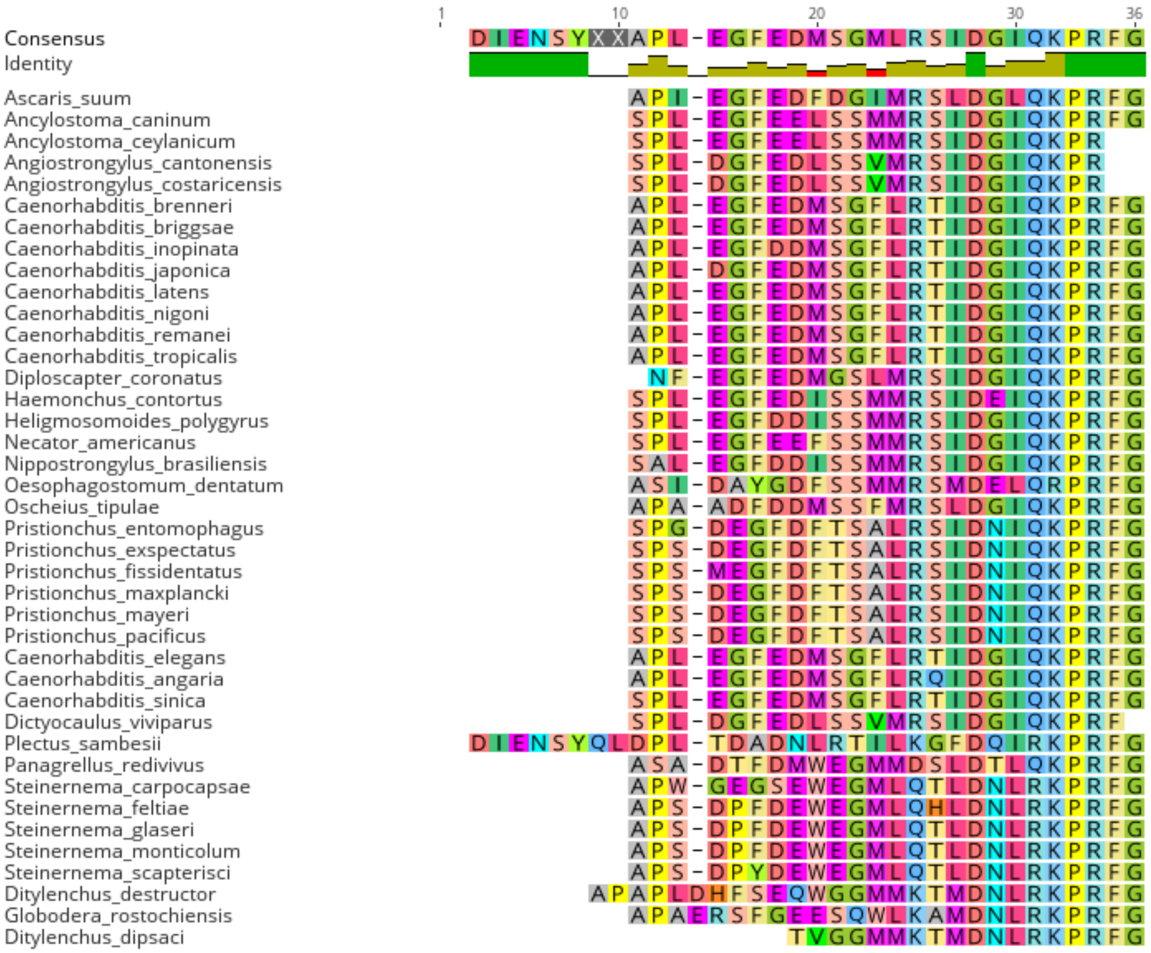

Aligned peptide region: **1**

Occupancy (cutoff = 50 %): **100**

Conserved peptide regions: **1**

SIGNATURE/MOTIF WEBLOGO:

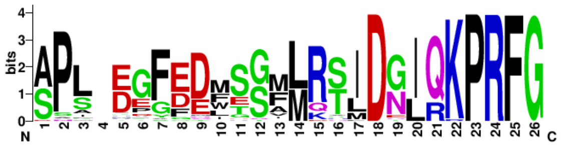

FLP-34 peptide alignment

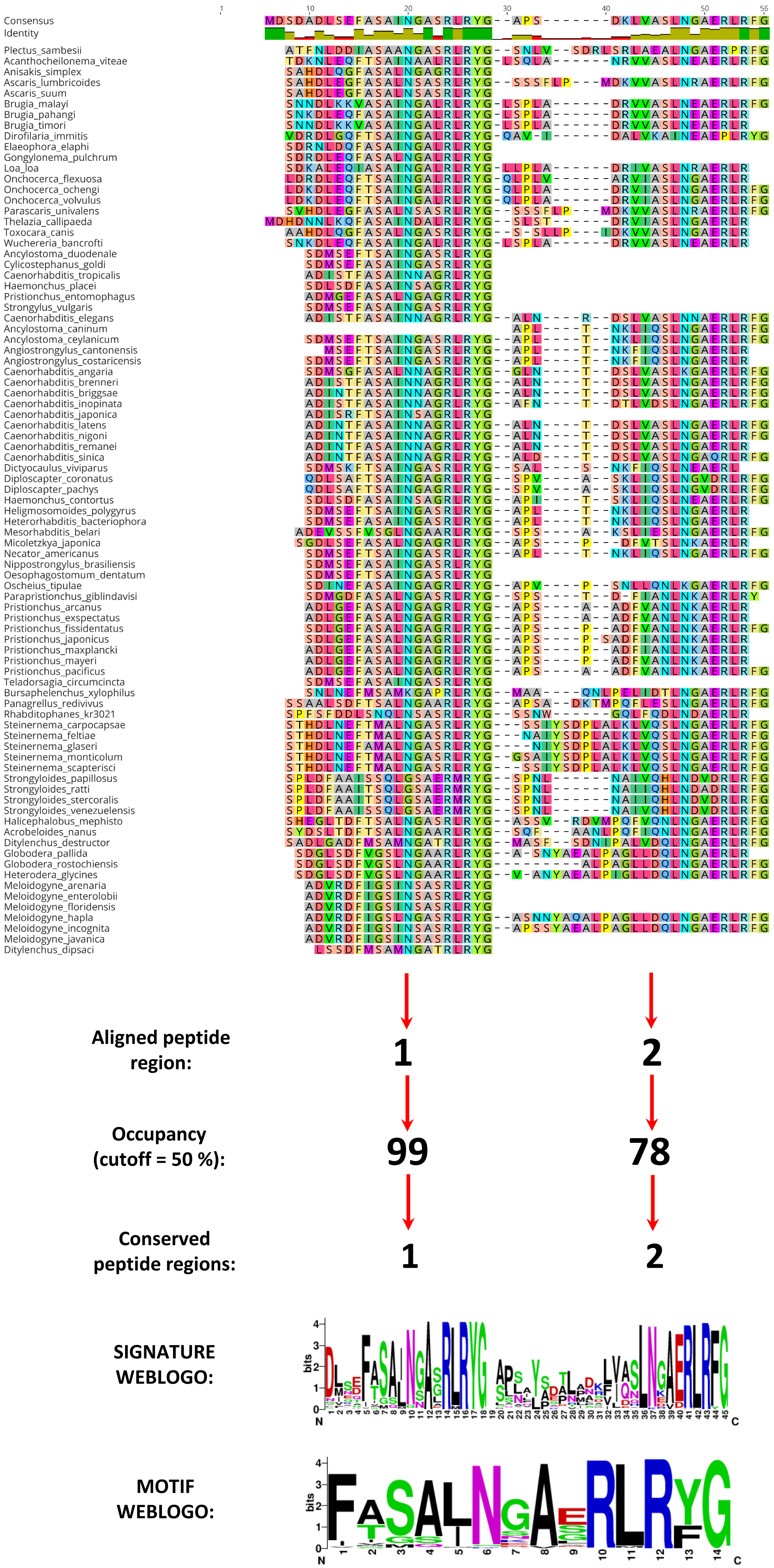

Supplement: Supplementary file 9 — Supplementary Material 9 [file 12864_2024_11111_MOESM9_ESM.pdf]
