# Supplementary material for: Exploitation of phylum-spanning omics resources reveals complexity in the nematode FLP signalling system and provides insights into flp-gene evolution
Source: BMC Genomics. 2024 Dec 19;25:1220. doi: 10.1186/s12864-024-11111-6 (PMC11658156; doi:10.1186/s12864-024-11111-6)
Supplement: Supplementary file 10 — Supplementary Material 10 [file 12864_2024_11111_MOESM10_ESM.docx]

**Supplementary Information**

**Supplementary Table 1: Genome and transcriptome datasets used in this study.**

**Supplementary Table 2: Putative *flp*-gene paralogues.**

**Supplementary File 1: FLP prepropeptide sequences identified via BLAST.**

**Supplementary File 2: FLP prepropeptide alignments.** Pages 1-28 represent an individual FLP prepropeptide alignment per page where page 1 represent FLP-1 and page 28 represents FLP-28, and each page between pages 29-32 represent FLP-31-34. Dashed black boxes highlight relatively conserved propeptide regions of FLP-1, -6, -12, -14 and -17 sequelogues.

**Supplementary Figure 1: FLP peptide alignments, signatures and motifs.** Note that only highly conserved aligned peptide regions (>50% occupancy) were used to generate the peptide signatures and motifs.

**Supplementary Figure 2: FLP-6 and FLP-17 prepropeptide alignment.** The conserved prepropeptide motif is highlighted by a red box and the black box (dashed line) highlights where this is not conserved.

**Supplementary Figure 3.** **AlphaFold derived structures of FLP prepropeptides.** Structures were downloaded (<https://alphafold.com>) and annotated. Red box indicates signal peptide; black arrows indicate conserved regions of the propeptide that are predicted to form alpha helices; * indicates a truncated predicted protein that does not encode a signal peptide; ** indicates an N-terminal extension of the predicted protein.

**Supplementary Figure 4.** **Lifestage and tissue-specific *flp*-gene expression profiles.** Heatmaps generated by mapping mean *flp* transcript Z-scores from lifestage and/or tissue-specific RNA-seq libraries. Y-axis denotes *flp*-gene. X-axis denotes lifestages or tissue types. Unannotated genes or those falling below the expression inclusion threshold of 1.5 TPM were omitted. *Trichuris muris*: life stages include second stage larvae (L2), third stage larvae (L3), adult female (AF), adult male (AM), and mixed adult (AMix Sex); tissues include adult female anterior (AF Ant), adult female posterior (AF Post), adult male anterior (AM Ant), adult male posterior (AM Post). *Ascaris suum*: tissues include anterior intestine, female intestine, female pharynx, female head, male head, male intestine, male pharynx, mid intestine, ovary, posterior intestine, seminal vesicle, testis, uterus, whole intestine, whole worm. *Dirofilaria immitis*: tissues include adult female body wall (AF Bodywall), adult female head (AF Head), adult female intestine (AF Intestine), adult female uterus (AF Uterus). *Brugia malayi*: life stages include eggs and embryos, microfilariae (MF), stage three larvae (L3), stage four larvae (L4), adult female (AF) and adult male (AM). *Onchocerca volvulus*: life stages include eggs and embryos, microfilariae (MF), stage two larvae (L2), stage three larvae (L3), adult female (AF), adult male (AM). *Ancylostoma caninum*: life stages include eggs, stage one larvae (L1), stage two larvae (L2), activated stage three larvae (L3 (A)), non-activated stage three larvae (L3 (NA)), untreated stage three larvae (L3 (UT)), adult female and adult male. *Dictyocaulus viviparus*: life stages include egg, stage one larvae (L1), mixed stage one and two larvae (L1+L2), stage two larvae (L2), stage three larvae (L3), mixed stage five larvae and adult females (L5AF), mixed stage five larvae and adult males (L5AM), mixed gender stage five larvae L5(Mixed), mixed stage five larvae and adults L5(Adult), hypobiotic larvae (LHyp), adult female (AF), adult male (AM); *Haemonchus contortus*: life stages include eggs, stage one larvae (L1), stage four larvae (L4), adult female (AF), adult male (AM), adult female gut (AF gut). *Teladorsagia circumcinta*: life stages include stage three larvae (L3), adult female, adult male. *Bursaphelenchus xylophilus*: life stages include mixed embryo, stage two larvae and 3 hour pooled male and females (L2+3h mixed), stage two larvae and 6 hour pooled male and females (L2+6h mixed), stage two larvae and pooled male and females (L2 mixed), stage three dispersal juvenile (S3 DJ), dauer, stage three larvae pooled male and female (L3 mixed), stage four larvae pooled male and female (L4 mixed), adult female, adult male, mixed propagative. *Strongyloides stercoralis*: life stages include post free-living stage one larvae (PFL L1), post parasitic stage one larvae (PP L1), post parasitic stage three larvae (PP L3), activated stage three larvae (L3+), infective stage three larvae (L3i), free-living females (FL Females), parasitic females (P Females). *Globodera pallida*: life stages include egg, stage two juveniles (J2), 7 days post infection (7dpi), 14 days post infection (14dpi), 21 days post infection (21dpi), 28 days post infection (28dpi), 35 days post infection (35dpi) and adult male (AM); *Meloidogyne incognita*: life stages include egg, stage two juvenile larvae (J2), stage three juvenile larvae (J3), stage four juvenile larvae (J4), adult female (AF). All datasets used are detailed in Supplementary Table 1.

**Supplementary Figure 5. Average *flp* transcripts per million values derived from lifestage and tissue specific RNASeq datasets.**  Each data point (grey circle) represents a specific *flp* TPM for a lifestage or tissue. All datasets used are detailed in Supplementary Table 1.
