# Supplementary material for: Exploitation of phylum-spanning omics resources reveals complexity in the nematode FLP signalling system and provides insights into flp-gene evolution
Source: BMC Genomics. 2024 Dec 19;25:1220. doi: 10.1186/s12864-024-11111-6 (PMC11658156; doi:10.1186/s12864-024-11111-6)
Supplement: Supplementary file 5 — Supplementary Material 5 [file 12864_2024_11111_MOESM5_ESM.pdf]

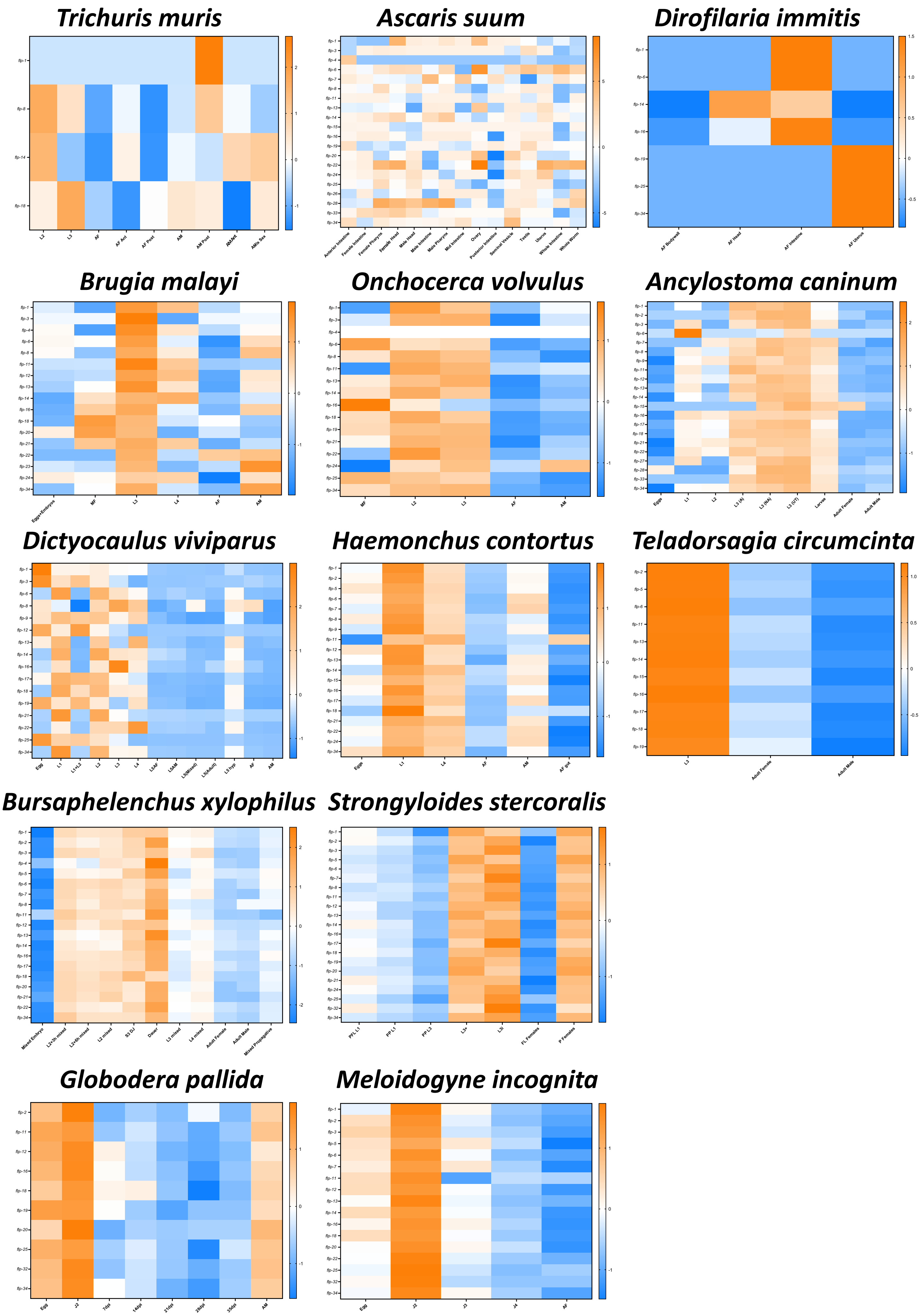
Trichuris muris
flp-1
flp-8
flp-14
flp-18
L2
L3
AF
AF Ant
AF Post
AM
AM Post
AMAnt
AMix Sex
Ascaris suum
Anterior Intestine
Female Intestine
Female Pharynx
Female Head
Male Head
Male Intestine
Male Pharynx
Mid Intestine
Ovary
Posterior Intestine
Seminal Vesicle
Testis
Uterus
Whole Intestine
Whole Worm
Dirofilaria immitis
flp-1
flp-6
flp-14
flp-16
flp-19
flp-25
flp-34
AF Bodywall
AF Head
AF Intestine
AF Uterus
Brugia malayi
Eggs+Embryos
MF
L3
L4
AF
AM
Onchocerca volvulus
MF
L2
L3
AF
AM
Ancylostoma caninum
Eggs
L1
L2
L3 (A)
L3 (NA)
L3 (UT)
Larvae
Adult Female
Adult Male
Dictyocaulus viviparus
Egg
L1
L1+L2
L2
L3
L4
L5AF
L5AM
L5(Mixed)
L5(Adult)
L5 hyp
AF
AM
Haemonchus contortus
Eggs
L1
L4
AF
AM
AF gut
Teladorsagia circumcinta
L3
Adult Female
Adult Male
Bursaphelenchus xylophilus
Mixed Embryo
L2+3h mixed
L2+6h mixed
L2 mixed
S3 DJ
Dauer
L3 mixed
L4 mixed
Adult Female
Adult Male
Mixed Propagative
Strongyloides stercoralis
PFL L1
PP L1
PP L3
L3+
L3i
FL Females
P Females
Globodera pallida
Egg
J2
7dpi
14dpi
21dpi
28dpi
35dpi
AM
Meloidogyne incognita
Egg
J2
J3
J4
AF
